# 2*H*-pyrazolo[3,4-*d*]pyrimidin-4-amine derivatives as novel selective fibroblast growth factor receptor 2 (FGFR2) inhibitors

**DOI:** 10.1080/14756366.2026.2647526

**Published:** 2026-03-30

**Authors:** Pinglian Wu, Zhaodi Tian, Weizhong Shen, Qiuju Xun, Yuan Tian, Huiqiong Li, Bowen Yang, Shaohua Chang, Weixue Huang, Zhen Wang, Ke Ding, Dawei Ma

**Affiliations:** ^a^ Chang-Kung Chuang Institute, School of Chemistry and Molecular Engineering, East China Normal University, Shanghai, China; ^b^ State Key Laboratory of Chemical Biology, Shanghai Institute of Organic Chemistry, Chinese Academy of Sciences, Shanghai, China; ^c^ KinoTeck Therapeutics Co., Ltd, Guangzhou City, China

**Keywords:** Antiproliferation, apoptosis, FGFR2, irreversible inhibitor, selectivity

## Abstract

Although FGFR2 is a well-validated oncogenic target, no selective FGFR2 inhibitors have been approved for clinical use. In this study, we report the discovery of 2*H*-pyrazolo[3,4-*d*]pyrimidin-4-amine derivative as novel, irreversible FGFR2 inhibitors. The optimal compound, **PLW559**, potently inhibited FGFR2 with an IC_50_ value of 13.59 nM and demonstrated exceptional selectivity over FGFR1, FGFR3, and FGFR4. Covalent binding to the target was confirmed by mass spectrometry. In cellular models, **PLW559** exhibited potent and selective antiproliferative effects against FGFR2-driven cancer cells, effectively suppressed downstream FGFR2 signalling and induced cancer cell apoptosis. Notably, it showed minimal activity in non-FGFR2-dependent cells. This work presents a new class of selective FGFR2 inhibitors based on a novel scaffold, offering promising lead compounds for the development of FGFR2-target therapies.

## 1. Introduction

Fibroblast growth factor receptor 2 (FGFR2), a member of the transmembrane receptor tyrosine kinase FGFR family (FGFR1, FGFR2, FGFR3 and FGFR4), activates critical cell survival and proliferation pathway upon ligand-induced dimerisation and autophosphorylation^1^^,^^2^. Oncogenic FGFR2 activation is implicated in various cancers and emerges as a potential therapeutic anti-cancer target^3–6^.

**Figure 1.**
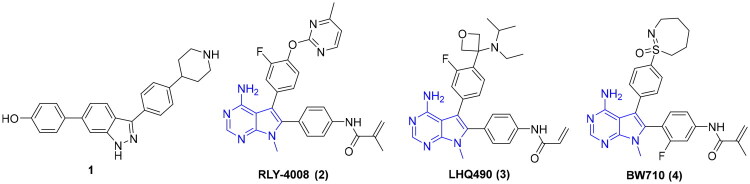
Chemical structures of selective FGFR2 inhibitors **1**–**4**.

Although approved pan-FGFR inhibitors (erdafitinib, pemigatinib, etc.) validate FGFR2 as a therapeutic target for cancer treatment^7–10^, their clinical utility is limited by off-isoform toxicity. Inhibition of FGFR1 frequently causes hyperphosphatemia, while FGFR4 inhibition can lead to diarrhea^11–16^. These limitations underscore the need for FGFR2-selective agents. Early selective inhibitors struggled to achieve high isoform selectivity. For instance, compound **1** (IC_50_ = 29 nM for FGFR2) showed only 13.4-fold selectivity over FGFR1^17^ (Figure 1). The field advanced significantly with the covalent inhibitor RLY-4008 (**2**)^18^^,^^19^, which targets FGFR2 with high potency (IC_50_ = 3 nM) and selectivity, demonstrating clinical efficacy without dose-limiting hyperphosphatemia or diarrhoea. Very recently, we also reported selective FGFR2 inhibitors by modification of compound **2**^20–22^. However, most reported selective FGFR2 inhibitors, including **2**–**4** (Figure 1), share a common 7-methyl-7*H*-pyrrolo[2,3-*d*]pyrimidin-4-amine core scaffold, highlighting a demand for novel chemotypes.

Herein, we report the discovery of 2*H*-pyrazolo[3,4-*d*]pyrimidin-4-amine derivatives as novel, selective FGFR2 inhibitors *via* a scaffold-hopping strategy. The representative compound, **PLW559,** potently inhibited FGFR2 (IC_50_ = 13.59 nM) with high selectivity over FGFR1, FGFR3 and FGFR4. It suppressed FGFR2 downstream signalling, selectively inhibited the proliferation of FGFR2-driven cells, and induced apoptosis of FGFR2-dependent cancer cells.

## 2. Results and discussion

### 2.1. Discovery of FGFR2 inhibitors with a novel core scaffold

**Figure 2.**
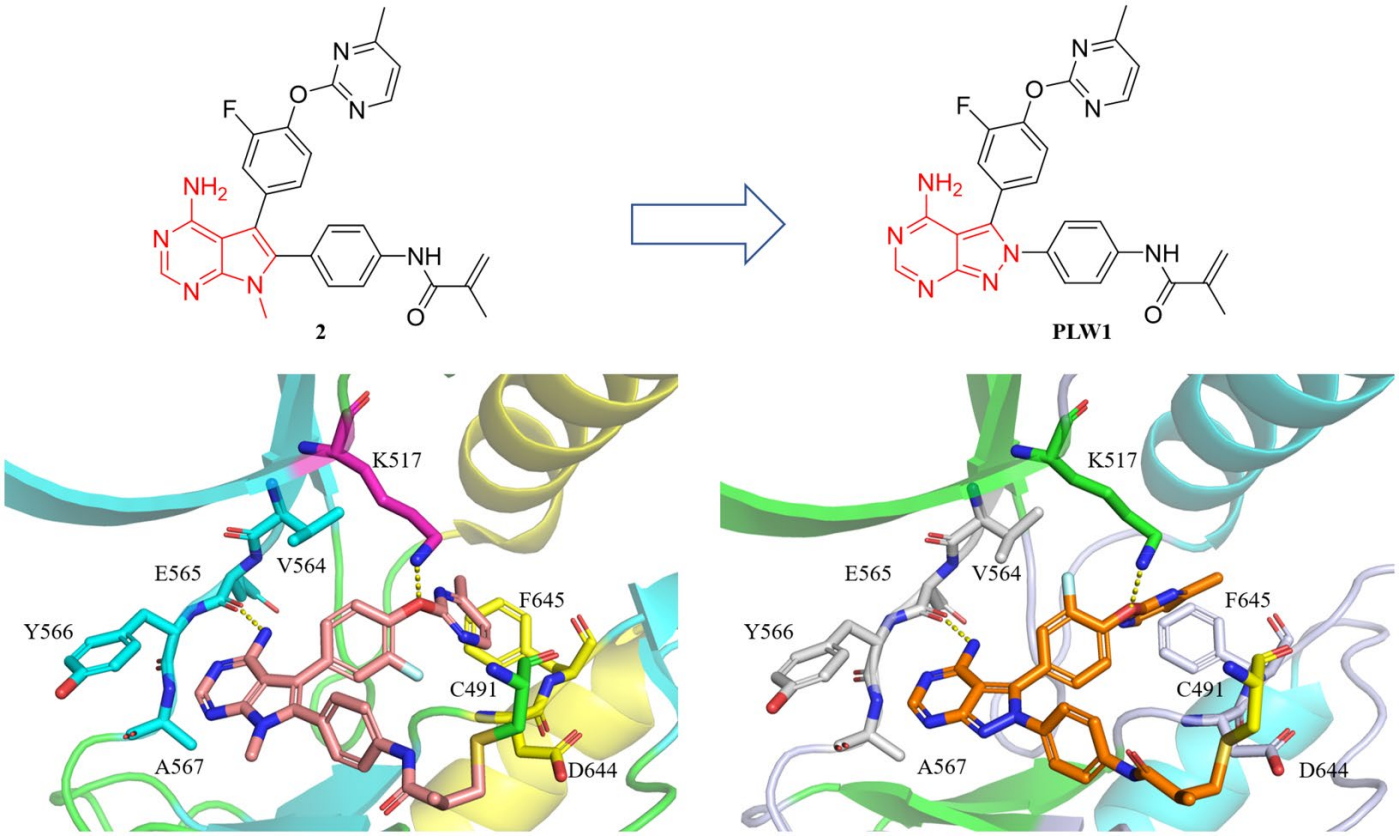
The binding mode of FGFR2 inhibitor **2** (PDB: 8STG) with FGFR2 and the predicted binding model of new FGFR2 inhibitor **PLW1** with FGFR2 are shown. Hydrogen bonds were indicated by yellow dashed lines.

The co-crystal structure of compound **2** bound to FGFR2 revealed three critical interactions: (1) a hydrogen bond between its 7-methyl-7*H*-pyrrolo[2,3-*d*]pyrimidin-4-amine core and the hinge residue Glu565; (2) a covalent bond formed by the methacrylamide warhead and Cys491 in the P-loop; (3) hydrophobic contacts between the pyrimidine moiety and the hydrophobic back pocket (Figure 2). Building on these structural insights, we proposed that the 7-methyl-7*H*-pyrrolo[2,3-*d*]pyrimidin-4-amine scaffold of compound **2** could be replaced with 2*H*-pyrazolo[3,4-*d*]pyrimidin-4-amine. This scaffold hopping strategy was designed to generate new selective FGFR2 inhibitors while preserving the essential hydrogen bond with Glu565 in the hinge region. Computational modelling of the resulting compound, **PLW1**, confirmed a binding pose similar to that of FGFR2, with the new core maintaining the key hydrogen bond to Glu565, preliminarily validating our design (Figure 2).

Based on the initial scaffold, compound **PLW1** was synthesised and evaluated for its antiproliferative activity against interleukin-3 (IL-3)-independent BaF3 cells expressing FGFR2 (BaF3-FGFR2) or FGFR1 (BaF3-FGFR1). **PLW1** inhibited the proliferation of BaF3-FGFR2 cells with an IC_50_ of 678.8 nM and showed good selectivity over BaF3-FGFR1 and parental BaF3 cells (IC_50_ > 1000 nM).

Using **PLW1** as the starting point, we replaced its methacrylamide warhead with various alternative Michael acceptors (**PLW2-6**), as different warheads can exhibit varying reactivity towards cysteine residue^23^. As summarised in Table 1, while **PLW2** and **PLW3** showed a significant loss of potency, **PLW4** through **PLW6** displayed improved antiproliferative activity against FGFR2-dependent cells. Among them, **PLW6** showed the most pronounced enhancement, with an IC_50_ of 54.9 nM against BaF3-FGFR2 cells, representing a greater than 10-fold improvement over **PLW1**. Subsequently, **PLW6** was selected as a new lead to investigate the effect of aryl substitutions at the *R*^2^ and *R*³ positions. Comparison of regioisomeric fluorine substitutions revealed that moving the fluorine from *R*^2^ to the *R*³ (**PLW7**) reduced activity by approximately 2-fold. Furthermore, removing the fluorine (**PLW8**) or replacing it with a methyl group (**PLW9**) also decreased potency. To further explore the hydrophobic back pocket, the 4-methylpyrimidine moiety was replaced with cycloalkyl groups, leading to the design of **PLW10** and **PLW11**. Although introducing a cyclobutyl (**PLW10**) or cyclohexyl (**PLW11**) group markedly enhanced potency, yielding IC_50_ values of 7.7 nM and 1.2 nM, respectively, both compounds also showed significant antiproliferative activity in BaF3-FGFR1 cells. We next modified the 4-methylpyrimidine scaffold by removing the methyl group or a nitrogen atom, resulting in compounds **PLW12–14**. Among these, **PLW14** (later designated **PLW559**) exhibited substantially enhanced activity against FGFR2-driven cells while showing no detectable inhibition of FGFR1-dependent proliferation. Based on these collective results, **PLW559** was selected for further comprehensive pharmacological characterisation.

**Table 1.** The antiproliferation efficacies of compounds **PLW1-14** against BaF3-FGFR1, BaF3-FGFR2 and parental BaF3 cell lines.^a^

Compd.	*R* ^1^	*R* ^2^	*R* ^3^	*R* ^4^	IC_50_ (nM)		
					BaF3-FGFR1	BaF3-FGFR2	BaF3
2	–	–	–	–	195.4 ± 62.7	1.3 ± 0.1	>1000
PLW1	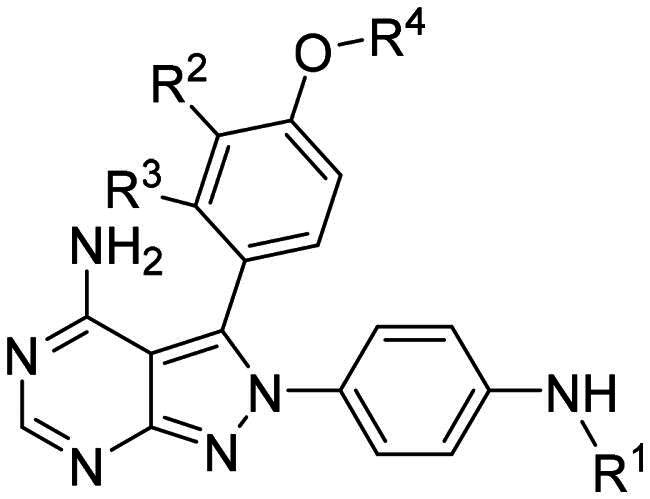	F	H	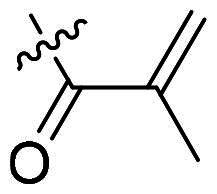	>1000	678.8 ± 0.1	>1000
PLW2	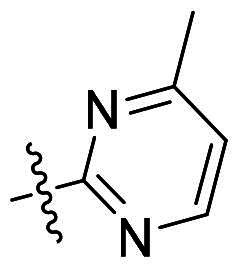	F	H	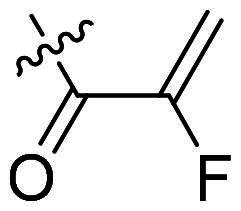	>1000	>1000	>1000
PLW3	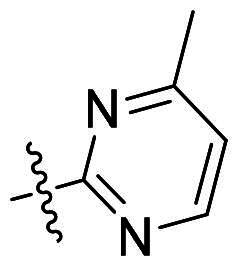	F	H	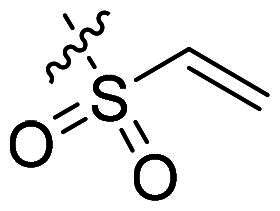	>1000	>1000	>1000
PLW4	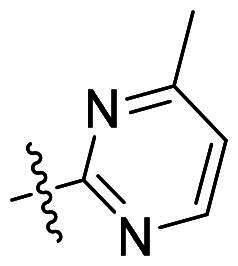	F	H	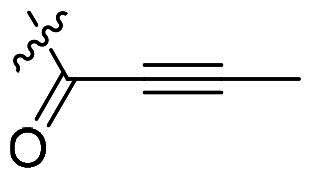	>1000	148.9 ± 56.6	>1000
PLW5	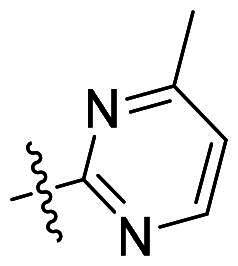	F	H	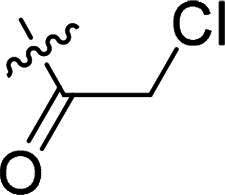	>1000	227.1 ± 75.3	>1000
PLW6	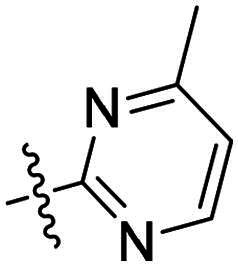	F	H	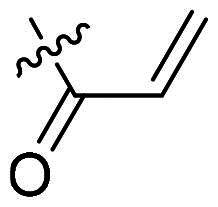	>1000	54.9 ± 25.7	>1000
PLW7	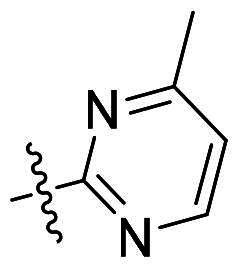	H	F	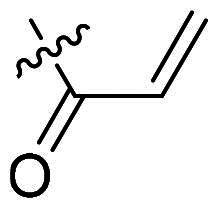	>1000	92.9 ± 13.8	>1000
PLW8	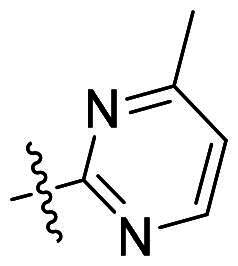	H	H	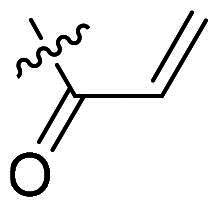	>1000	98.0 ± 4.0	>1000
PLW9	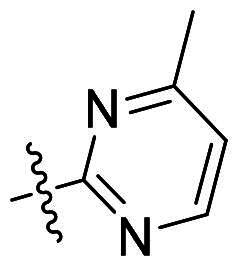	Me	H	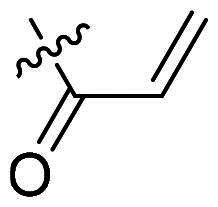	>1000	110.6 ± 2.5	>1000
PLW10	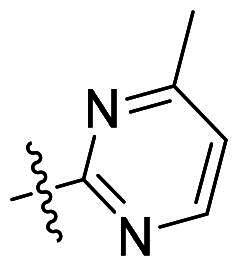	F	H	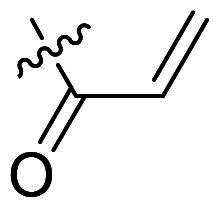	174.7 ± 10.9	7.7 ± 2.9	>1000
PLW11	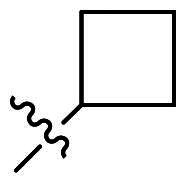	F	H	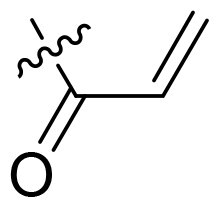	27.7 ± 4.2	1.2 ± 0.3	>1000
PLW12	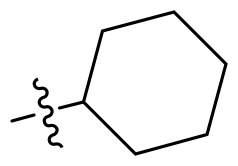	F	H	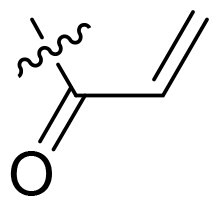	>1000	302.6 ± 5.2	>1000
PLW13	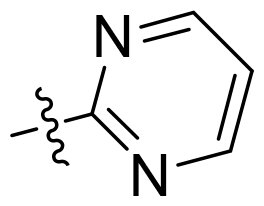	F	H	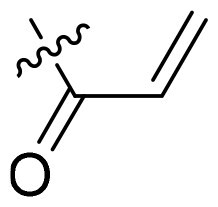	>1000	110.4 ± 5.5	>1000
PLW14 (PLW559)	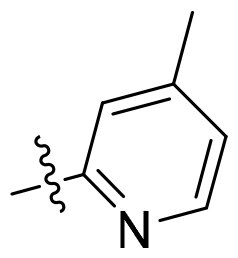	F	H	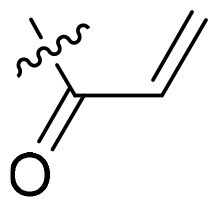	>1000	11.8 ± 0.1	>1000

^a^ The data are mean values from at least two independent experiments.

### 2.2. Chemical synthesis

**Scheme 1.**
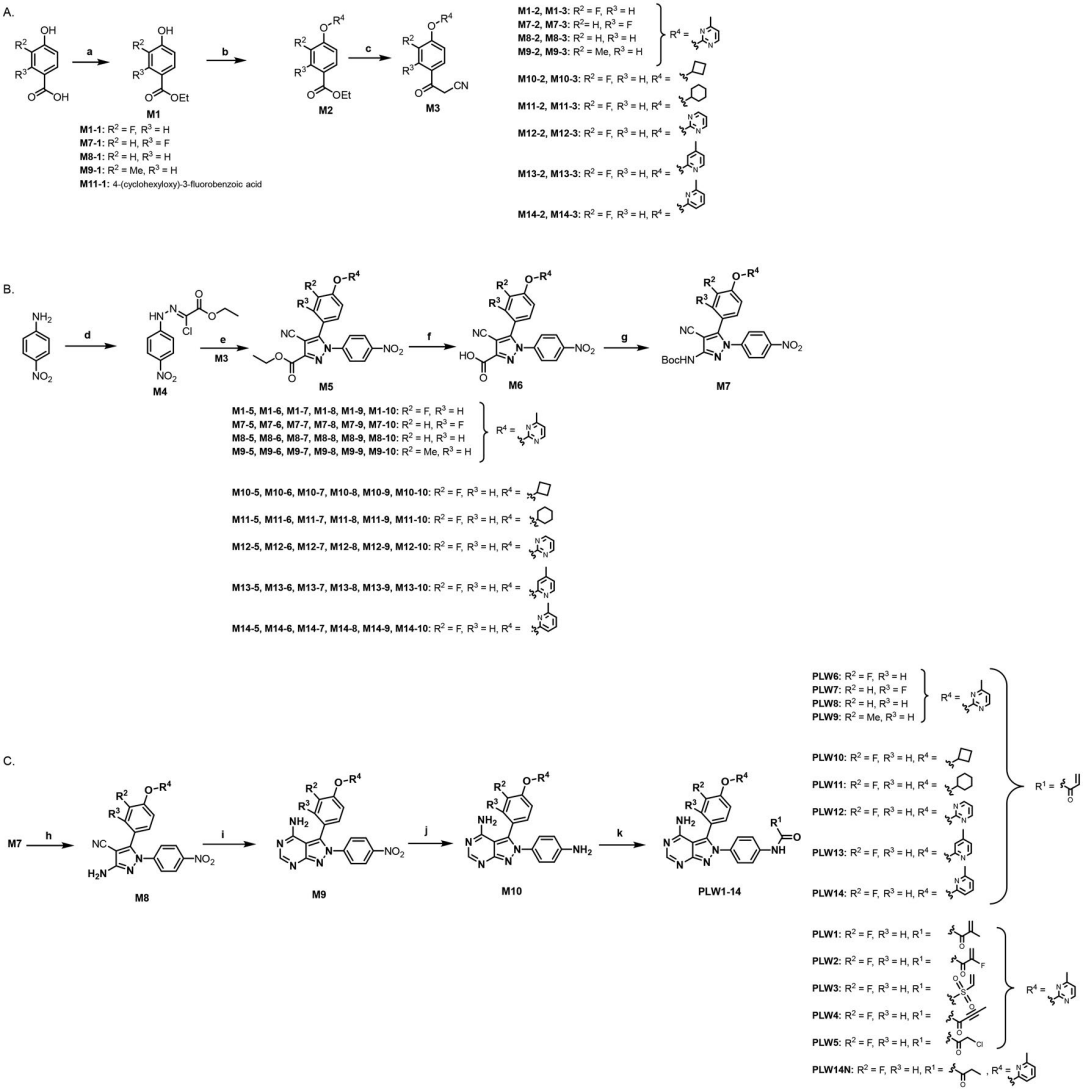
Synthesis of compounds PLW1-14.

As shown in Scheme 1, compounds **PLW1–14** were synthesised through a stepwise and modular synthetic route. The synthesis was initiated by esterification of substituted benzoic acids under thionyl chloride/ethanol conditions to afford the corresponding ethyl ester intermediates **M1**. Introduction of the *R*^4^ substituents was achieved *via* a copper-catalyzed Ullmann-type *O*-coupling reaction, yielding intermediates **M2**, which were subsequently treated with acetonitrile and n-butyllithium to generate the key *β*-ketonitrile intermediates **M3**. Construction of the pyrazole core was accomplished through a Japp–Klingemann reaction. Specifically, substituted nitroanilines (**M4**) were converted into the corresponding diazonium salts and coupled with **M3** to afford the cyclized intermediates **M5**. Subsequent hydrolysis of the ester functionality in **M5** using lithium hydroxide provided the carboxylic acid intermediates **M6**, which underwent a Curtius rearrangement in the presence of diphenylphosphoryl azide (DPPA) and *tert*-butanol to yield the Boc-protected amine intermediates **M7**. Removal of the Boc protecting group under trifluoroacetic acid (TFA) conditions furnished intermediates **M8**. Thereafter, intermediates **M8** were subjected to cyclocondensation with formamide at elevated temperature to construct the pyrazolo[3,4-*d*]pyrimidine scaffold (**M9**). Subsequent Pd/C-catalyzed hydrogenation reduced the nitro group to give the corresponding primary amine **M10**. Finally, acylation of **M10** with various propionyl chlorides or substituted acyl chlorides afforded the target compounds **PLW1–14**.

Reagents and conditions: (**a**) SOCl_2_, CH_3_CH_2_OH, 80 °C, 1 h, 92%; (**b**) K_3_PO_4_, CuI, ligand (*N*^1^-([1,1′-biphenyl]-2-yl)-*N*^2^-benzyloxalamide), 2-chloro-4-methylpyrimidine, DMSO, 130 °C, 24h, 81%; (**c**) n-BuLi, CH_3_CN, THF, −78 °C, 67%; (**d**) NaNO_2_, HCl/H_2_O, CH_3_COONa, ethyl 2-chloro-3-oxobutanoate, EtOH, 0 °C-rt, 44.5%; (**e**) Et_3_N, DCM, rt, 47.8%; (**f**) LiOH•H_2_O, THF/H_2_O, 88%; (**g**) DPPA, Et_3_N, 1,4-dioxane/*t*-BuOH, 65.7%; (**h**) TFA, DCM, rt, 73.5%; (**i**) formamide, 170 °C, 35%; (**j**) Pd/C, H_2_, MeOH, rt, 8 h, 80%; (**k**) acryloyl chloride, Py/DMF, 0 °C, 18%.

### 2.3. PLW559 covalently binds to FGFR2 kinase

**Figure 3.**
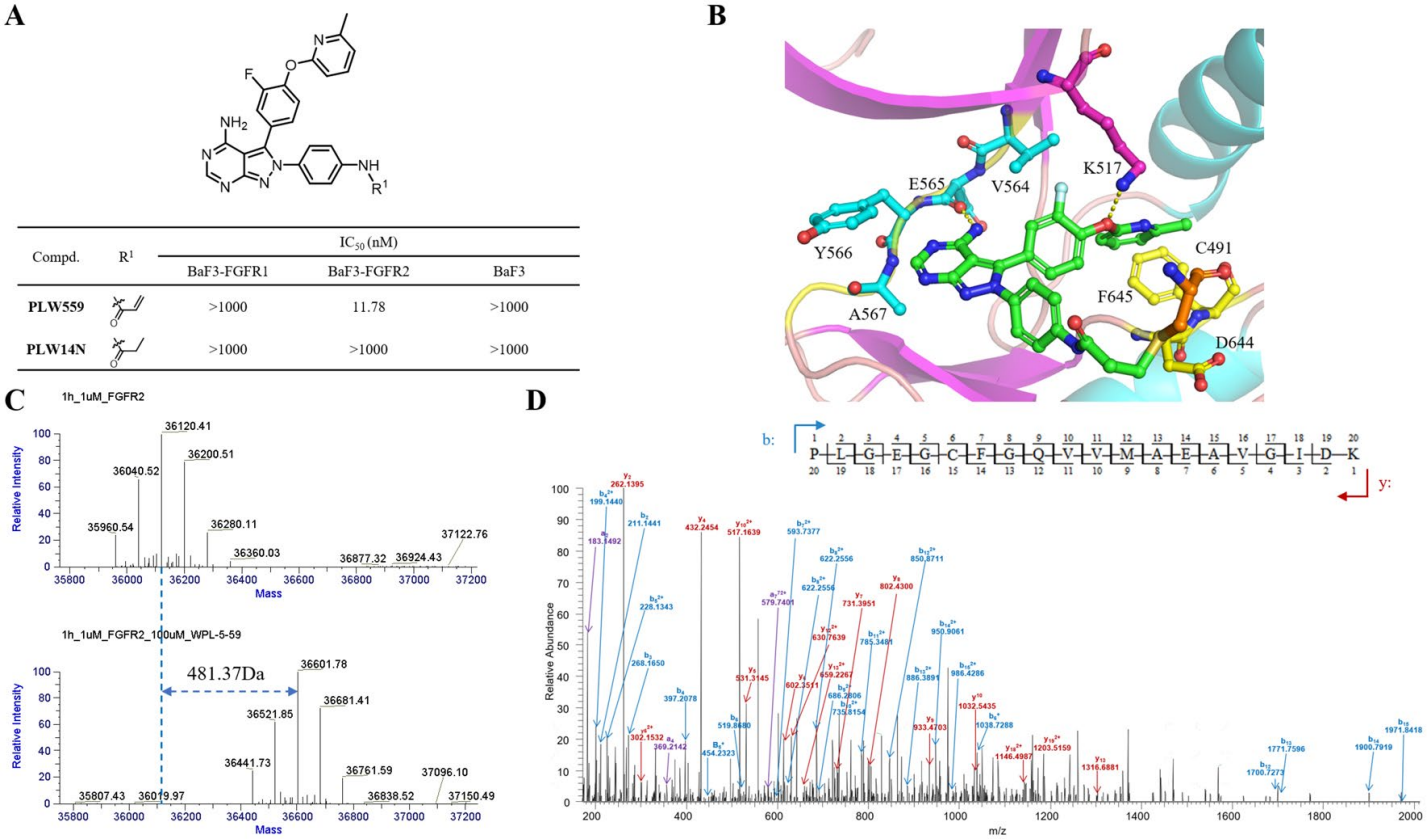
**PLW559** covalently binds to FGFR2 kinase. (A) Antiproliferative effects of **PLW559** and **PLW14N** against BaF3-FGFR1, BaF3-FGFR2 and parental BaF3 cell lines; (B) Docking results of **PLW559** with FGFR2; (C) Deconvoluted intact mass spectra of unmodified FGFR2 (top) and **PLW559**-labeled FGFR2 (bottom), acquired with 1 μg of protein; (D) Higher energy collision-induced dissociation (HCD) MS/MS spectrum of the [M + 2H]^2+^ ion at *m/z* 1251.581, derived from the human FGFR2 peptide PLGEGCFGQVVMAEAVGIDK containing one modified site. Predicted b- and y-type ions (partial list) are indicated above and below the peptide sequence, respectively.

To confirm the covalent binding mechanism of **PLW559** to FGFR2, the negative control compound **PLW14N** was designed and synthesised. **PLW14N** is structurally identical to **PLW559** but lacks the acrylamide moiety, thereby preventing it from undergoing Michael addition (Figure 3A). As designed, **PLW14N** showed negligible antiproliferative activity against BaF3-FGFR2 cells (IC_50_ > 1000 nM), confirming covalent binding is essential for FGFR2 inhibition. Subsequent molecular docking studies revealed the binding mode of **PLW559**. As shown in Figure 3B, **PLW559** adopts a pose similar to that of compound **2**, with its acrylamide warhead positioned to form a covalent bond with Cys491. The covalent interaction was further verified by mass spectrometry. Incubation of FGFR2 with **PLW559** resulted in a protein mass shift of approximately 481.37 Da, corresponding to the formation of a covalent FGFR2–**PLW559** adduct (Figure 3C). Moreover, higher-energy collision-induced dissociation (HCD) MS/MS analysis definitely identified Cys491 as the site of covalent modification (Figure 3D).

### 2.4. PLW559 selectively inhibits FGFR2 kinase activity

**Figure 4.**
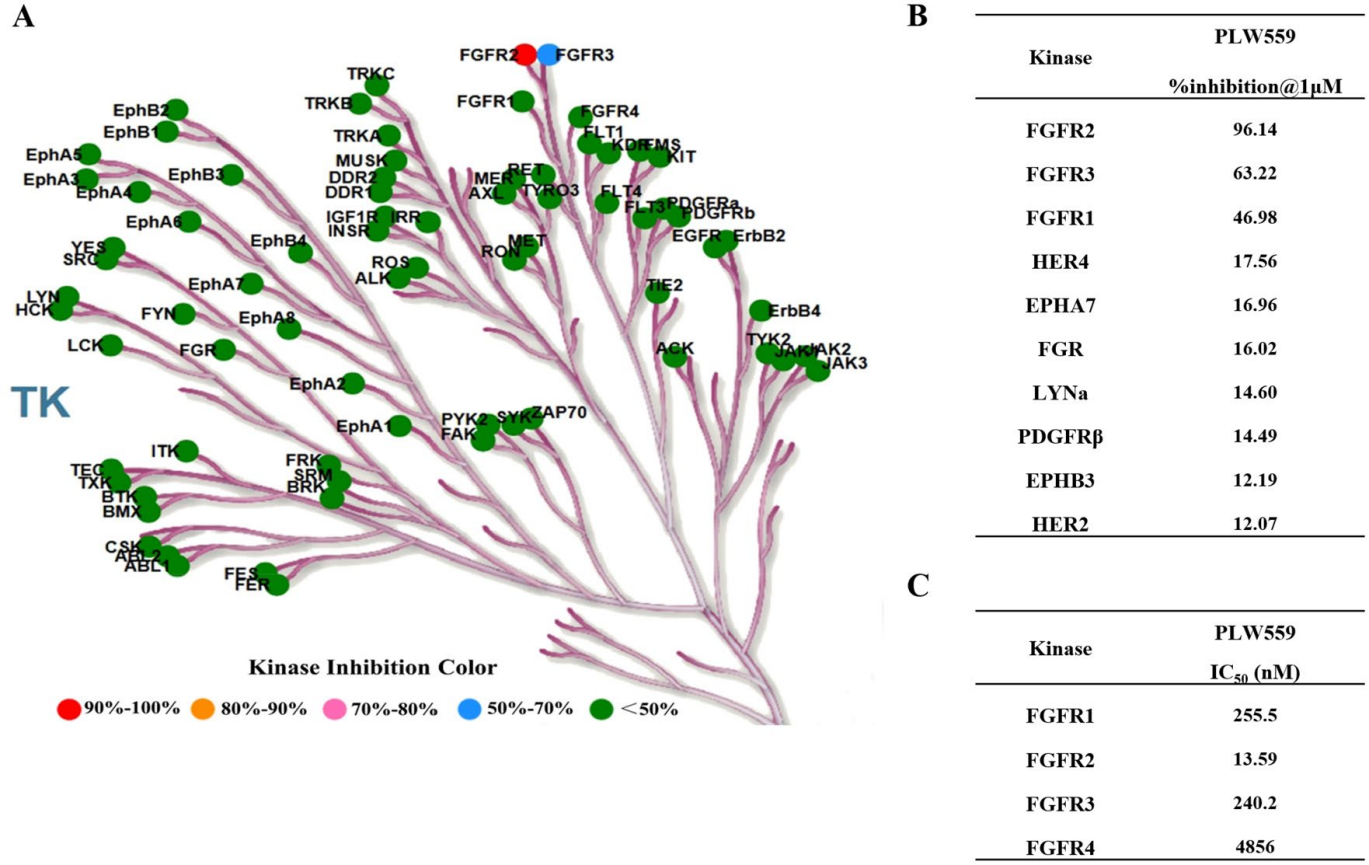
**PLW559** selectively inhibits FGFR2 kinase activity. (A) Selectivity profile of **PLW559** against a panel of 76 tyrosine kinases, presented as a heat map; (B) Kinases exhibiting the highest inhibition rates by **PLW559** at 1 μM; (C) IC_50_ values of **PLW559** against FGFR1-4. Pan-FGFR inhibitor TAS120 was included as a positive control and its IC_50_ values against FGFR1-4 were 0.793, 0.5634, 0.8203, and 2.724 nM, respectively.

The kinase selectivity profile of **PLW559** was evaluated against a panel of 76 human tyrosine kinases. The results, visualised in a kinome tree format (Figure 4A; Supporting Information Table S1), demonstrated that at a screening concentration of 1 μM, **PLW559** potently inhibited FGFR2 with an inhibition rate of 96.14%. Notably, with the exception of FGFR3, which showed an inhibition rate of 63.22%, all other kinases exhibited inhibition rates below 50% (Figure 4B). To quantify its enzymatic potency, mobility shift-based kinase assays were performed against FGFR1–4. As shown in Figure 4C, **PLW559** potently inhibited FGFR2 with an IC_50_ value of 13.59 nM. In contrast, it showed substantially weaker activity against FGFR1 (IC_50_ = 255.5 nM), FGFR3 (IC_50_ = 240.2 nM), and FGFR4 (IC_50_ = 4856 nM). Together, these results establish **PLW559** as a highly selective FGFR2 inhibitor.

### 2.5. PLW559 suppresses FGFR2 signaling and selectively Inhibits FGFR2-driven cancer Cell proliferation

**Figure 5.**
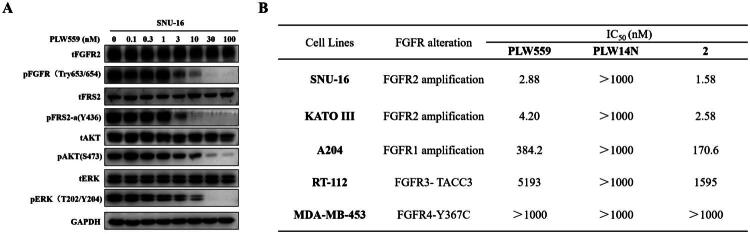
**PLW559** suppresses FGFR2 signalling and selectively inhibits FGFR2-driven cancer cell proliferation. (A) Inhibition of FGFR2-mediated signalling in SNU-16 cells. SNU-16 cells were treated with **PLW559** for 1 h before lysis and subsequent Western-blot analysis. GAPDH served as the loading control; (B) Antiproliferative activity of **PLW559** and **PLW14N** against cancer cell lines. Cell proliferation was assessed using the CCK-8 assay after 72 h of treatment with compound or 0.2% DMSO.

Pharmacological evaluation demonstrated that **PLW559** effectively blocked the FGFR2 signalling cascade in FGFR2-amplified SNU-16 cells. As shown in Figure 5A, treatment with **PLW559** led to a dose-dependent reduction in the phosphorylation of FGFR2, accompanied by reduced phosphorylation of its downstream effectors FRS2α, Akt, and ERK1/2, without altering total protein levels. **PLW559** also exhibited exceptional potency against FGFR2-amplified SNU-16 and KATO III cells, with IC_50_ values of 2.88 nM and 4.20 nM, respectively (Figure 5B). Notably, **PLW559** showed high selectivity for FGFR2 over other isoforms, displaying over 100-fold to 3000-fold lower potency in cell lines driven by FGFR1, FGFR3, or FGFR4. These results highlight the potential of **PLW559** as a highly specific lead candidate for treating FGFR2-dependent cancers.

### 2.6. PLW559 selectively induces apoptosis of FGFR2-overexpressed cancer cells

**Figure 6.**
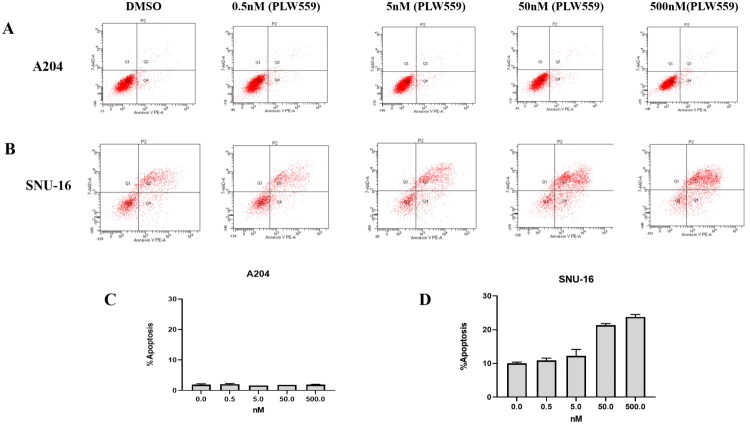
**PLW559** selectively induces apoptosis of FGFR2-overexpressed cancer cells. Apoptosis was analysed in A204 (A) and SNU-16 (B) cells following treatment with the indicated concentrations of **PLW559** for 48 h. Cells were stained with Annexin V and 7-AAD and analysed by flow cytometry; representative flow cytometry plots were shown. Quantitative summaries of the flow cytometry results are presented in bar charts (C and D).

The pro-apoptotic effect of **PLW559** was evaluated using flow cytometry. As shown in Figure 6A and 6C, **PLW559** induced minimal apoptosis in A204 cells across a concentration range of 0.5–500 nM. In contrast, **PLW559** triggered dose-dependent apoptosis in FGFR2-amplified SNU-16 cells (Figure 6B and 6D), with a pronounced increase in the apoptotic populations at concentrations of 50 nM and 500 nM. These results demonstrate that **PLW559** selectively induces apoptosis in FGFR2-driven cancer cells.

## 3. Conclusion

Overall, using a scaffold-hopping strategy, we successfully developed a novel series of irreversible and selective FGFR2 inhibitors based on a 2*H*-pyrazolo[3,4-*d*]pyrimidin-4-amine scaffold. From this series, **PLW559** emerged as a representative lead compound, exhibiting potent inhibition of FGFR2 with an IC_50_ value of 13.59 nM. In a broad selectivity screen against 76 receptor tyrosine kinases at 1 μM, **PLW559** demonstrated excellent selectivity. At the cellular level, **PLW559** potently suppressed the proliferation of BaF3-FGFR2 cells and FGFR2-driven cancer cell lines (SNU-16 and KATO III), while showing markedly reduced activity in non-FGFR2-dependent models. Furthermore, **PLW559** effectively blocked FGFR2 downstream signalling and selectively induced apoptosis in FGFR2-amplified cancer cells. However, although **PLW559** displayed comparable activity and selectivity to compound **2** *in vitro*, the *in vivo* PK properties and efficacy warrant further investigation. In summary, this study provides a promising lead compound with a novel scaffold for the future development of selective FGFR2-targeted therapies.

## 4. Experimental section

### 4.1. General methods for chemistry

Commercial reagents were used as received unless otherwise noted. All solvents were of reagent grade and, with the exception of anhydrous tetrahydrofuran, were used without prior drying. Reaction progress was monitored by thin-layer chromatography (TLC) on silica gel plates, visualised under UV light (254 nm) or by staining with phosphomolybdic acid. Purification was performed by flash chromatography on silica gel (100–200 mesh). ^1^H and 1³C NMR spectra were recorded on Bruker AV-400, Bruker Avance Neo 500, Agilent 500, or Bruker 600 spectrometers. Chemical shifts (δ) are reported in ppm relative to residual solvent signals (CDCl_3_: 7.26 ppm; DMSO-d_6_: 2.50 ppm) or tetramethylsilane (TMS = 0 ppm). Multiplicities are abbreviated as s (singlet), d (doublet), t (triplet), q (quartet), m (multiplet), with additional combinations (e.g., dd, dt) as observed. Low-resolution mass spectrometry (LRMS) was performed on a Shimadzu LCMS-2020 instrument using electrospray ionisation (ESI). High-resolution mass spectrometry (HRMS) was conducted on a Bruker MaXis 4 G time-of-flight (TOF) mass spectrometer with ESI. The purity of all final target compounds was determined to be >95% by analytical HPLC on an Agilent 1200 system equipped with a binary pump, a VWD detector, and a Dikma Diamonsil Plus C18 column (5 µm, 250 × 4.6 mm).

#### 4.1.1. Ethyl 3-fluoro-4-hydroxybenzoate (M1-1)

3-fluoro-4-hydroxybenzoic acid (10 g, 64.06 mmol) and ethanol (150 ml)were charged into a round-bottom flask with a magnetic stir bar. SOCl_2_ (30 ml) was slowly dropped into the above solution. After the dropping was completed, the reaction mixture was stirred for 1 h at 80 °C. After the completion, the reaction mixture was cooled to room temperature, and the ethanol was concentrated *in vacuo*. The resulting residue was purified by silica gel chromatography using PE/EA (v/v = 2:1) as eluent to obtained white solid **M1-1** (10.90 g, 92%).^1^H NMR (500 MHz, CDCl_3_) δ 7.80–7.74 (m, 2H), 7.04 (t, *J* = 8.6 Hz, 1H), 6.10 (s, 1H), 4.36 (q, *J* = 7.1 Hz, 2H), 1.38 (t, *J* = 7.1 Hz, 3H). LRMS (ESI) *m*/*z*: 183.10 [M–H]^–^.

*4.1.2. Ethyl 3-fluoro-4-((4-methylpyrimidin-2-yl)oxy)benzoate (****M1-2****)***M1-1** (10.90 g, 59.18 mmol), 2-chloro-4-methylpyrimidine (8.37 g, 65.01 mmol), K_3_PO_4_ (37.69 g, 177.54 mmol), CuI (1.13 g, 5.92 mmol) and **Ligand** (*N*^1^-([1,1′-biphenyl]-2-yl)-*N*^2^-benzyloxalamide, 2.04 g, 5.92 mmol) were charged into a thick wall solvent storage bottle with a magnetic stir bar. The reaction vial was evacuated and purged with argon three times, and DMSO (80 ml) was then added. The reaction mixture was stirred for 24 h at 130 °C. After the reaction was complete, it was cooled to room temperature, then diluted with 500 ml of water, and was extracted with EA (300 ml) three times. The organic layer was washed with brines, dried over anhydrous Na_2_SO_4_, filtered, and concentrated *in vacuo*. Subsequently, the resulting residue was purified by silica gel chromatography using PE/EA(v/v = 8:1) as eluent to obtain white solid **M1-2** (13.24 g, 81%). ^1^H NMR (500 MHz, CDCl_3_) δ 8.34 (d, *J* = 5.0 Hz, 1H), 7.92–7.83 (m, 2H), 7.34–7.30 (m, 1H), 6.94 (d, *J* = 5.0 Hz, 1H), 4.38 (q, *J* = 7.1 Hz, 2H), 2.48 (s, 3H), 1.38 (t, *J* = 7.1 Hz, 3H). LRMS (ESI) *m/z*: 277.10 [M + H]^+^.

#### 4.1.3. Methyl 2-([1,1’-biphenyl]-2-ylamino)-2-oxoacetate (LM)

*O*-aminobiphenyl (20 g, 118.19 mmol) was dissolved in THF (200 ml), and then triethylamine (21.4 ml, 153.65 mmol) was added. Oxalyl chloride monomethyl ester (17.38 g, 141.83 mmol) was slowly dropped into the above-mentioned mixed solution under an ice bath. The reaction was carried out at room temperature for 2 h. After the reaction was completed, THF was concentrated *in vacuo*. Water was added and pulped overnight, then water was filtered to obtain white solid **LM** (28.60 g, 94.8%).^1^H NMR (500 MHz, CDCl_3_) δ 8.96 (s, 1H), 8.34 (d, *J* = 8.2 Hz, 1H), 7.40 (t, *J* = 7.4 Hz, 2H), 7.36–7.25 (m, 4H), 7.20 (dd, *J* = 7.6, 1.5 Hz, 1H), 7.16–7.11 (m, 1H), 3.76 (s, 3H). LRMS (ESI) *m/z*: 256.10 [M + H]^+^.

#### 4.1.4. N^1^-([1,1’-biphenyl]-2-yl)-N^2^-benzyloxalamide (Ligand)

**LM** (28.60 g, 112.04 mmol), benzylamine (14.41 g, 134.45 mmol) and THF (200 ml) were added in a round-bottom flask with a magnetic stir bar. The reaction was stirred for 12 h at 80 °C. After the reaction was completed, it was cooled to room temperature. An appropriate amount of water was added to make a slurry overnight. The water was filtered to obtain white solid **Ligand** (36.21 g, 97.8%).^1^H NMR (500 MHz, CDCl_3_) δ 9.55 (s, 1H), 8.44 (dd, *J* = 8.3, 0.8 Hz, 1H), 7.83 (s, 1H), 7.53 (t, *J* = 7.5 Hz, 2H), 7.47–7.39 (m, 4H), 7.37–7.23 (m, 8H), 4.48 (d, *J* = 6.2 Hz, 2H). LRMS (ESI) *m/z:* 331.20 [M + H]^+^.

#### 4.1.5. 3-(3-fluoro-4-((4-methylpyrimidin-2-yl)oxy)phenyl)-3-oxopropanenitrile (M1-3)

The hexane solution of n-BuLi (2.5 M in Hexane, 25 ml, 62.3 mmol) was added to THF (60 ml). Under argon protection, acetonitrile (5 ml in 30 ml THF, 95.86 mmol) was slowly dropped into the above solution. After the addition was completed, the argon protection was removed. After the reaction for 1 h, **M1-2** (13.24 g in 30 ml THF, 47.93 mmol) was added dropwise, and the reaction mixture was stirred for 30 min, then raised to −45 °C and reacted for 2 h. After the reaction was completed, 1.0 M dilute hydrochloric acid solution was added dropwise to the reaction solution to acidify until neutral. The mixture was extracted three times with EA (200 ml), and the organic layer was washed with brines, dried over anhydrous Na_2_SO_4_, filtered, and concentrated *in vacuo*. Subsequently, the resulting residue was purified by silica gel chromatography using PE/EA (v/v = 1:1) as eluent to obtain white solid **M1-3** (8.71 g, 67%). ^1^H NMR (500 MHz, CDCl_3_) δ 8.36 (d, *J* = 5.0 Hz, 1H), 7.80–7.74 (m, 2H), 7.43 (dd, *J* = 8.5, 7.5 Hz, 1H), 6.98 (d, *J* = 5.0 Hz, 1H), 4.09 (s, 2H), 2.49 (s, 3H). LRMS (ESI) *m/z:* 272.15 [M + H]^+^.

#### 4.1.6. Ethyl (Z)-2-chloro-2-(2-(4-nitrophenyl)hydrazineylidene)acetate (M1-4)

p-Nitroaniline (20 g, 144.80 mmol) was dissolved in a mixed solution of HCl/H_2_O (80 ml, 1:5). An aqueous solution of NaNO_2_ (1.2 M, 130 ml) was slowly added under an ice bath, and the mixture was stirred for 30 min to obtain the diazonium salt intermediate. CH_3_COONa (13.07 g, 159.28 mmol) was added, followed by the slow addition of ethyl 2-chloroacetoacetate (26.22 g, 159.28 mmol). The reaction was then allowed to proceed at room temperature. After completion of the reaction, a small amount of water was added, and the mixture was extracted three times with ethyl acetate. The organic layer was dried over anhydrous Na_2_SO_4_, filtered, and concentrated. Subsequently, the resulting residue was purified by silica gel chromatography using PE/EA (v/v = 6:1) as eluent to afforded yellow solid **M1-4** (17.5 g, 44.5%). ^1^H NMR (500 MHz, DMSO-*d_6_*) δ 11.13 (s, 1H), 8.22 (d, *J* = 9.2 Hz, 2H), 7.49 (d, *J* = 9.3 Hz, 2H), 4.31 (q, *J* = 7.1 Hz, 2H), 1.30 (t, *J* = 7.1 Hz, 3H). LRMS (ESI) *m/z:* 270.05 [M - H]^-^.

#### 4.1.7. Ethyl 4-cyano-5-(3-fluoro-4-((4-methylpyrimidin-2-yl)oxy)phenyl)-1-(4-nitrophenyl)-1H-pyrazole-3-carboxylate (M1-5)

**M1-3** (8.71 g, 32.11 mmol) was dissolved in DCM (100 ml), followed by the addition of triethylamine (17.85 ml, 128.44 mmol). After stirring for 10 min, compound **M1-4** (10.47 g, 38.54 mmol) was added, and the reaction was allowed to proceed at room temperature until the starting material was consumed. Upon completion, the solvent was removed under reduced pressure. Water was added, and the mixture was extracted three times with ethyl acetate. The combined organic layers were dried, filtered, and concentrated. Subsequently, the resulting residue was purified by silica gel chromatography using PE/EA (v/v = 6:1) as eluent to afforded yellow solid **M1-5** (7.49 g, 47.8%). ^1^H NMR (500 MHz, CDCl_3_) δ 8.37 (d, *J* = 5.0 Hz, 1H), 8.30 (d, *J* = 9.0 Hz, 2H), 7.57 (d, *J* = 9.0 Hz, 2H), 7.39 (t, *J* = 8.0 Hz, 1H), 7.17 (dd, *J* = 13.8, 5.0 Hz, 2H), 6.97 (d, *J* = 5.0 Hz, 1H), 4.54 (q, *J* = 7.1 Hz, 2H), 2.50 (s, 3H), 1.48 (t, *J* = 7.1 Hz, 3H). LRMS (ESI) *m/z:* 489.20 [M + H]^+^.

#### 4.1.8. 4-cyano-5-(3-fluoro-4-((4-methylpyrimidin-2-yl)oxy)phenyl)-1-(4-nitrophenyl)-1H-pyrazole-3-carboxylic acid (M1-6)

**M1-5** (7.49 g, 15.33 mmol) was dissolved in THF/H_2_O (3:1, 160 ml), followed by the addition of LiOH·H_2_O (1.93 g, 45.99 mmol). The reaction was stirred at room temperature until completion. After the reaction, the mixture was neutralised with 1 M dilute hydrochloric acid (HCl) and extracted three times with DCM. The combined organic layers were dried over anhydrous Na_2_SO_4_, filtered, and concentrated under reduced pressure. Subsequently, the resulting residue was purified by silica gel chromatography using DCM/MeOH (v/v = 10:1) as eluent to afforded yellow solid **M1-6** (6.21 g, 88%). ^1^H NMR (500 MHz, DMSO-*d_6_*) δ 8.49 (d, *J* = 5.0 Hz, 1H), 8.35 (d, *J* = 9.0 Hz, 2H), 7.68 (d, *J* = 9.0 Hz, 2H), 7.56 (dd, *J* = 9.5, 6.9 Hz, 2H), 7.37–7.34 (m, 1H), 7.22 (d, *J* = 5.0 Hz, 1H), 2.43 (s, 3H). LRMS (ESI) *m/z:* 461.20 [M + H]^+^.

#### 4.1.9. Tert-butyl(4-cyano-5-(3-fluoro-4-((4-methylpyrimidin-2-yl)oxy)phenyl)-1-(4-nitrophenyl)-1H-pyrazol-3-yl)carbamate (M1-7)

**M1-6** (6.21 g, 13.49 mmol) was dissolved in 1,4-dioxane (30 ml) under an argon atmosphere. DPPA (6.31 g, 22.94 mmol), triethylamine (4.37 g, 43.17 mmol), and *tert*-butanol (20 ml) were sequentially added. The reaction was stirred at 110 °C for 1 h. After completion, the mixture was diluted with water and extracted three times with ethyl acetate. The combined organic layers were washed with brine, dried over anhydrous sodium sulphate, filtered, and concentrated under reduced pressure. Subsequently, the resulting residue was purified by silica gel chromatography using PE/EA (v/v = 1:2) as eluent to afforded yellow solid **M1-7** (4.71 g, 65.7%). ^1^H NMR (500 MHz, CDCl_3_) δ 8.38 (d, *J* = 4.9 Hz, 1H), 8.25 (d, *J* = 9.0 Hz, 2H), 7.48 (d, *J* = 9.0 Hz, 2H), 7.37 (t, *J* = 7.9 Hz, 1H), 7.20–7.14 (m, 2H), 6.97 (d, *J* = 4.9 Hz, 1H), 6.91 (s, 1H), 2.51 (s, 3H), 1.57 (s, 9H). LRMS (ESI) *m/z:* 532.20 [M + H]^+^.

#### 4.1.10. 3-amino-5-(3-fluoro-4-((4-methylpyrimidin-2-yl)oxy)phenyl)-1-(4-nitrophenyl)-1H-pyrazole-4-carbonitrile (M1-8)

**M1-7** (4.71 g, 8.86 mmol) was dissolved in DCM (40 ml), and trifluoroacetic acid (6 ml) was slowly added dropwise at 0 °C. After the addition was complete, the reaction was warmed to room temperature and stirred for 30 min. The mixture was then neutralised to pH 7 by dropwise addition of saturated aqueous NaHCO_3_. The solution was extracted three times with DCM, and the combined organic layers were dried over anhydrous Na_2_SO_4_, filtered, and concentrated under reduced pressure. Subsequently, the resulting residue was purified by silica gel chromatography using PE/EA (v/v = 1:2) as eluent to yielded yellow solid **M1-8** (2.8 g, 73.5%). ^1^H NMR (500 MHz, DMSO-*d_6_*) δ 8.50 (d, *J* = 5.0 Hz, 1H), 8.25 (d, *J* = 9.1 Hz, 2H), 7.55–7.50 (m, 2H), 7.48 (d, *J* = 9.1 Hz, 2H), 7.29 (dd, *J* = 8.3, 1.7 Hz, 1H), 7.22 (d, *J* = 5.0 Hz, 1H), 6.22 (s, 2H), 2.44 (s, 3H). LRMS (ESI) *m/z:* 432.25 [M + H]^+^.

#### 4.1.11. 3-(3-fluoro-4-((4-methylpyrimidin-2-yl)oxy)phenyl)-2-(4-nitrophenyl)-2H-pyrazolo[3,4-d]pyrimidin-4-amine (M1-9)

**M1-8** (2.8 g, 6.49 mmol) was placed in a flask and dissolved in formamide (30 ml). The reaction mixture was heated at 170 °C for 2 h. After completion, water was added to the reaction mixture, which was then extracted three times with ethyl acetate. The combined organic layers were washed three times with water, followed by three washes with brine (NaCl aq.). The organic phase was dried over anhydrous Na_2_SO_4_, filtered, and concentrated under reduced pressure. Subsequently, the resulting residue was purified by silica gel chromatography using DCM/MeOH (v/v = 10:1) as eluent to yielded yellow solid **M1-9** (1.04 g, 35%). ^1^H NMR (500 MHz, DMSO-*d_6_*) δ 8.51 (d, *J* = 5.0 Hz, 1H), 8.31 (d, *J* = 9.4 Hz, 3H), 7.68 (d, *J* = 9.0 Hz, 2H), 7.63 (dd, *J* = 10.8, 1.9 Hz, 1H), 7.51 (t, *J* = 8.2 Hz, 1H), 7.37 (dd, *J* = 8.3, 1.5 Hz, 1H), 7.22 (d, *J* = 5.1 Hz, 1H), 2.42 (s, 3H). LRMS (ESI) *m/z:* 459.35 [M + H]^+^.

#### 4.1.12. 2-(4-aminophenyl)-3-(3-fluoro-4-((4-methylpyrimidin-2-yl)oxy)phenyl)-2H-pyrazolo[3,4-d]pyrimidin-4-amine (M1-10)

**M1-9** (1.04 g, 2.27 mmol) was dissolved in methanol (20 ml), followed by the addition of 10% Pd/C (∼55% water content, 1 g). The reaction was carried out under a hydrogen atmosphere at room temperature for 8 h. After completion, the mixture was filtered through Celite to afford white solid **M1-10** (778 mg, 80% yield). ^1^H NMR (500 MHz, DMSO-*d_6_*) δ 8.50 (d, *J* = 5.0 Hz, 1H), 8.26 (s, 1H), 7.49–7.45 (m, 2H), 7.27 (dd, *J* = 8.3, 1.5 Hz, 1H), 7.21 (d, *J* = 5.0 Hz, 1H), 7.02 (d, *J* = 8.7 Hz, 2H), 6.53 (d, *J* = 8.7 Hz, 2H), 5.50 (s, 2H), 2.43 (s, 3H). LRMS (ESI) *m/z:* 429.40 [M + H]^+^.

#### 4.1.13. N-(4-(4-amino-3-(3-fluoro-4-((4-methylpyrimidin-2-yl)oxy)phenyl)-2H-pyrazolo[3,4-d]pyrimidin-2-yl)phenyl)methacrylamide (PLW1)

**M1-10** (100 mg, 0.23 mmol) was dissolved in DMF (5 ml), followed by addition of pyridine (0.5 ml). A solution of acryloyl chloride (18.7 μL, 0.23 mmol) in DMF was added dropwise at 0 °C, and the reaction was then warmed to room temperature. After completion, the reaction mixture was poured into water (20 ml) and extracted with ethyl acetate (30 ml × 3). The combined organic layers were dried over anhydrous sodium sulphate, filtered, and concentrated under reduced pressure. The crude product was purified by silica gel chromatography using DCM/MeOH (v/v = 10:1) as eluent to afford white solid **PLW1** (19.4 mg, 17% yield). **^1^H NMR** (500 MHz, DMSO-*d_6_*) δ 9.99 (s, 1H), 8.50 (d, *J* = 5.0 Hz, 1H), 8.29 (s, 1H), 7.76 (d, *J* = 8.9 Hz, 2H), 7.52 (dd, *J* = 10.9, 1.9 Hz, 1H), 7.47 (t, *J* = 8.3 Hz, 1H), 7.35 (d, *J* = 8.9 Hz, 2H), 7.29 (dd, *J* = 8.3, 1.5 Hz, 1H), 7.21 (d, *J* = 5.0 Hz, 1H), 5.81 (s, 1H), 5.54 (s, 1H), 2.41 (s, 3H), 1.95 (s, 3H). **^13^C NMR** (126 MHz, DMSO-*d_6_*) δ 170.16, 166.45, 162.78, 158.86, 158.78, 158.42, 156.17, 153.23 (d, *J* = 248.6 Hz), 140.32 (d, *J* = 12.2 Hz), 139.61, 138.80, 134.40, 133.27, 126.70, 126.28 (d, *J* = 7.8 Hz), 125.92, 124.15, 119.70, 119.30, 118.25 (d, *J* = 19.9 Hz), 116.33, 100.55, 22.90, 18.03. ESI-HRMS *m/z:* calcd. For C_26_H_21_FN_8_O_2_ [M + H]^+^ 497.1844; found: 497.1844. HPLC: 98.5998%.

#### 4.1.14. N-(4-(4-amino-3-(3-fluoro-4-((4-methylpyrimidin-2-yl)oxy)phenyl)-2H-pyrazolo[3,4-d]pyrimidin-2-yl)phenyl)-2-fluoroacrylamide (PLW2)

**PLW2** (7.6 mg, 6.5%, white solid) was synthesised from **M1-10** (100 mg, 0.23 mmol) in a similar manner as **PLW1**. **^1^H NMR** (**500 MHz,** DMSO-*d_6_***)** δ 13.48 (s, 1H), 8.49 (d, *J* = 5.0 Hz, 1H), 8.33 (s, 1H), 7.60 (d, *J* = 11.0 Hz, 1H), 7.39 (t, *J* = 8.3 Hz, 1H), 7.25–7.19 (m, 2H), 7.06 (d, *J* = 8.7 Hz, 2H), 6.56 (d, *J* = 8.7 Hz, 2H), 5.41 (dd, *J* = 46.2, 1.9 Hz, 1H), 5.30 (dd, *J* = 13.9, 2.3 Hz, 1H), 2.42 (s, 3H). **^13^C NMR** (**126 MHz**, DMSO-*d_6_*) δ 171.28, 164.02, 159.86, 159.00 (d, *J* = 256.7 Hz), 157.42, 154.68, 154.56 (d, *J* = 20.9 Hz), 152.51, 150.05, 146.18, 140.82 (d, *J* = 12.1 Hz),139.71, 130.13, 128.51, 127.42, 127.17, 123.90, 120.51 (d, *J* = 19.6 Hz),117.41, 113.74, 102.05 (d, *J* = 15.9 Hz),104.78, 24.01. ESI-HRMS *m/z:* calcd. For C_25_H_18_F_2_N_8_O_2_ [M + H]^+^ 501.1594; found: 501.1583. HPLC: 97.9257%.

#### 4.1.15. N-(4-(4-amino-3-(3-fluoro-4-((4-methylpyrimidin-2-yl)oxy)phenyl)-2H-pyrazolo[3,4-d]pyrimidin-2-yl)phenyl)ethenesulfonamide (PLW3)

**PLW3** (11 mg, 9.2%, white solid) was synthesised from **M1-10** (100 mg, 0.23 mmol) in a similar manner as **PLW1**. **^1^H NMR** (500 MHz, DMSO-*d_6_*) δ 10.35 (s, 1H), 8.50 (d, *J* = 5.0 Hz, 1H), 8.29 (s, 1H), 7.51–7.49 (m, 1H), 7.47 (t, *J* = 4.9 Hz, 1H), 7.35 (d, *J* = 8.9 Hz, 2H), 7.28 (dd, *J* = 8.3, 1.7 Hz, 1H), 7.23 (d, *J* = 5.0 Hz, 1H), 7.18 (d, *J* = 8.9 Hz, 2H), 6.82 (dd, *J* = 16.4, 10.0 Hz, 1H), 6.13 (d, *J* = 16.4 Hz, 1H), 6.04 (d, *J* = 9.9 Hz, 1H), 2.43 (s, 3H). **^13^C NMR** (126 MHz, DMSO-*d_6_*) δ 173.23, 165.84, 161.89, 161.82, 161.44, 159.22, 156.29 (d, *J* = 249.4 Hz), 143.38 (d, *J* = 12.0 Hz), 140.68, 138.39, 137.56, 136.70, 132.07, 130.63, 129.73, 129.63, 127.22, 121.48, 121.28 (d, *J* = 19.2 Hz), 119.39, 103.59, 25.98. ESI-HRMS *m/z:* calcd. For C_24_H_19_FN_8_O_3_S [M + H]^+^ 519.1358; found: 519.1354. HPLC: 96.7220%.

#### 4.1.16. N-(4-(4-amino-3-(3-fluoro-4-((4-methylpyrimidin-2-yl)oxy)phenyl)-2H-pyrazolo[3,4-d]pyrimidin-2-yl)phenyl)but-2-ynamide (PLW4)

**PLW4**(17.6 mg, 15.5%, white solid) was synthesised from **M1-10** (100 mg, 0.23 mmol) in a similar manner as **PLW1**. **^1^H NMR** (**500 MHz**, DMSO-*d_6_*) δ 10.83 (s, 1H), 8.50 (d, *J* = 5.0 Hz, 1H), 8.29 (s, 1H), 7.65 (d, *J* = 8.9 Hz, 2H), 7.52 (dd, *J* = 10.9, 1.9 Hz, 1H), 7.47 (t, *J* = 8.3 Hz, 1H), 7.35 (d, *J* = 8.9 Hz, 2H), 7.29 (dd, *J* = 8.3, 1.4 Hz, 1H), 7.22 (d, *J* = 5.0 Hz, 1H), 2.42 (s, 3H), 2.06 (s, 3H). **^13^C NMR** (**126 MHz**, DMSO-*d_6_*) δ 173.22, 165.87, 161.89 (d, *J* = 7.4 Hz), 161.50, 159.29, 156.32 (d, *J* = 248.7 Hz), 153.07, 143.47 (d, *J* = 2.0 Hz), 143.38, 141.32, 137.50, 136.64, 132.09, 129.77, 129.18, 127.26, 121.80, 121.32 (d, *J* = 20.9 Hz). 119.40, 103.68, 87.27, 78.15, 25.98, 5.68. ESI-HRMS *m/z:* calcd. For C_26_H_20_FN_8_O_2_ [M + H]^+^ 495.1688; found: 495.1686. HPLC: 99.0122%.

#### 4.1.17. N-(4-(4-amino-3-(3-fluoro-4-((4-methylpyrimidin-2-yl)oxy)phenyl)-2H-pyrazolo[3,4-d]pyrimidin-2-yl)phenyl)-2-chloroacetamide (PLW5)

**PLW5** (12.3 mg, 10.6%, white solid) was synthesised from **M1-10** (100 mg, 0.23 mmol) in a similar manner as **PLW1**. **^1^H NMR** (**500 MHz**, DMSO-*d_6_*) δ 10.53 (s, 1H), 8.49 (d, *J* = 5.0 Hz, 1H), 8.28 (s, 1H), 7.66 (d, *J* = 8.9 Hz, 2H), 7.51 (dd, *J* = 10.9, 2.0 Hz, 1H), 7.47 (t, *J* = 8.3 Hz, 1H), 7.37 (d, *J* = 8.9 Hz, 2H), 7.28 (dd, *J* = 8.3, 1.4 Hz, 1H), 7.21 (d, *J* = 5.0 Hz, 1H), 4.26 (s, 2H), 2.41 (s, 3H). **^13^C NMR** (**126 MHz**, DMSO-*d_6_*) δ 173.21, 167.41, 165.85, 161.92, 161.84, 161.49, 159.26, 156.31 (d, *J* = 249.0 Hz), 143.40 (d, *J* = 11.8 Hz), 141.25, 137.52, 136.65, 132.08, 129.76, 129.31, 127.25, 121.67, 121.30 (d, *J* = 20.2 Hz), 119.39, 103.62, 45.97, 25.98. ESI-HRMS *m/z:* calcd. For C_24_H_19_ClFN_8_O_2_ [M + H]^+^ 505.1298; found: 505.1299. HPLC: 99.3611%.

#### 4.1.18. N-(4-(4-amino-3-(3-fluoro-4-((4-methylpyrimidin-2-yl)oxy)phenyl)-2H-pyrazolo[3,4-d]pyrimidin-2-yl)phenyl)acrylamide (PLW6)

**PLW6** (20 mg, 18%, white solid) was synthesised from **M1-10** (100 mg, 0.23 mmol) in a similar manner as **PLW1**. **^1^H NMR (500 MHz,** DMSO-*d_6_***)** δ 10.40 (s, 1H), 8.49 (d, *J* = 5.0 Hz, 1H), 8.28 (s, 1H), 7.73 (d, *J* = 8.8 Hz, 2H), 7.52 (dd, *J* = 10.9, 1.7 Hz, 1H), 7.47 (t, *J* = 8.3 Hz, 1H), 7.36 (d, *J* = 8.8 Hz, 2H), 7.29 (d, *J* = 8.3 Hz, 1H), 7.21 (d, *J* = 5.0 Hz, 1H), 6.43 (dd, *J* = 17.0, 10.1 Hz, 1H), 6.28 (dd, *J* = 17.0, 1.7 Hz, 1H), 5.79 (dd, *J* = 10.1, 1.7 Hz, 1H), 2.41 (s, 3H). **^13^C NMR (126 MHz,** DMSO-*d_6_***)** δ 171.28, 163.88, 159.95, 159.88, 158.40 (d, *J* = 279.3 Hz), 155.34, 153.36, 141.40 (d, *J* = 12.3 Hz), 139.83, 135.53, 134.40, 132.03, 127.99, 127.82 (d, *J* = 2.1 Hz), 127.38, 127.32, 127.28, 125.28, 119.69, 119.35 (d, *J* = 19.9 Hz), 117.44, 101.65, 24.01. ESI-HRMS *m/z:* calcd. For C_25_H_19_FN_8_O_2_ [M + H]^+^ 483.1688; found: 483.1685. HPLC: 97.9732%.

#### 4.1.19. Ethyl 2-fluoro-4-hydroxybenzoate (M7-1)

**M7-1** (11.3 g, 95.8%, white solid) was synthesised from 2-fluoro-4-hydroxybenzoic acid (10 g, 64.06 mmol) in a similar manner as **M1-1**. ^1^H NMR (500 MHz, CDCl_3_) δ 7.85 (t, *J* = 8.5 Hz, 1H), 6.66 (ddd, *J* = 14.5, 10.4, 2.4 Hz, 2H), 4.37 (q, *J* = 7.1 Hz, 2H), 1.39 (t, *J* = 7.1 Hz, 3H). LRMS (ESI) *m/z*: 185.25 [M + H]^+^.

#### 4.1.20. Ethyl 2-fluoro-4-((4-methylpyrimidin-2-yl)oxy)benzoate (M7-2)

**M7-2** (14.1 g, 83.2%, white solid) was synthesised from **M7-1** (11.3 g, 61.36 mmol) in a similar manner as **M1-2**. ^1^H NMR (500 MHz, CDCl_3_) δ 8.38 (d, *J* = 5.0 Hz, 1H), 7.99 (t, *J* = 8.5 Hz, 1H), 7.07–7.01 (m, 2H), 6.96 (d, *J* = 5.0 Hz, 1H), 4.38 (q, *J* = 7.1 Hz, 2H), 2.50 (s, 3H), 1.38 (t, *J* = 7.1 Hz, 3H). LRMS (ESI) *m/z*: 277.15 [M + H]^+^.

#### 4.1.21. 3-(2-fluoro-4-((4-methylpyrimidin-2-yl)oxy)phenyl)-3-oxopropanenitrile (M7-3)

**M7-3** (9.55 g, 69%, white solid) was synthesised from **M7-2** (14.1 g, 51.05 mmol) in a similar manner as **M1-3**. ^1^H NMR (500 MHz, CDCl_3_) δ 8.41 (d, *J* = 5.0 Hz, 1H), 8.04 (t, *J* = 8.6 Hz, 1H), 7.14 (ddd, *J* = 14.5, 10.5, 2.2 Hz, 2H), 7.01 (d, *J* = 5.0 Hz, 1H), 4.09 (d, *J* = 2.7 Hz, 2H), 2.52 (s, 3H). LRMS (ESI) *m/z*: 272.15 [M + H]^+^.

#### 4.1.22. Ethyl 4-cyano-5-(2-fluoro-4-((4-methylpyrimidin-2-yl)oxy)phenyl)-1-(4-nitrophenyl)-1H-pyrazole-3-carboxylate (M7-5)

**M7-5** (8.68 g, 50.5%, yellow solid) was synthesised from **M7-3** (9.55 g, 35.22 mmol) and **M1-4** (11.48 g, 42.26 mmol) in a similar manner as **M1-5**. ^1^H NMR (500 MHz, CDCl_3_) δ 8.27 (d, *J* = 9.0 Hz, 2H), 7.70 (d, *J* = 9.1 Hz, 2H), 7.56 (d, *J* = 9.0 Hz, 2H), 7.53 (d, *J* = 8.2 Hz, 1H), 7.24 (dd, *J* = 8.6, 2.1 Hz, 1H), 7.12 (dd, *J* = 12.3, 2.1 Hz, 1H), 4.55 (q, *J* = 7.1 Hz, 2H), 2.24 (s, 3H), 1.48 (t, *J* = 7.1 Hz, 3H). LRMS (ESI) *m/z*: 489.20 [M + H]^+^.

#### 4.1.23. 4-cyano-5-(2-fluoro-4-((4-methylpyrimidin-2-yl)oxy)phenyl)-1-(4-nitrophenyl)-1H-pyrazole-3-carboxylic acid (M7-6)

**M7-6** (6.52 g, 79.7%, yellow solid) was synthesised from **M7-5** (8.68 g, 17.78 mmol) in a similar manner as **M1-6**. ^1^H NMR (500 MHz, DMSO-*d_6_*) δ 8.50 (d, *J* = 5.0 Hz, 1H), 8.33 (d, *J* = 9.0 Hz, 2H), 7.68 (d, *J* = 9.0 Hz, 2H), 7.64 (d, *J* = 8.4 Hz, 1H), 7.40 (dd, *J* = 11.0, 2.2 Hz, 1H), 7.28 (dd, *J* = 8.5, 2.2 Hz, 1H), 7.21 (d, *J* = 5.0 Hz, 1H), 2.41 (s, 3H). LRMS (ESI) *m/z*: 461.15 [M + H]^+^.

#### 4.1.24. Tert-butyl(4-cyano-5-(2-fluoro-4-((4-methylpyrimidin-2-yl)oxy)phenyl)-1-(4-nitrophenyl)-1H-pyrazol-3-yl)carbamate (M7-7)

**M7-7** (5.09 g, 67.6%, yellow solid) was synthesised from **M7-6** (6.52 g, 14.17 mmol) in a similar manner as **M1-7**. ^1^H NMR (500 MHz, DMSO-*d_6_*) δ 10.22 (s, 1H), 8.50 (d, *J* = 5.0 Hz, 1H), 8.29 (d, *J* = 9.1 Hz, 2H), 7.63 (t, *J* = 8.4 Hz, 1H), 7.56 (d, *J* = 9.1 Hz, 2H), 7.40 (dd, *J* = 11.0, 2.2 Hz, 1H), 7.27 (dd, *J* = 8.5, 2.2 Hz, 1H), 7.21 (d, *J* = 5.0 Hz, 1H), 2.42 (s, 3H), 1.48 (s, 9H). LRMS (ESI) *m/z*: 532.25 [M + H]^+^.

#### 4.1.25. 3-amino-5-(2-fluoro-4-((4-methylpyrimidin-2-yl)oxy)phenyl)-1-(4-nitrophenyl)-1H-pyrazole-4-carbonitrile (M7-8)

**M7-8** (2.95 g, 71.4%, yellow solid) was synthesised from **M7-7** (5.09 g, 9.58 mmol) in a similar manner as **M1-8**. ^1^H NMR (500 MHz, DMSO-*d_6_*) δ 8.50 (d, *J* = 5.0 Hz, 1H), 8.23 (d, *J* = 9.1 Hz, 2H), 7.61 (t, *J* = 8.4 Hz, 1H), 7.47 (d, *J* = 9.1 Hz, 2H), 7.38 (dd, *J* = 11.0, 2.2 Hz, 1H), 7.26 (dd, *J* = 8.5, 2.2 Hz, 1H), 7.21 (d, *J* = 5.0 Hz, 1H), 2.42 (s, 3H). LRMS (ESI) *m/z*: 432.15 [M + H]^+^.

#### 4.1.26. 3-(2-fluoro-4-((4-methylpyrimidin-2-yl)oxy)phenyl)-2-(4-nitrophenyl)-2H-pyrazolo[3,4-d]pyrimidin-4-amine (M7-9)

**M7-9** (846 mg, 27%, yellow solid) was synthesised from **M7-8** (2.95 g, 6.84 mmol) in a similar manner as **M1-9**. ^1^H NMR (500 MHz, DMSO-*d_6_*) δ 8.51 (d, *J* = 5.0 Hz, 1H), 8.31 (s, 1H), 8.30 (d, *J* = 9.0 Hz, 2H), 7.71 (t, *J* = 8.4 Hz, 1H), 7.65 (d, *J* = 9.0 Hz, 2H), 7.31 (dd, *J* = 10.8, 2.2 Hz, 1H), 7.26 (dd, *J* = 8.4, 2.3 Hz, 1H), 7.21 (d, *J* = 5.0 Hz, 1H), 2.41 (s, 3H). LRMS (ESI) *m/z*: 459.15 [M + H]^+^.

#### 4.1.27. 2-(4-aminophenyl)-3-(2-fluoro-4-((4-methylpyrimidin-2-yl)oxy)phenyl)-2H-pyrazolo[3,4-d]pyrimidin-4-amine (M7-10)

**M7-10** (672 mg, 84.8%, yellow solid) was synthesised from **M7-9** (846 mg, 1.85 mmol) in a similar manner as **M1-10**. ^1^H NMR (500 MHz, DMSO-*d_6_*) δ 8.50 (d, *J* = 5.0 Hz, 1H), 8.24 (s, 1H), 7.57 (t, *J* = 8.4 Hz, 1H), 7.27 (dd, *J* = 10.6, 2.2 Hz, 1H), 7.22–7.17 (m, 2H), 6.97 (d, *J* = 8.7 Hz, 2H), 6.49 (d, *J* = 8.7 Hz, 2H), 2.42 (s, 3H). LRMS (ESI) *m/z*: 429.20 [M + H]^+^.

#### 4.1.28. N-(4-(4-amino-3-(2-fluoro-4-((4-methylpyrimidin-2-yl)oxy)phenyl)-2H-pyrazolo[3,4-d]pyrimidin-2-yl)phenyl)acrylamide (PLW7)

**PLW7** (46 mg, 13.9%, white solid) was synthesised from **M7-10** (300 mg, 0.7 mmol) in a similar manner as **PLW1**. **^1^H NMR** (500 MHz, DMSO-*d_6_*) δ 10.37 (s, 1H), 8.52 (d, *J* = 5.0 Hz, 1H), 8.30 (s, 1H), 7.73 (d, *J* = 8.9 Hz, 2H), 7.65 (t, *J* = 8.4 Hz, 1H), 7.34 (d, *J* = 8.9 Hz, 2H), 7.30 (dd, *J* = 10.7, 2.2 Hz, 1H), 7.23 (dd, *J* = 8.5, 2.2 Hz, 1H), 7.22 (d, *J* = 5.1 Hz, 1H), 6.43 (dd, *J* = 17.0, 10.1 Hz, 1H), 6.28 (dd, *J* = 17.0, 1.9 Hz, 1H), 5.79 (dd, *J* = 10.1, 1.9 Hz, 1H), 2.42 (s, 3H). **^13^C NMR** (126 MHz, DMSO-*d_6_*) δ 171.16, 164.08, 163.87, 159.94 (d, *J* = 249.1 Hz), 159.91, 159.88, 159.84, 157.32, 156.20 (d, *J* = 11.3 Hz), 139.87, 134.63, 133.28, 132.05, 130.49, 127.94, 126.35, 119.78, 118.92, 117.54, 113.25 (d, *J* = 15.7 Hz), 110.68 (d, *J* = 24.4 Hz)102.57, 24.03. ESI-HRMS *m/z:* calcd. For C_25_H_19_FN_8_O_2_ [M + H]^+^ 483.1688; found: 483.1685. HPLC: 95.4063%.

#### 4.1.29. Ethyl 4-hydroxybenzoate (M8-1)

**M8-1** (9.08 g, 75.5%, white solid) was synthesised from 4-hydroxybenzoic acid (10 g, 72.4 mmol) in a similar manner as **M1-1**. ^1^H NMR (500 MHz, CDCl_3_) δ 7.95 (d, *J* = 8.8 Hz, 2H), 6.89 (d, *J* = 8.8 Hz, 2H), 4.35 (q, *J* = 7.1 Hz, 2H), 1.38 (t, *J* = 7.1 Hz, 3H). LRMS (ESI) *m/z*: 167.15 [M + H]^+^.

#### 4.1.30. Ethyl 4-((4-methylpyrimidin-2-yl)oxy)benzoate (M8-2)

**M8-2** (12.08 g, 85.6%, white solid) was synthesised from **M8-1** (9.08 g, 54.66 mmol) in a similar manner as **M1-2**. ^1^H NMR (500 MHz, CDCl_3_) δ 8.37 (d, *J* = 5.0 Hz, 1H), 8.11 (d, *J* = 8.8 Hz, 2H), 7.26 (d, *J* = 8.8 Hz, 3H), 6.93 (d, *J* = 5.0 Hz, 1H), 4.37 (q, *J* = 7.1 Hz, 2H), 2.50 (s, 3H), 1.38 (t, *J* = 7.1 Hz, 3H). LRMS (ESI) *m/z*: 259.15 [M + H]^+^.

#### 4.1.31. 3-(4-((4-methylpyrimidin-2-yl)oxy)phenyl)-3-oxopropanenitrile (M8-3)

**M8-3** (8.71 g, 73.5%, white solid) was synthesised from **M8-2** (12.08 g, 46.78 mmol) in a similar manner as **M1-3**. ^1^H NMR (500 MHz, CDCl_3_) δ 8.38 (d, *J* = 5.0 Hz, 1H), 7.99 (d, *J* = 8.7 Hz, 2H), 7.35 (d, *J* = 8.7 Hz, 2H), 6.98 (d, *J* = 5.0 Hz, 1H), 4.09 (s, 2H), 2.51 (s, 3H). LRMS (ESI) *m/z*: 254.15 [M + H]^+^.

#### 4.1.32. ethyl4-cyano-5-(4-((4-methylpyrimidin-2-yl)oxy)phenyl)-1-(4-nitrophenyl)-1H-pyrazole-3-carboxylate (M8-5)

**M8-5** (9.43 g, 58.3%, yellow solid) was synthesised from **M8-3** (8.71 g, 34.38 mmol) and **M1-4** (11.3 g, 41.62 mmol) in a similar manner as **M1-5**. ^1^H NMR (500 MHz, CDCl_3_) δ 8.39 (d, *J* = 5.0 Hz, 1H), 8.28 (d, *J* = 9.0 Hz, 2H), 7.57 (d, *J* = 9.0 Hz, 2H), 7.39 (d, *J* = 8.7 Hz, 2H), 7.33 (d, *J* = 8.7 Hz, 2H), 6.97 (d, *J* = 5.0 Hz, 1H), 4.55 (q, *J* = 7.1 Hz, 2H), 2.52 (s, 3H), 1.48 (t, *J* = 7.1 Hz, 3H). LRMS (ESI) *m/z*: 471.20 [M + H]^+^.

#### 4.1.33. 4-cyano-5-(4-((4-methylpyrimidin-2-yl)oxy)phenyl)-1-(4-nitrophenyl)-1H-pyrazole-3-carboxylic acid (M8-6)

**M8-6** (6.88 g, 77.6%, yellow solid) was synthesised from **M8-5** (9.43 g, 20.04 mmol) in a similar manner as **M1-6**. ^1^H NMR (500 MHz, DMSO-*d_6_*) δ 8.46 (d, *J* = 5.0 Hz, 1H), 8.32 (d, *J* = 9.0 Hz, 2H), 7.66 (d, *J* = 9.1 Hz, 2H), 7.48 (d, *J* = 8.7 Hz, 2H), 7.33 (d, *J* = 8.7 Hz, 2H), 7.17 (d, *J* = 5.0 Hz, 1H), 2.40 (s, 3H). LRMS (ESI) *m/z*: 443.20 [M + H]^+^.*tert-butyl (4-cyano-5-(4-((4-methylpyrimidin-2-yl)oxy)phenyl)-1-(4-nitrophenyl)-1H-pyrazol-3-yl)carbamate (****M8-7****):* **M8-7** (5.5 g, 68.9%, yellow solid) was synthesised from **M8-6** (6.88 g, 15.55 mmol) in a similar manner as **M1-7**. ^1^H NMR (500 MHz, DMSO-*d_6_*) δ 10.14 (s, 1H), 8.47 (d, *J* = 5.0 Hz, 1H), 8.28 (d, *J* = 9.0 Hz, 2H), 7.54 (d, *J* = 9.0 Hz, 2H), 7.46 (d, *J* = 8.6 Hz, 2H), 7.32 (d, *J* = 8.6 Hz, 2H), 7.17 (d, *J* = 5.0 Hz, 1H), 2.40 (s, 3H), 1.48 (s, 9H). LRMS (ESI) *m/z*: 514.20 [M + H]^+^.

#### 4.1.34. 3-amino-5-(4-((4-methylpyrimidin-2-yl)oxy)phenyl)-1-(4-nitrophenyl)-1H-pyrazole-4-carbonitrile (M8-8)

**M8-8** (3.17 g, 71.6%, yellow solid) was synthesised from **M8-7** (5.5 g, 10.71 mmol) in a similar manner as **M1-8**. ^1^H NMR (500 MHz, DMSO-*d_6_*) δ 8.46 (dd, *J* = 5.0, 2.4 Hz, 1H), 8.33 (d, *J* = 9.0 Hz, 1H), 8.22 (d, *J* = 9.1 Hz, 1H), 7.67 (d, *J* = 9.0 Hz, 1H), 7.49 (d, *J* = 8.6 Hz, 1H), 7.44 (t, *J* = 8.6 Hz, 2H), 7.33 (dd, *J* = 11.5, 8.6 Hz, 2H), 7.17 (d, *J* = 5.0 Hz, 1H), 2.40 (s, 3H). LRMS (ESI) *m/z*: 414.15 [M + H]^+^.

#### 4.1.35. 3-(4-((4-methylpyrimidin-2-yl)oxy)phenyl)-2-(4-nitrophenyl)-2H-pyrazolo[3,4-d]pyrimidin-4-amine (M8-9)

**M8-9** (753 mg, 22.3%, yellow solid) was synthesised from **M8-8** (3.17 g, 7.67 mmol) in a similar manner as **M1-9**. ^1^H NMR (500 MHz, DMSO-*d_6_*) δ 8.48 (d, *J* = 5.0 Hz, 1H), 8.30 (s, 1H), 8.28 (d, *J* = 9.0 Hz, 2H), 7.65 (d, *J* = 9.0 Hz, 2H), 7.54 (d, *J* = 8.6 Hz, 2H), 7.34 (d, *J* = 8.6 Hz, 2H), 7.18 (d, *J* = 5.0 Hz, 1H), 2.40 (s, 3H). LRMS (ESI) *m/z*: 441.20 [M + H]^+^.

*4.1.36. 2-(4-aminophenyl)-3-(4-((4-methylpyrimidin-2-yl)oxy)phenyl)-2H-pyrazolo[3,4-d]pyrimidin-4-amine (****M8-10****)***M8-10** (557 mg, 79.5%, yellow solid) was synthesised from **M8-9** (753 mg, 1.71 mmol) in a similar manner as **M1-10**. ^1^H NMR (500 MHz, DMSO-*d_6_*) δ 8.47 (d, *J* = 5.0 Hz, 1H), 8.24 (s, 1H), 7.44 (d, *J* = 8.6 Hz, 2H), 7.29 (d, *J* = 8.6 Hz, 2H), 7.17 (d, *J* = 5.0 Hz, 1H), 6.99 (d, *J* = 8.6 Hz, 2H), 6.50 (d, *J* = 8.6 Hz, 2H), 2.40 (s, 3H). LRMS (ESI) *m/z*: 411.20 [M + H]^+^.

#### 4.1.37. N-(4-(4-amino-3-(4-((4-methylpyrimidin-2-yl)oxy)phenyl)-2H-pyrazolo[3,4-d]pyrimidin-2-yl)phenyl)acrylamide (PLW8)

**PLW8** (64 mg, 19%, white solid) was synthesised from **M8-10** (300 mg, 0.73 mmol) in a similar manner as **PLW1**. **^1^H NMR** (500 MHz, DMSO-*d_6_*) δ 10.36 (s, 1H), 8.49 (d, *J* = 5.0 Hz, 1H), 8.29 (s, 1H), 7.72 (d, *J* = 8.9 Hz, 2H), 7.49 (d, *J* = 8.6 Hz, 2H), 7.36 (d, *J* = 8.9 Hz, 2H), 7.32 (d, *J* = 8.6 Hz, 2H), 7.18 (d, *J* = 5.1 Hz, 1H), 6.43 (dd, *J* = 17.0, 10.1 Hz, 1H), 6.28 (dd, *J* = 17.0, 1.9 Hz, 1H), 5.78 (dd, *J* = 10.1, 1.9 Hz, 1H), 2.41 (s, 3H). **^13^C NMR** (126 MHz, DMSO-*d_6_*) δ 171.06, 164.47, 163.86, 160.07, 159.75, 159.50, 157.26, 154.35, 139.73, 136.64, 134.56, 132.06, 132.01, 127.93, 127.20, 125.62, 122.73, 119.73, 117.19, 101.58, 24.04. ESI-HRMS *m/z:* calcd. For C_25_H_20_N_8_O_2_ [M + H]^+^ 465.1782; found: 465.1788. HPLC: 98.6849%.

#### 4.1.38. Ethyl 4-hydroxy-3-methylbenzoate (M9-1)

**M9-1** (10.93 g, 92.3%, white solid) was synthesised from 4-hydroxy-3-methylbenzoic acid (10 g, 65.72 mmol) in a similar manner as **M1-1**. ^1^H NMR (500 MHz, CDCl_3_) δ 7.84 (d, *J* = 1.4 Hz, 1H), 7.79 (dd, *J* = 8.4, 2.1 Hz, 1H), 6.84 (d, *J* = 8.4 Hz, 1H), 4.35 (q, *J* = 7.1 Hz, 2H), 2.27 (s, 3H), 1.38 (t, *J* = 7.1 Hz, 3H). LRMS (ESI) *m/z*: 181.20 [M + H]^+^.

#### 4.1.39. Ethyl 3-methyl-4-((4-methylpyrimidin-2-yl)oxy)benzoate (M9-2)

**M9-2** (10.93 g, 66.2%, white solid) was synthesised from **M9-1** (10.93 g, 60.66 mmol) in a similar manner as **M1-2**. ^1^H NMR (500 MHz, CDCl_3_) δ 8.32 (d, *J* = 5.0 Hz, 1H), 7.98 (d, *J* = 1.4 Hz, 1H), 7.93 (dd, *J* = 8.4, 2.0 Hz, 1H), 7.14 (d, *J* = 8.4 Hz, 1H), 6.90 (d, *J* = 5.0 Hz, 1H), 4.36 (q, *J* = 7.1 Hz, 2H), 2.49 (s, 3H), 2.23 (s, 3H), 1.37 (t, *J* = 7.1 Hz, 3H). LRMS (ESI) *m/z:* 273.20 [M + H]^+^.

*3-(3-methyl-4-((4-methylpyrimidin-2-yl)oxy)phenyl)-3-oxopropanenitrile (****M9-3****):* **M9-3** (7.51 g, 70%, white solid) was synthesised from **M9-2** (10.93 g, 40.15 mmol) in a similar manner as **M1-3**. ^1^H NMR (500 MHz, CDCl_3_) δ 8.34 (d, *J* = 5.0 Hz, 1H), 7.87 (d, *J* = 1.5 Hz, 1H), 7.80 (dd, *J* = 8.4, 2.2 Hz, 1H), 7.23 (d, *J* = 8.4 Hz, 1H), 6.95 (d, *J* = 5.0 Hz, 1H), 4.08 (s, 2H), 2.51 (s, 3H), 2.27 (s, 3H). LRMS (ESI) *m/z*: 268.20 [M + H]^+^.

#### 4.1.40. ethyl4-cyano-5-(3-methyl-4-((4-methylpyrimidin-2-yl)oxy)phenyl)-1-(4-nitrophenyl)-1H-pyrazole-3-carboxylate (M9-5)

**M9-5** (7.08 g, 52%, yellow solid) was synthesised from **M9-3** (7.51 g, 28.1 mmol) and **M1-4** (9.16 g, 33.72 mmol) in a similar manner as **M1-5**. ^1^H NMR (500 MHz, CDCl_3_) δ 8.35 (d, *J* = 5.0 Hz, 1H), 8.27 (d, *J* = 9.0 Hz, 2H), 7.57 (d, *J* = 9.0 Hz, 2H), 7.32 (d, *J* = 1.6 Hz, 1H), 7.13 (m, 2H), 6.94 (d, *J* = 5.0 Hz, 1H), 4.54 (q, *J* = 7.1 Hz, 2H), 2.50 (s, 3H), 2.20 (s, 3H), 1.48 (t, *J* = 7.1 Hz, 3H). LRMS (ESI) *m/z*: 485.20 [M + H]^+^.

#### 4.1.41. 4-cyano-5-(3-methyl-4-((4-methylpyrimidin-2-yl)oxy)phenyl)-1-(4-nitrophenyl)-1H-pyrazole-3-carboxylic acid (M9-6)

**M9-6** (4.38 g, 65.7%, yellow solid) was synthesised from **M9-5** (7.08 g, 14.61 mmol) in a similar manner as **M1-6**. ^1^H NMR (500 MHz, DMSO-*d_6_*) δ 8.43 (d, *J* = 5.0 Hz, 1H), 8.32 (d, *J* = 9.1 Hz, 2H), 7.65 (d, *J* = 9.0 Hz, 2H), 7.44 (d, *J* = 1.4 Hz, 1H), 7.24 (dt, *J* = 15.7, 5.1 Hz, 2H), 7.14 (d, *J* = 5.0 Hz, 1H), 2.39 (s, 3H), 2.04 (s, 3H). LRMS (ESI) *m/z*: 457.15 [M + H]^+^.

#### 4.1.42. Tert-butyl(4-cyano-5-(3-methyl-4-((4-methylpyrimidin-2-yl)oxy)phenyl)-1-(4-nitrophenyl)-1H-pyrazol-3-yl)carbamate (M9-7)

**M9-7** (2.92 g, 57.7%, yellow solid) was synthesised from **M9-6** (4.38 g, 9.6 mmol) in a similar manner as **M1-7**. ^1^H NMR (500 MHz, DMSO-*d_6_*) δ 10.14 (s, 1H), 8.44 (d, *J* = 5.0 Hz, 1H), 8.28 (d, *J* = 9.0 Hz, 2H), 7.54 (d, *J* = 9.0 Hz, 2H), 7.42 (s, 1H), 7.23 (s, 2H), 7.15 (d, *J* = 5.0 Hz, 1H), 2.40 (s, 3H), 2.05 (s, 3H), 1.48 (s, 9H). LRMS (ESI) *m/z*: 528.25 [M + H]^+^.

#### 4.1.43. 3-amino-5-(3-methyl-4-((4-methylpyrimidin-2-yl)oxy)phenyl)-1-(4-nitrophenyl)-1H-pyrazole-4-carbonitrile (M9-8)

**M9-8** (1.6 g, 67.7%, yellow solid) was synthesised from **M9-7** (2.92 g, 5.54 mmol) in a similar manner as **M1-8**. ^1^H NMR (500 MHz, DMSO-*d_6_*) δ 8.44 (d, *J* = 5.0 Hz, 1H), 8.22 (d, *J* = 9.1 Hz, 2H), 7.44 (d, *J* = 9.1 Hz, 2H), 7.39 (s, 1H), 7.23–7.19 (m, 2H), 7.15 (d, *J* = 5.0 Hz, 1H), 2.41 (s, 3H), 2.05 (s, 3H). LRMS (ESI) *m/z*: 428.15 [M + H]^+^.

#### 4.1.44. 3-(3-methyl-4-((4-methylpyrimidin-2-yl)oxy)phenyl)-2-(4-nitrophenyl)-2H-pyrazolo[3,4-d]pyrimidin-4-amine (M9-9)

**M9-9** (518 mg, 30.4%, yellow solid) was synthesised from **M9-8** (1.6 g, 3.75 mmol) in a similar manner as **M1-9**. ^1^H NMR (500 MHz, DMSO-*d_6_*) δ 8.45 (d, *J* = 5.0 Hz, 1H), 8.30 (s, 1H), 8.28 (d, *J* = 9.1 Hz, 2H), 7.66 (d, *J* = 9.0 Hz, 2H), 7.50 (d, *J* = 1.7 Hz, 1H), 7.32 (dd, *J* = 8.3, 2.1 Hz, 1H), 7.23 (d, *J* = 8.3 Hz, 1H), 7.15 (d, *J* = 5.0 Hz, 1H), 2.39 (s, 3H), 2.07 (s, 3H). LRMS (ESI) *m/z*: 455.15 [M + H]^+^.

#### 4.1.45. 2-(4-aminophenyl)-3-(3-methyl-4-((4-methylpyrimidin-2-yl)oxy)phenyl)-2H-pyrazolo[3,4-d]pyrimidin-4-amine (M9-10)

**M9-10** (375 mg, 77.7%, white solid) was synthesised from **M9-9** (518 mg, 1.14 mmol) in a similar manner as **M1-10**. ^1^H NMR (500 MHz, DMSO-*d_6_*) δ 8.44 (d, *J* = 5.0 Hz, 1H), 8.23 (s, 1H), 7.39 (d, *J* = 1.4 Hz, 1H), 7.24 (dd, *J* = 8.3, 1.9 Hz, 1H), 7.19 (d, *J* = 8.3 Hz, 1H), 7.14 (d, *J* = 5.0 Hz, 1H), 7.00 (d, *J* = 8.6 Hz, 2H), 6.50 (d, *J* = 8.7 Hz, 2H), 2.40 (s, 3H), 2.06 (s, 3H). LRMS (ESI) *m/z*: 425.25 [M + H]^+^.

#### 4.1.46. N-(4-(4-amino-3-(3-methyl-4-((4-methylpyrimidin-2-yl)oxy)phenyl)-2H-pyrazolo[3,4-d]pyrimidin-2-yl)phenyl)acrylamide (PLW9)

**PLW9** (88 mg, 20.8%, white solid) was synthesised from **M9-10** (375 mg, 0.88 mmol) in a similar manner as **PLW1**. **^1^H NMR** (500 MHz, DMSO-*d_6_*) δ 10.45 (s, 1H), 8.46 (d, *J* = 5.0 Hz, 1H), 8.33 (s, 1H), 7.74 (d, *J* = 8.9 Hz, 2H), 7.45 (d, *J* = 1.7 Hz, 1H), 7.37 (d, *J* = 8.9 Hz, 2H), 7.28 (dd, *J* = 8.3, 2.0 Hz, 1H), 7.22 (d, *J* = 8.3 Hz, 1H), 7.16 (d, *J* = 5.0 Hz, 1H), 6.46 (dd, *J* = 17.0, 10.2 Hz, 1H), 6.28 (dd, *J* = 17.0, 1.9 Hz, 1H), 5.78 (dd, *J* = 10.1, 1.9 Hz, 1H), 2.41 (s, 3H), 2.08 (s, 3H). **^13^C NMR** (126 MHz, DMSO-*d_6_*) δ 171.13, 164.37, 163.89, 159.83, 159.79, 158.44, 156.26, 152.88, 139.83, 137.33, 134.43, 133.16, 132.08, 131.76, 129.55, 127.90, 127.10, 125.66, 123.32, 119.72, 116.89, 101.35, 24.05, 16.53. ESI-HRMS *m/z*: calcd. For C_26_H_22_N_8_O_2_ [M + H]^+^ 479.1938; found: 479.1943. HPLC: 99.1140%.

#### 4.1.47. Ethyl 4-cyclobutoxy-3-fluorobenzoate (M10-2)

**M1-1** (10 g, 54.29 mmol), bromobutane (21.98 g, 162.87 mmol), Cs_2_CO_3_ (53.07 g, 162.87 mmol), and KI (0.9 g, 5.43 mmol) were combined in a flask with DMF (150 ml). The reaction mixture was stirred at 80 °C for 2 h. Upon completion, water was added, and the mixture was extracted three times with ethyl acetate. The combined organic layers were dried over anhydrous Na_2_SO_4_, filtered, and concentrated under reduced pressure. Subsequently, the resulting residue was purified by silica gel chromatography using PE/EA(v/v = 5:1) as eluent to obtain white solid **M10-2** (9.99 g, 77.3%). ^1^H NMR (500 MHz, CDCl_3_) δ 7.80–7.71 (m, 2H), 6.81 (t, *J* = 8.3 Hz, 1H), 4.73 (p, *J* = 7.2 Hz, 1H), 4.33 (q, *J* = 7.1 Hz, 2H), 2.52–2.43 (m, 2H), 2.30–2.19 (m, 2H), 1.93–1.85 (m, 1H), 1.77–1.65 (m, 1H), 1.36 (t, *J* = 7.1 Hz, 3H). LRMS (ESI) *m/z*: 239.20 [M + H]^+^.

#### 4.1.48. 3-(4-cyclobutoxy-3-fluorophenyl)-3-oxopropanenitrile (M10-3)

**M10-3** (6.15 g, 62.8%, white solid) was synthesised from **M10-2** (9.99 g, 41.96 mmol) in a similar manner as **M1-3**. ^1^H NMR (500 MHz, CDCl_3_) δ 7.69–7.61 (m, 2H), 6.87 (t, *J* = 8.3 Hz, 1H), 4.77 (p, *J* = 7.1 Hz, 1H), 4.00 (d, *J* = 1.1 Hz, 2H), 2.54–2.45 (m, 2H), 2.32–2.21 (m, 2H), 1.98–1.88 (m, 1H), 1.79–1.68 (m, 1H). LRMS (ESI) *m/z*: 234.15 [M + H]^+^.

#### 4.1.49. ethyl4-cyano-5-(4-cyclobutoxy-3-fluorophenyl)-1-(4-nitrophenyl)-1H-pyrazole-3-carboxylate (M10-5)

**M10-5** (6.28 g, 52.9%, yellow solid) was synthesised from **M10-3** (6.15 g, 26.35 mmol) and **M1-4** (8.59 g, 31.62 mmol) in a similar manner as **M1-5**. ^1^H NMR (500 MHz, CDCl_3_) δ 8.26 (d, *J* = 8.9 Hz, 2H), 7.52 (d, *J* = 8.9 Hz, 2H), 7.08 (d, *J* = 9.0 Hz, 1H), 6.98 (dd, *J* = 11.0, 2.1 Hz, 1H), 6.90–6.86 (m, 1H), 4.74–4.67 (m, 1H), 4.52 (q, *J* = 7.1 Hz, 2H), 2.53–2.43 (m, 3H), 2.30–2.21 (m, 3H), 1.46 (t, *J* = 7.1 Hz, 3H). LRMS (ESI) *m/z*: 451.20 [M + H]^+^.

#### 4.1.50. 4-cyano-5-(4-cyclobutoxy-3-fluorophenyl)-1-(4-nitrophenyl)-1H-pyrazole-3-carboxylic acid (M10-6)

**M10-6** (4.26 g, 72.4%, yellow solid) was synthesised from **M10-5** (6.28 g, 13.94 mmol) in a similar manner as **M1-6**. ^1^H NMR (500 MHz, DMSO-*d_6_*) δ 8.28 (d, *J* = 9.1 Hz, 2H), 7.58 (d, *J* = 9.0 Hz, 2H), 7.30 (dd, *J* = 11.8, 2.0 Hz, 1H), 7.16 (dd, *J* = 8.5, 1.6 Hz, 1H), 7.11 (t, *J* = 8.5 Hz, 1H), 4.81–4.74 (m, 1H), 2.51–2.49 (m, 1H), 2.46–2.39 (m, 3H), 2.13–2.01 (m, 3H). LRMS (ESI) *m/z*: 421.10 [M - H]**^-^**.

#### 4.1.51. Tert-butyl(4-cyano-5-(4-cyclobutoxy-3-fluorophenyl)-1-(4-nitrophenyl)-1H-pyrazol-3-yl)carbamate (M10-7)

**M10-7** (2.78 g, 55.8%, yellow solid) was synthesised from **M10-6** (4.26 g, 10.09 mmol) in a similar manner as **M1-7**. ^1^H NMR (500 MHz, DMSO-*d_6_*) δ 10.13 (s, 1H), 8.27 (d, *J* = 9.1 Hz, 2H), 7.51 (d, *J* = 9.1 Hz, 2H), 7.30 (dd, *J* = 11.7, 2.0 Hz, 1H), 7.17 (dd, *J* = 8.6, 1.8 Hz, 1H), 7.12 (t, *J* = 8.5 Hz, 1H), 4.82–4.75 (m, 1H), 2.51–2.49 (m, 1H), 2.46–2.40 (m, 2H), 2.13–2.02 (m, 3H), 1.47 (s, 9H). LRMS (ESI) *m/z*: 492.20 [M–H]**^–^**.

#### 4.1.52. 3-amino-5-(4-cyclobutoxy-3-fluorophenyl)-1-(4-nitrophenyl)-1H-pyrazole-4-carbonitrile (M10-8)

**M10-8** (1.45 g, 65.6%, yellow solid) was synthesised from **M10-7** (2.78 g, 5.63 mmol) in a similar manner as **M1-8**. ^1^H NMR (500 MHz, DMSO-*d_6_*) δ 8.32 (d, *J* = 9.0 Hz, 2H), 7.63 (d, *J* = 9.1 Hz, 2H), 7.32 (dd, *J* = 11.7, 2.0 Hz, 1H), 7.20 (dd, *J* = 8.9, 1.9 Hz, 1H), 7.13 (t, *J* = 8.5 Hz, 1H), 4.82–4.75 (m, 1H), 2.51–2.49 (m, 1H), 2.46–2.40 (m, 2H), 2.12–2.02 (m, 3H). LRMS (ESI) *m/z*: 394.20 [M + H]^+^.

#### 4.1.53. 3-(4-cyclobutoxy-3-fluorophenyl)-2-(4-nitrophenyl)-2H-pyrazolo[3,4-d]pyrimidin-4-amine (M10-9)

**M10-9** (299 mg, 19.3%, yellow solid) was synthesised from **M10-8** (1.45 g, 3.69 mmol) in a similar manner as **M1-9**. ^1^H NMR (500 MHz, DMSO-*d_6_*) δ 8.28 (s, 1H), 8.26 (d, *J* = 2.3 Hz, 2H), 7.60 (d, *J* = 9.0 Hz, 2H), 7.40 (dd, *J* = 11.7, 2.0 Hz, 1H), 7.16 (dd, *J* = 8.5, 1.3 Hz, 1H), 7.08 (t, *J* = 8.6 Hz, 1H), 4.80–4.73 (m, 1H), 2.47–2.39 (m, 2H), 2.14–2.04 (m, 2H), 1.84–1.75 (m, 1H), 1.68–1.57 (m, 1H). LRMS (ESI) *m/z*: 421.15 [M + H]^+^.

#### 4.1.54. 2-(4-aminophenyl)-3-(4-cyclobutoxy-3-fluorophenyl)-2H-pyrazolo[3,4-d]pyrimidin-4-amine (M10-10)

**M10-10** (204 mg, 73.9%, white solid) was synthesised from **M10-9** (299 mg, 0.71 mmol) in a similar manner as **M1-10**. ^1^H NMR (500 MHz, DMSO-*d_6_*) δ 8.23 (s, 1H), 7.23 (dd, *J* = 11.8, 1.8 Hz, 1H), 7.08 (dd, *J* = 8.5, 1.8 Hz, 1H), 7.05 (t, *J* = 8.3 Hz, 1H), 6.94 (d, *J* = 8.7 Hz, 2H), 6.48 (d, *J* = 8.7 Hz, 2H), 4.79–4.72 (m, 1H), 2.45–2.40 (m, 2H), 2.11–2.03 (m, 2H), 1.83–1.74 (m, 1H), 1.65–1.58 (m, 1H). LRMS (ESI) *m/z*: 391.20 [M + H]^+^.

#### 4.1.55. N-(4-(4-amino-3-(4-cyclobutoxy-3-fluorophenyl)-2H-pyrazolo[3,4-d]pyrimidin-2-yl)phenyl)acrylamide (PLW10)

**PLW10** (22 mg, 9.6%, white solid) was synthesised from **M10-10** (204 mg, 0.52 mmol) in a similar manner as **PLW1**. **^1^H NMR** (600 MHz, DMSO-*d_6_*) δ 10.37 (s, 1H), 8.26 (s, 1H), 7.70 (d, *J* = 8.9 Hz, 2H), 7.32 (s, 1H), 7.31 (d, *J* = 2.8 Hz, 2H), 7.13 (dd, *J* = 8.4, 1.3 Hz, 1H), 7.07 (t, *J* = 8.6 Hz, 1H), 6.44 (dd, *J* = 17.0, 10.2 Hz, 1H), 6.28 (dd, *J* = 17.0, 1.7 Hz, 1H), 5.78 (dd, *J* = 10.2, 1.7 Hz, 1H), 4.80–4.74 (m, 1H), 2.47–2.41 (m, 2H), 2.14–2.06 (m, 2H), 1.83–1.76 (m, 1H), 1.68–1.59 (m, 1H). **^13^C NMR** (151 MHz, DMSO-*d_6_*) δ 162.79, 158.93, 158.27, 156.05, 150.68 (d, *J* = 245.4 Hz), 145.63 (d, *J* = 10.1 Hz), 138.61, 135.02, 133.46, 131.01, 126.81, 126.45, 126.05, 120.08 (d, *J* = 7.8 Hz), 118.67, 117.40 (d, *J* = 19.4 Hz), 114.94, 100.43, 71.35, 29.41, 12.07. ESI-HRMS *m/z*: calcd. For C_24_H_21_FN_6_O_2_ [M + H]^+^ 445.1783; found: 445.1790. HPLC: 95.3016%.

#### 4.1.56. 4-(Cyclohexyloxy)-3-fluorobenzoic acid (M11-1)

A mixture of 3,4-difluorobenzoic acid (10 g, 63.25 mmol), cyclohexanol (12.67 g, 126.5 mmol), and NaOH (5.06 g, 126.5 mmol) in DMSO (200 ml) was stirred at 100 °C for 2 h. After cooling to room temperature, the reaction mixture was diluted with water and extracted with ethyl acetate (3 × 300 ml). The combined organic layers were washed with saturated NaCl solution, dried over anhydrous Na_2_SO_4_, filtered, and concentrated under reduced pressure. Subsequently, the resulting residue was purified by silica gel chromatography using PE/EA(v/v = 5:1) as eluent to obtain white solid **M11-1** (12.05 g, 80%). ^1^H NMR (500 MHz, CDCl_3_) δ 7.86–7.82 (m, 1H), 7.80 (dd, *J* = 11.5, 2.0 Hz, 1H), 7.01 (t, *J* = 8.3 Hz, 1H), 4.43–4.36 (m, 1H), 2.05–1.97 (m, 2H), 1.88–1.79 (m, 2H), 1.68–1.55 (m, 3H), 1.45–1.32 (m, 3H). LRMS (ESI) *m/z*: 239.15 [M + H]^+^.

#### 4.1.57. Ethyl 4-(cyclohexyloxy)-3-fluorobenzoate (M11-2)

A solution of **M11-1** (12.05 g, 50.58 mmol) in ethanol (150 ml) was treated dropwise with hionyl chloride (SOCl_2_, 30 ml) at room temperature. After complete addition, the mixture was heated to 80 °C for 1 h. Upon reaction completion, the solvent was removed under reduced pressure to eliminate excess ethanol. Subsequently, the resulting residue was purified by silica gel chromatography using PE/EA(v/v = 5:1) as eluent to afford the white solid **M11-2** (12.12 g, 90%). ^1^H NMR (500 MHz, CDCl_3_) δ 7.79 (d, *J* = 8.6 Hz, 1H), 7.73 (dd, *J* = 11.8, 2.0 Hz, 1H), 6.95 (t, *J* = 8.4 Hz, 1H), 4.98 (ddd, *J* = 12.6, 8.6, 3.8 Hz, 1H), 4.15 (q, *J* = 7.0 Hz, 2H), 1.96–1.88 (m, 2H), 1.83–1.73 (m, 2H), 1.61–1.52 (m, 3H), 1.47 (t, *J* = 7.0 Hz, 3H), 1.45–1.32 (m, 3H). LRMS (ESI) *m/z*: 267.20 [M + H]^+^.

#### 4.1.58. 3-(4-(Cyclohexyloxy)-3-fluorophenyl)-3-oxopropanenitrile (M11-3)

**M11-3** (7.44 g, 62.6%, white solid) was synthesised from **M11-2** (12.12 g, 45.51 mmol) in a similar manner as **M11-3**. ^1^H NMR (500 MHz, CDCl_3_) δ 7.67–7.62 (m, 2H), 7.03 (t, *J* = 8.1 Hz, 1H), 4.46–4.39 (m, 1H), 4.00 (s, 2H), 2.03–1.96 (m, 2H), 1.87–1.79 (m, 2H), 1.67–1.55 (m, 3H), 1.44–1.32 (m, 3H). LRMS (ESI) *m/z*: 262.20 [M + H]^+^.

#### 4.1.59. ethyl4-cyano-5-(4-(cyclohexyloxy)-3-fluorophenyl)-1-(4-nitrophenyl)-1H-pyrazole-3-carboxylate (M11-5)

**M11-5** (6.08 g, 44.6%, yellow solid) was synthesised from **M11-3** (7.44 g, 28.49 mmol) and **M1-4** (9.28 g, 34.19 mmol) in a similar manner as **M1-5**. ^1^H NMR (500 MHz, CDCl_3_) δ 8.26 (d, *J* = 9.1 Hz, 2H), 7.53 (d, *J* = 9.0 Hz, 2H), 7.06 (dd, *J* = 8.7, 2.0 Hz, 1H), 7.03 (d, *J* = 7.9 Hz, 1H), 7.02–6.98 (m, 1H), 4.51 (q, *J* = 7.1 Hz, 2H), 4.36–4.29 (m, 1H), 2.01–1.94 (m, 2H), 1.85–1.78 (m, 2H), 1.63–1.57 (m, 2H), 1.46 (t, *J* = 7.1 Hz, 3H), 1.42–1.29 (m, 4H). LRMS (ESI) *m/z*: 479.20 [M + H]^+^.

#### 4.1.60. 4-cyano-5-(4-(cyclohexyloxy)-3-fluorophenyl)-1-(4-nitrophenyl)-1H-pyrazole-3-carboxylic acid (M11-6)

**M11-6** (4.29 g, 75%, yellow solid) was synthesised from **M11-5** (6.08 g, 12.7 mmol) in a similar manner as **M1-6**. ^1^H NMR (500 MHz, DMSO-*d_6_*) δ 8.31 (d, *J* = 8.9 Hz, 2H), 7.63 (d, *J* = 8.9 Hz, 2H), 7.35–7.29 (m, 2H), 7.16 (d, *J* = 8.7 Hz, 1H), 4.48–4.41 (m, 1H), 1.95–1.87 (m, 2H), 1.73–1.64 (m, 2H), 1.53–1.19 (m, 6H). LRMS (ESI) *m/z*: 449.10 [M - H]^-^.

#### 4.1.61. Tert-butyl (4-cyano-5-(4-(cyclohexyloxy)-3-fluorophenyl)-1-(4-nitrophenyl)-1H-pyrazol-3-yl)carbamate (M11-7)

**M11-7** (2.68 g, 54%, yellow solid) was synthesised from **M11-6** (4.29 g, 9.53 mmol) in a similar manner as **M1-7**. ^1^H NMR (500 MHz, DMSO-*d_6_*) δ 10.11 (s, 1H), 8.27 (d, *J* = 9.1 Hz, 2H), 7.52 (d, *J* = 9.0 Hz, 2H), 7.35–7.28 (m, 2H), 7.14 (dd, *J* = 8.6, 0.9 Hz, 1H), 4.49–4.42 (m, 1H), 1.96–1.89 (m, 2H), 1.74–1.66 (m, 2H), 1.48 (s, 9H), 1.45–1.20 (m, 6H). LRMS (ESI) *m/z*: 522.45 [M + H]^+^.

#### 4.1.62. 3-amino-5-(4-(cyclohexyloxy)-3-fluorophenyl)-1-(4-nitrophenyl)-1H-pyrazole-4-carbonitrile (M11-8)

**M11-8** (1.52 g, 70.2%, yellow solid) was synthesised from **M11-7** (2.68 g, 5.15 mmol) in a similar manner as **M1-8**. ^1^H NMR (500 MHz, DMSO-*d_6_*) δ 8.22 (d, *J* = 9.1 Hz, 2H), 7.43 (d, *J* = 9.1 Hz, 2H), 7.31 (t, *J* = 6.3 Hz, 1H), 7.28 (dd, *J* = 9.4, 2.3 Hz, 1H), 7.08 (dd, *J* = 13.5, 8.6 Hz, 1H), 6.12 (s, 2H), 4.49–4.42 (m, 1H), 1.95–1.89 (m, 2H), 1.73–1.67 (m, 2H), 1.48–1.31 (m, 6H). LRMS (ESI) *m/z*: 422.20 [M + H]^+^.

#### 4.1.63. 3-4-(Cyclohexyloxy)-3-fluorophenyl)-2-(4-nitrophenyl)-2H-pyrazolo[3,4-d]pyrimidin-4-amine (M11-9)

**M11-9** (384 mg, 23.7%, yellow solid) was synthesised from **M11-8** (1.52 g, 3.62 mmol) in a similar manner as **M1-9**. ^1^H NMR (500 MHz, DMSO-*d_6_*) δ 8.28 (s, 1H), 8.27 (d, *J* = 8.1 Hz, 2H), 7.61 (d, *J* = 9.1 Hz, 2H), 7.56–7.49 (m, 1H), 7.35 (d, *J* = 8.9 Hz, 1H), 7.28–7.25 (m, 1H), 4.48–4.38 (m, 1H), 1.96–1.82 (m, 2H), 1.72–1.60 (m, 2H), 1.52–1.20 (m, 6H). LRMS (ESI) *m/z*: 449.20 [M + H]^+^.

#### 4.1.64. 2-(4-aminophenyl)-3-(4-(cyclohexyloxy)-3-fluorophenyl)-2H-pyrazolo[3,4-d]pyrimidin-4-amine (M11-10)

**M11-10** (286 mg, 79.7%, white solid) was synthesised from **M11-9** (384 mg, 0.86 mmol) in a similar manner as **M1-10**. ^1^H NMR (500 MHz, DMSO-*d_6_*) δ 8.21 (s, 1H), 7.35 (d, *J* = 9.0 Hz, 1H), 7.27–7.23 (m, 1H), 7.23–7.20 (m, 1H), 6.95 (d, *J* = 8.7 Hz, 2H), 6.49 (d, *J* = 8.6 Hz, 2H), 5.41 (s, 2H), 4.45–4.40 (m, 1H), 1.97–1.88 (m, 2H), 1.73–1.66 (m, 2H), 1.53–1.40 (m, 6H). LRMS (ESI) *m/z*: 419.25 [M + H]^+^.

#### 4.1.65. N-(4-(4-amino-3-(4-(cyclohexyloxy)-3-fluorophenyl)-2H-pyrazolo[3,4-d]pyrimidin-2-yl)phenyl)acrylamide (PLW11)

**PLW11** (61 mg, 19%, white solid) was synthesised from **M11-10** (286 mg, 0.69 mmol) in a similar manner as **PLW1**. **^1^H NMR** (500 MHz, DMSO-*d_6_*) δ 10.34 (s, 1H), 8.26 (s, 1H), 7.69 (d, *J* = 8.9 Hz, 2H), 7.31 (d, *J* = 8.8 Hz, 2H), 7.29 (d, *J* = 3.7 Hz, 1H), 7.28–7.25 (m, 1H), 7.11 (dd, *J* = 8.5, 1.1 Hz, 1H), 6.43 (dd, *J* = 17.0, 10.1 Hz, 1H), 6.27 (dd, *J* = 17.0, 1.9 Hz, 1H), 5.78 (dd, *J* = 10.1, 1.9 Hz, 1H), 4.46–4.40 (m, 1H), 1.97–1.92 (m, 2H), 1.74–1.69 (m, 2H), 1.53–1.44 (m, 3H), 1.42–1.31 (m, 3H). **^13^C NMR** (126 MHz, DMSO-*d_6_*) δ 163.26, 159.45, 158.88, 156.64, 151.91 (d, *J* = 245.2 Hz), 146.26 (d, *J* = 10.1 Hz), 139.05, 135.48, 133.99, 131.47, 127.34, 126.83, 126.56, 120.45 (d, *J* = 7.3 Hz), 118.01 (d, *J* = 19.9 Hz), 119.13, 116.66, 100.86, 75.97, 31.13, 24.83, 23.02. ESI-HRMS *m/z*: calcd. For C_26_H_25_FN_6_O_2_ [M + H]^+^ 473.2096; found: 473.2100. HPLC: 95.2631%.

#### 4.1.66. Ethyl 3-fluoro-4-(pyrimidin-2-yloxy)benzoate (M12-2)

**M12-2** (12.67 g, 89%, white solid) was synthesised from **M1-1** (10 g, 52.3 mmol) in a similar manner as **M1-2**. ^1^H NMR (500 MHz, CDCl_3_) δ 8.57 (d, *J* = 4.8 Hz, 2H), 7.94–7.91 (m, 1H), 7.88 (dd, *J* = 10.6, 1.9 Hz, 1H), 7.37–7.32 (t, 1H), 7.10 (t, *J* = 4.8 Hz, 1H), 4.39 (q, *J* = 7.1 Hz, 2H), 1.39 (t, *J* = 7.1 Hz, 3H). LRMS (ESI) *m/z*: 263.15 [M + H]^+^.

#### 4.1.67. 3-(3-fluoro-4-(pyrimidin-2-yloxy)phenyl)-3-oxopropanenitrile (M12-3)

**M12-3** (7.43 g, 59.8%, white solid) was synthesised from **M12-2** (12.67 g, 48.31 mmol) in a similar manner as **M1-3**. ^1^H NMR (500 MHz, CDCl_3_) δ 8.58 (d, *J* = 4.8 Hz, 2H), 7.82–7.77 (m, 2H), 7.46 (dd, *J* = 8.6, 7.4 Hz, 1H), 7.15 (t, *J* = 4.8 Hz, 1H), 4.09 (s, 2H). LRMS (ESI) *m/z*: 258.15 [M + H]^+^.

#### 4.1.68. Ethyl 4-cyano-5-(3-fluoro-4-(pyrimidin-2-yloxy)phenyl)-1-(4-nitrophenyl)-1H-pyrazole-3-carboxylate (M12-5)

**M12-5** (11.88 g, 86.7%, yellow solid) was synthesised from **M12-3** (7.43 g, 28.89 mmol) and **M1-4** (9.4 g, 34.67 mmol) in a similar manner as **M1-5**. ^1^H NMR (500 MHz, CDCl_3_) δ 8.58 (d, *J* = 4.8 Hz, 2H), 8.30 (d, *J* = 8.7 Hz, 2H), 7.57 (d, *J* = 8.7 Hz, 2H), 7.39 (t, *J* = 8.1 Hz, 1H), 7.23–7.17 (m, 2H), 7.14 (t, *J* = 4.7 Hz, 1H), 4.54 (q, *J* = 7.1 Hz, 2H), 1.47 (t, *J* = 7.1 Hz, 3H). LRMS (ESI) *m/z*: 475.30 [M + H]^+^.

#### 4.1.69. 4-cyano-5-(3-fluoro-4-(pyrimidin-2-yloxy)phenyl)-1-(4-nitrophenyl)-1H-pyrazole-3-carboxylic acid (M12-6)

**M12-6** (4.66 g, 41.7%, yellow solid) was synthesised from **M12-5** (11.88 g, 25.04 mmol) in a similar manner as **M1-6**. ^1^H NMR (500 MHz, DMSO-*d_6_*) δ 8.71 (d, *J* = 4.8 Hz, 2H), 8.34 (d, *J* = 9.0 Hz, 2H), 7.66 (d, *J* = 9.0 Hz, 2H), 7.60–7.56 (m, 2H), 7.38–7.32 (m, 2H). LRMS (ESI) *m/z*: 447.10 [M + H]^+^.

#### 4.1.70. 3-amino-5-(3-fluoro-4-(pyrimidin-2-yloxy)phenyl)-1-(4-nitrophenyl)-1H-pyrazole-4-carbonitrile (M12-8)

**M12-8** (3.17 g, 72.9%, yellow solid) was synthesised from **M12-6** (4.66 g, 10.44 mmol) in a similar manner as **M1-7** and **M1-8**. ^1^H NMR (500 MHz, DMSO-*d_6_*) δ 8.71 (d, *J* = 4.8 Hz, 2H), 8.37 (d, *J* = 9.1 Hz, 2H), 7.71 (d, *J* = 9.1 Hz, 2H), 7.61–7.57 (m, 2H), 7.38–7.35 (m, 2H). LRMS (ESI) *m/z*: 418.20 [M + H]^+^.

#### 4.1.71. 3-(3-fluoro-4-(pyrimidin-2-yloxy)phenyl)-2-(4-nitrophenyl)-2H-pyrazolo[3,4-d]pyrimidin-4-amine (M12-9)

**M12-9** (541 mg, 16%, yellow solid) was synthesised from **M12-8** (3.17 g, 7.61 mmol) in a similar manner as **M1-9**. ^1^H NMR (500 MHz, DMSO-*d_6_*) δ 8.69 (d, *J* = 4.8 Hz, 2H), 8.30 (t, *J* = 5.0 Hz, 3H), 7.67 (d, *J* = 9.0 Hz, 2H), 7.62 (dd, *J* = 10.8, 1.6 Hz, 1H), 7.53 (t, *J* = 8.3 Hz, 1H), 7.37–7.33 (m, 2H). LRMS (ESI) *m/z*: 445.15 [M + H]^+^.

#### 4.1.72. 2-(4-aminophenyl)-3-(3-fluoro-4-(pyrimidin-2-yloxy)phenyl)-2H-pyrazolo[3,4-d]pyrimidin-4-amine (M12-10)

**M12-10** (475 mg, 94.3%, white solid) was synthesised from **M12-9** (541 mg, 1.22 mmol) in a similar manner as **M1-10**. ^1^H NMR (500 MHz, DMSO-*d_6_*) δ 8.68 (d, *J* = 4.8 Hz, 2H), 8.24 (s, 1H), 7.50–7.43 (m, 2H), 7.33 (t, *J* = 4.8 Hz, 1H), 7.26 (dd, *J* = 8.3, 1.4 Hz, 1H), 7.01 (d, *J* = 8.7 Hz, 2H), 6.52 (d, *J* = 8.7 Hz, 2H). LRMS (ESI) *m/z*: 415.25 [M + H]^+^.

#### 4.1.73. N-(4-(4-amino-3-(3-fluoro-4-(pyrimidin-2-yloxy)phenyl)-2H-pyrazolo[3,4-d]pyrimidin-2-yl)phenyl)acrylamide (PLW12)

**PLW12** (64 mg, 12%, white solid) was synthesised from **M12-10** (475 mg, 1.15 mmol) in a similar manner as **PLW1**. **^1^H NMR** (500 MHz, DMSO-*d_6_*) δ 10.39 (s, 1H), 8.70 (d, *J* = 4.8 Hz, 2H), 8.29 (s, 1H), 7.74 (d, *J* = 8.9 Hz, 2H), 7.54 (dd, *J* = 11.2, 2.1 Hz, 1H), 7.51 (t, *J* = 8.3 Hz, 1H), 7.38 (d, *J* = 8.9 Hz, 2H), 7.35 (t, *J* = 4.8 Hz, 1H), 7.30 (dd, *J* = 8.3, 1.5 Hz, 1H), 6.44 (dd, *J* = 17.0, 10.1 Hz, 1H), 6.28 (dd, *J* = 17.0, 1.8 Hz, 1H), 5.79 (dd, *J* = 10.1, 1.8 Hz, 1H). **^13^C NMR** (126 MHz, DMSO-*d_6_*) δ 164.00, 163.89, 160.82, 159.96, 159.53, 157.27, 154.26 (d, *J* = 249.2 Hz), 141.29 (d, *J* = 12.1 Hz), 139.85, 135.48, 134.43, 132.06, 130.13, 127.97, 127.46 (d, *J* = 8.7 Hz), 127.33, 125.20, 119.76, 119.41 (d, *J* = 20.1 Hz)118.13, 101.68. ESI-HRMS *m/z*: calcd. For C_24_H_17_FN_8_O_2_ [M + H]^+^ 469.1531; found: 469.1534. HPLC: 96.8633%.

#### 4.1.74. Ethyl 3-fluoro-4-((4-methylpyridin-2-yl)oxy)benzoate (M13-2)

**M13-2** (12.67 g, 84.8%, white solid) was synthesised from **M1-1** (10 g, 54.3 mmol) in a similar manner as **M1-2**. ^1^H NMR (500 MHz, CDCl_3_) δ 7.98 (d, *J* = 5.1 Hz, 1H), 7.87 (t, *J* = 10.8 Hz, 2H), 7.27 (t, *J* = 7.9 Hz, 1H), 6.87–6.82 (m, 2H), 4.37 (q, *J* = 7.1 Hz, 2H), 2.37 (s, 3H), 1.38 (t, *J* = 7.1 Hz, 3H). LRMS (ESI) *m/z*: 276.10 [M + H]^+^.

#### 4.1.75. 3-(3-fluoro-4-((4-methylpyridin-2-yl)oxy)phenyl)-3-oxopropanenitrile (M13-3)

**M13-3** (7.6 g, 61.1%, white solid) was synthesised from **M13-2** (12.67 g, 46.04 mmol) in a similar manner as **M1-3**. ^1^H NMR (500 MHz, CDCl_3_) δ 7.98 (d, *J* = 5.1 Hz, 1H), 7.79–7.71 (m, 2H), 7.37 (t, *J* = 7.9 Hz, 1H), 6.92–6.87 (m, 2H), 4.07 (s, 2H), 2.40 (s, 3H). LRMS (ESI) *m/z*: 271.15 [M + H]^+^.

#### 4.1.76. Ethyl 4-cyano-5-(3-fluoro-4-((4-methylpyridin-2-yl)oxy)phenyl)-1-(4-nitrophenyl)-1H-pyrazole-3-carboxylate (M13-5)

**M13-5** (8.36 g, 61%, yellow solid) was synthesised from **M13-3** (7.6 g, 28.13 mmol) and **M1-4** (9.16 g, 33.76 mmol) in a similar manner as **M1-5**. ^1^H NMR (500 MHz, CDCl_3_) δ 8.30 (d, *J* = 8.9 Hz, 2H), 8.00 (d, *J* = 5.0 Hz, 1H), 7.58 (d, *J* = 8.8 Hz, 2H), 7.34 (t, *J* = 8.4 Hz, 1H), 7.19–7.12 (m, 2H), 6.91 (d, *J* = 4.9 Hz, 1H), 6.87 (s, 1H), 4.55 (q, *J* = 7.2 Hz, 2H), 2.40 (s, 3H), 1.48 (t, *J* = 7.1 Hz, 3H). LRMS (ESI) *m/z*: 488.15 [M + H]^+^.

#### 4.1.77. 4-cyano-5-(3-fluoro-4-((4-methylpyridin-2-yl)oxy)phenyl)-1-(4-nitrophenyl)-1H-pyrazole-3-carboxylic acid (M13-6)

**M13-6** (5.44 g, 69%, yellow solid) was synthesised from **M13-5** (8.36 g, 17.16 mmol) in a similar manner as **C1-6**. ^1^H NMR (500 MHz, DMSO-*d_6_*) δ 8.32 (d, *J* = 9.0 Hz, 2H), 7.99 (d, *J* = 5.7 Hz, 1H), 7.63 (d, *J* = 8.8 Hz, 2H), 7.50 (dd, *J* = 11.0, 1.9 Hz, 1H), 7.43 (t, *J* = 8.3 Hz, 1H), 7.26 (dd, *J* = 8.3, 1.3 Hz, 1H), 7.02 (d, *J* = 4.3 Hz, 2H), 2.36 (s, 3H). LRMS (ESI) *m/z*: 460.15 [M + H]^+^.

#### 4.1.78. 3-amino-5-(3-fluoro-4-((4-methylpyridin-2-yl)oxy)phenyl)-1-(4-nitrophenyl)-1H-pyrazole-4-carbonitrile (M13-8)

**M13-8** (3.57 g, 70%, yellow solid) was synthesised from **M13-6** (5.44 g, 11.84 mmol) in a similar manner as **M1-7** and **M1-8**. ^1^H NMR (500 MHz, DMSO-*d_6_*) δ 8.24 (d, *J* = 9.1 Hz, 2H), 7.98 (d, *J* = 5.7 Hz, 1H), 7.45 (m, 4H), 7.23 (dd, *J* = 8.3, 1.4 Hz, 1H), 7.00 (d, *J* = 4.4 Hz, 2H), 2.34 (s, 3H). LRMS (ESI) *m/z*: 431.20 [M + H]^+^.

#### 4.1.79. 3-(3-fluoro-4-((4-methylpyridin-2-yl)oxy)phenyl)-2-(4-nitrophenyl)-2H-pyrazolo[3,4-d]pyrimidin-4-amine (M13-9)

**M13-9** (667 mg, 17.6%, yellow solid) was synthesised from **M13-8** (3.57 g, 8.29 mmol) in a similar manner as **M1-9**. ^1^H NMR (500 MHz, DMSO-*d_6_*) δ 8.32 (s, 1H), 8.31 (d, *J* = 3.2 Hz, 2H), 8.02 (d, *J* = 5.1 Hz, 1H), 7.67 (d, *J* = 9.0 Hz, 2H), 7.60 (dd, *J* = 10.9, 1.8 Hz, 1H), 7.42 (t, *J* = 8.3 Hz, 1H), 7.32 (dd, *J* = 8.3, 1.5 Hz, 1H), 7.03 (d, *J* = 5.2 Hz, 1H), 6.99 (s, 1H), 2.35 (s, 3H). LRMS (ESI) *m/z*: 458.15 [M + H]^+^.

#### 4.1.80. 2-(4-aminophenyl)-3-(3-fluoro-4-((4-methylpyridin-2-yl)oxy)phenyl)-2H-pyrazolo[3,4-d]pyrimidin-4-amine (M13-10)

**M13-10** (449 mg, 72%, white solid) was synthesised from **M13-9** (667 mg, 1.46 mmol) in a similar manner as **M1-10**. ^1^H NMR (500 MHz, DMSO-*d_6_*) δ 8.26 (s, 1H), 8.01 (d, *J* = 5.1 Hz, 1H), 7.43 (dd, *J* = 11.1, 1.9 Hz, 1H), 7.38 (t, *J* = 8.3 Hz, 1H), 7.23 (dd, *J* = 8.2, 1.6 Hz, 1H), 7.03–6.96 (m, 4H), 6.53 (d, *J* = 8.7 Hz, 2H), 2.35 (s, 3H). LRMS (ESI) *m/z*: 428.20 [M + H]^+^.

#### 4.1.81. N-(4-(4-amino-3-(3-fluoro-4-((4-methylpyridin-2-yl)oxy)phenyl)-2H-pyrazolo[3,4-d]pyrimidin-2-yl)phenyl)acrylamide (PLW13)

**PLW13** (80 mg, 15.8%, white solid) was synthesised from **M13-10** (449 mg, 1.05 mmol) in a similar manner as **PLW1**. **^1^H NMR** (500 MHz, DMSO-*d_6_*) δ 10.37 (s, 1H), 8.28 (s, 1H), 8.00 (d, *J* = 5.2 Hz, 1H), 7.72 (d, *J* = 8.9 Hz, 2H), 7.49 (dd, *J* = 11.0, 2.0 Hz, 1H), 7.39 (d, *J* = 8.3 Hz, 1H), 7.36 (d, *J* = 8.9 Hz, 2H), 7.25 (dd, *J* = 8.2, 1.5 Hz, 1H), 7.01 (d, *J* = 5.1 Hz, 1H), 6.96 (s, 1H), 6.43 (dd, *J* = 17.0, 10.1 Hz, 1H), 6.27 (dd, *J* = 17.0, 1.9 Hz, 1H), 5.78 (dd, *J* = 10.1, 1.9 Hz, 1H), 2.34 (s, 3H). **^13^C NMR** (126 MHz, DMSO-*d_6_*) δ 165.78, 164.51, 161.87, 161.42, 159.16, 156.26 (d, *J* = 249.8 Hz), 154.05, 149.21, 144.16 (d, *J* = 11.4 Hz), 141.71, 137.50, 136.35, 133.96, 129.85, 129.63, 129.18, 128.55, 126.97, 123.21, 121.64, 121.28 (d, *J* = 19.9 Hz), 113.24, 103.53, 22.81. ESI-HRMS *m/z*: calcd. For C_26_H_20_FN_7_O_2_ [M + H]^+^ 482.1740; found: 482.1740. HPLC: 98.5564%.

#### 4.1.82. Ethyl 3-fluoro-4-((6-methylpyridin-2-yl)oxy)benzoate (M14-2)

**M14-2** (12.6 g, 84.3%, white solid) was synthesised from **M1-1** (10 g, 54.3 mmol) in a similar manner as **M1-2**. ^1^H NMR (500 MHz, CDCl_3_) δ 7.88–7.82 (m, 2H), 7.60 (t, *J* = 7.8 Hz, 1H), 7.26 (t, *J* = 8.2 Hz, 1H), 6.90 (d, *J* = 7.3 Hz, 1H), 6.75 (d, *J* = 8.1 Hz, 1H), 4.38 (q, *J* = 7.1 Hz, 2H), 2.38 (s, 3H), 1.40 (t, *J* = 7.1 Hz, 3H). LRMS (ESI) *m/z*: 276.10 [M + H]^+^.

#### 4.1.83. 3-(3-fluoro-4-((6-methylpyridin-2-yl)oxy)phenyl)-3-oxopropanenitrile (M14-3)

**M14-3** (8.81 g, 71.2%, white solid) was synthesised from **M14-2** (12.6 g, 45.8 mmol) in a similar manner as **M1-3**. ^1^H NMR (500 MHz, CDCl_3_) δ 7.76 (dd, *J* = 10.6, 2.1 Hz, 1H), 7.73–7.69 (m, 1H), 7.64 (t, *J* = 7.8 Hz, 1H), 7.37–7.33 (m, 1H), 6.94 (d, *J* = 7.4 Hz, 1H), 6.82 (d, *J* = 8.1 Hz, 1H), 4.08 (s, 2H), 2.38 (s, 3H). LRMS (ESI) *m/z*: 271.15 [M + H]^+^.

#### 4.1.84. Ethyl 4-cyano-5-(3-fluoro-4-((6-methylpyridin-2-yl)oxy)phenyl)-1-(4-nitrophenyl)-1H-pyrazole-3-carboxylate (M14-5)

**M14-5** (8.9 g, 56%, yellow solid) was synthesised from **M14-3** (8.81 g, 32.6 mmol) and **M1-4** (10.62 g, 39.12 mmol) in a similar manner as **M1-5**. ^1^H NMR (500 MHz, CDCl_3_) δ 8.32–8.26 (m, 2H), 7.62 (dd, *J* = 10.5, 4.8 Hz, 1H), 7.60–7.54 (m, 2H), 7.33 (t, *J* = 7.9 Hz, 1H), 7.14 (dd, *J* = 17.3, 9.3 Hz, 2H), 6.93 (d, *J* = 7.2 Hz, 1H), 6.80 (d, *J* = 7.8 Hz, 1H), 4.54 (q, *J* = 6.9 Hz, 2H), 2.39 (s, 3H), 1.48 (t, *J* = 7.0 Hz, 3H). LRMS (ESI) *m/z*: 488.15 [M + H]^+^.

#### 4.1.85. 4-cyano-5-(3-fluoro-4-((6-methylpyridin-2-yl)oxy)phenyl)-1-(4-nitrophenyl)-1H-pyrazole-3-carboxylic acid (M14-6)

**M14-6** (6.79 g, 81%, yellow solid) was synthesised from **M14-5** (8.9 g, 18.25 mmol) in a similar manner as **M1-6**. ^1^H NMR (500 MHz, DMSO-*d_6_*) δ 8.32 (d, *J* = 9.1 Hz, 2H), 7.80–7.76 (m, 1H), 7.63 (d, *J* = 9.0 Hz, 2H), 7.50 (dd, *J* = 11.0, 2.0 Hz, 1H), 7.44 (t, *J* = 8.3 Hz, 1H), 7.30 (dd, *J* = 8.4, 1.5 Hz, 1H), 7.04 (d, *J* = 7.4 Hz, 1H), 6.93 (d, *J* = 8.1 Hz, 1H), 2.30 (s, 3H). LRMS (ESI) *m/z*: 460.10 [M + H]^+^.

#### 4.1.86. 3-amino-5-(3-fluoro-4-((6-methylpyridin-2-yl)oxy)phenyl)-1-(4-nitrophenyl)-1H-pyrazole-4-carbonitrile (M14-8)

**M14-8** (5.33 g, 83.9%, yellow solid) was synthesised from **M14-6** (6.79 g, 14.78 mmol) in a similar manner as **M1-7** and **M1-8**. ^1^H NMR (500 MHz, DMSO-*d_6_*) δ 8.36 (d, *J* = 10.0 Hz, 2H), 7.79 (t, *J* = 10.0 Hz, 1H), 7.69 (d, *J* = 9.1 Hz, 2H), 7.53 (dd, *J* = 11.0, 2.0 Hz, 1H), 7.48 (t, *J* = 8.2 Hz, 1H), 7.34 (dd, *J* = 8.4, 1.6 Hz, 1H), 7.05 (d, *J* = 7.4 Hz, 1H), 6.95 (d, *J* = 8.2 Hz, 1H), 2.51 (s, 3H). LRMS (ESI) *m/z*: 431.15 [M + H]^+^.

#### 4.1.87. 3-(3-fluoro-4-((6-methylpyridin-2-yl)oxy)phenyl)-2-(4-nitrophenyl)-2H-pyrazolo[3,4-d]pyrimidin-4-amine (M14-9)

**M14-9** (1.44 g, 25.3%, yellow solid) was synthesised from **M14-8** (5.33 g, 12.4 mmol) in a similar manner as **M1-9**. ^1^H NMR (500 MHz, DMSO-*d_6_*) δ 8.31–8.26 (m, 3H), 7.75 (t, *J* = 7.8 Hz, 1H), 7.65 (d, *J* = 9.0 Hz, 2H), 7.58 (dd, *J* = 10.9, 1.8 Hz, 1H), 7.40 (t, *J* = 8.2 Hz, 1H), 7.31 (dd, *J* = 8.3, 1.5 Hz, 1H), 7.02 (d, *J* = 7.3 Hz, 1H), 6.91 (d, *J* = 8.1 Hz, 1H), 2.30 (s, 3H). LRMS (ESI) *m/z*: 458.15 [M + H]^+^.

#### 4.1.88. 2-(4-aminophenyl)-3-(3-fluoro-4-((6-methylpyridin-2-yl)oxy)phenyl)-2H-pyrazolo[3,4-d]pyrimidin-4-amine (M14-10)

**M14-10** (1.15 g, 86%, white solid) was synthesised from **M14-9** (1.44 g, 3.14 mmol) in a similar manner as **M1-10**. ^1^H NMR (500 MHz, DMSO-*d_6_*) δ 8.23 (s, 1H), 7.74 (t, *J* = 7.8 Hz, 1H), 7.42 (dd, *J* = 11.0, 1.9 Hz, 1H), 7.35 (t, *J* = 8.3 Hz, 1H), 7.21 (dd, *J* = 8.3, 1.4 Hz, 1H), 7.02 (d, *J* = 7.3 Hz, 1H), 6.98 (d, *J* = 8.7 Hz, 2H), 6.88 (d, *J* = 8.2 Hz, 1H), 6.50 (d, *J* = 8.7 Hz, 2H), 2.30 (s, 3H). LRMS (ESI) *m/z*: 428.20 [M + H]^+^.

#### 4.1.89. N-(4-(4-amino-3-(3-fluoro-4-((6-methylpyridin-2-yl)oxy)phenyl)-2H-pyrazolo[3,4-d]pyrimidin-2-yl)phenyl)acrylamide (PLW14):

**PLW14** (49 mg, 8.8%, white solid) was synthesised from **M14-10** (500 mg, 1.17 mmol) in a similar manner as **PLW1**. **^1^H NMR** (500 MHz, DMSO-*d_6_*) δ 10.40 (s, 1H), 8.28 (s, 1H), 7.76 (d, *J* = 7.6 Hz, 1H), 7.73 (d, *J* = 8.9 Hz, 2H), 7.49 (dd, *J* = 11.0, 2.0 Hz, 1H), 7.38 (d, *J* = 8.3 Hz, 1H), 7.35 (d, *J* = 9.0 Hz, 2H), 7.25 (dd, *J* = 8.3, 1.5 Hz, 1H), 7.03 (d, *J* = 7.4 Hz, 1H), 6.90 (d, *J* = 8.2 Hz, 1H), 6.44 (dd, *J* = 17.0, 10.2 Hz, 1H), 6.27 (dd, *J* = 17.0, 1.9 Hz, 1H), 5.78 (dd, *J* = 10.1, 1.9 Hz, 1H), 2.30 (s, 3H). **^13^C NMR** (126 MHz, DMSO-*d_6_*) δ 165.79, 163.75, 161.90, 161.45, 159.21, 158.94, 156.35 (d, *J* = 247.9 Hz), 144.30 (d, *J* = 11.7 Hz), 142.96, 141.72, 137.54, 136.33, 134.00, 129.81, 129.68, 129.08, 128.59, 126.92, 121.57, 121.30 (d, *J* = 20.2 Hz), 121.05, 109.73, 103.53, 25.97. ESI-HRMS *m/z*: calcd. For C_26_H_20_FN_7_O_2_ [M + H]^+^ 482.1735; found: 482.1738. HPLC: 97.2938%.

#### 4.1.90. N-(4-(4-amino-3-(3-fluoro-4-((6-methylpyridin-2-yl)oxy)phenyl)-2H-pyrazolo[3,4-d]pyrimidin-2-yl)phenyl)propionamide (PLW14N):

**PLW14N** (42 mg, 17%, white solid) was synthesised from **M14-10** (500 mg, 1.17 mmol) in a similar manner as **PLW1**. **^1^H NMR** (500 MHz, DMSO-*d_6_*) δ 10.18 (s, 1H), 8.63 (s, 1H), 7.77 (t, *J* = 7.7 Hz, 1H), 7.69 (d, *J* = 8.7 Hz, 2H), 7.56 (d, *J* = 10.7 Hz, 1H), 7.40 (t, *J* = 8.2 Hz, 1H), 7.32 (d, *J* = 8.7 Hz, 2H), 7.25 (d, *J* = 8.3 Hz, 1H), 7.04 (d, *J* = 7.3 Hz, 1H), 6.90 (d, *J* = 8.1 Hz, 1H), 2.34 (dd, *J* = 15.0, 7.5 Hz, 2H), 2.31 (s, 3H), 1.07 (t, *J* = 7.5 Hz, 3H). **^13^C NMR** (126 MHz, DMSO-*d_6_*) δ 172.92, 161.69, 159.25, 158.98, 157.07, 154.58 (d, *J* = 248.1 Hz), 143.19 (d, *J* = 11.8 Hz), 141.07, 140.87, 140.16, 132.71, 127.77, 127.15, 125.13, 124.20, 124.13, 119.53, 119.36, 119.28, 107.90, 99.97, 30.04, 24.00, 10.00. ESI-HRMS *m/z*: calcd. For C_26_H_22_FN_7_O_2_ [M + H]^+^ 484.1897; found: 484.1897. HPLC: 97.0909%.

### 4.2. General methods for biology

#### 4.2.1. Cell culture

A204, SNU-16, BaF3, BaF3-EVT6-FGFR1 and BaF3-EVT6-FGFR2 were kindly provided by Jinan University. All cell lines were cultured for less than 2 months after thaw and used within 20 passages. Cell lines were cultured at 37 °C in 5% CO_2_ humidified air in media recommended by the ATCC.

#### 4.2.2. Western blot analysis (WB)

A204 and SNU-16 cells were treated with different concentrations (0.1, 0.3, 1, 3, 10, 30, 100, 300 nM) of our best candidate **PLW559** or DMSO (D8418-1L, CAS-No:67*-*87-5, Sigma-Aldrich) for 1 h. Then, cells were lysed with 1 × lysis buffer. The cell lysates were loaded and then electrophoresed to 10% SDS‐PAGE gel, and the separated proteins were transferred to PVDF membranes. After blocking with 5% milk in TBS at room temperature for 2 h, the membranes were incubated with the corresponding primary antibody (1:1000) at 4 °C overnight. After washing with TBST for 3 times, the HRP‐conjugated secondary antibodies were incubated for 2 h. The protein signals were detected with Amersham Imager 800 system using a Western Lightning Plus-ECL (Perkin Elmer). Primary antibodies FGFR2(23328S), phospho‐FGFR (3471S), phospho‐FRS2α (3861S), ERK1/2 (4695S), phospho‐ERK1/2 (4370S), Akt (pan) (11E7) (4685S), phospho‐AKT (9271S), GAPDH (5174), and secondary antibodies (1:2000) were purchased from Cell Signalling Technology (Boston, MA, USA).

#### 4.2.3. Cell proliferation assay

Cells were seeded in 384-well plate in complete medium (2000–4000 cells/well) and cultured overnight. Then the cells were exposed to gradient concentrations of compounds. Cell proliferation was analysed by Cell Counting Kit 8 (C0037, Beyotime) after co-incubation for a further 72 h. IC_50_ values were calculated by fitting of concentration–response curves using GraphPad Prism 8.01 software. Each assay was repeated at least three times. Each IC_50_ value is displayed as mean ± SD of at least two independent experiments.

#### 4.2.4. Apoptosis assay

A204 and SNU-16 cells were treated with indicated concentrations of our best candidate **PLW559** or DMSO (D8418-1L, CAS-No:67*-*87-5, Sigma-Aldrich) for 48 h at 37 °C. After incubation, cells were collected and washed twice with ice‐cold PBS. About 6 × 105 cells were resuspended in 100 μl of 1× BD binding buffer solution, and then stained with 7‐AAD and Annexin V‐PE in the dark for 15 min. Finally, 400 μl of 1 × BD binding buffer solution were added to stop staining. The cells were then measured on a BD FACSCelestaTM flow cytometer.

#### 4.2.5. Purification of FGFR2 WT kinase domains

The FGFR2 kinase domain (aa P458-E768) was cloned into pSJ2 vector (derived from the modified pET28a vector, and the pET28a was purchased from Shanghai Branch of Beijing Tsingke Biotechnology Co., Ltd) with an *N*-terminal hexahistidine tag (His6) followed by the TEV sequence (MGSHHHHHHHHGSDYDIPTTENLYFQG). BL21 (DE3) (Beyotime, D131M) Escherichia coli cells were transfected with this plasmid and grown in Luria–Bertani (LB) (1 L: 10 g tryptone (OXOID,LOT:4318083), 5 g YEAST EXTRACT (OXOID, LOT:3254297), 10 g NaCl (Shanghai Dahe Chemicals, 250630)) media supplemented with 100 μg/mL ampicillin (MESGEN, Cat.No.MG1378) at 37 °C. At OD600 = 0.6, the temperature was lowered to 18 °C. Isopropyl-β-d-thiogalactopyranoside (MESGEN, Cat.No.MG1160) was added to a final concentration of 0.4 mmol/L. After 18 h, cells were harvested by centrifuge and resuspend in Tris 25 mM, NaCl 300 mM, pH 8.0 (with protease inhibitor). Cells were lysed by pressure and supernatant harvested by centrifugation. His-tagged FGFR2 kinase domain protein was captured on HisTrap HP column and eluted in Tris 25 mM, NaCl 300 mM, Imidazole 250 mM, pH 8.0. Eluted protein was incubated overnight at 4 °C with TEV protease. Protein tag was removed prior to loading onto a Superdex 75 columns. Purified protein was stored in Tris 25 mM, NaCl 150 mM, DTT 2 mM. The theoretical molecular weight of the WT FGFR2 kinase domain is 35.58 kDa. Molecular weights were confirmed experimentally using LC/MS (FGFR2 = 35961.85 Da, 36121.8 Da, 36201.76 Da and 36281.67 Da).

#### 4.2.6. FGFR kinase activity inhibition assay

**PLW559** was preincubated with FGFR1, FGFR2, FGFR3 or FGFR4 kinase (Carna Biosciences) and substrate peptide (Peptide30, GL) in 50 mmol/L HEPES, pH 7.5, 10 mmol/L MgCl_2_, 1 mmol/L EGTA, 0.01% Brij-35, 2 mmol/L DTT, and 0.05% BSA for 30 min at room temperature. Compounds were transferred 200 nl of each well from the 384-well Echo plate to a 384-well assay plate in duplicates by Echo. ATP was added to a total concentration of 100 μmol/L and incubated for a period of time, and reactions were stopped using 100 mmol/L HEPES, pH 7.5, 50 mmol/L EDTA, 0.015% Brij-35 and 0.2% coating reagent #3 (PerkinElmer). The substrate conversion ratio was calculated for each PLW559 concentration and was then used to calculate IC_50_ values.

#### 4.2.7. KINOMEscan kinase profiling of PLW559

We use the KINOMEscan screening platform (ICE Bioscience Inc., China) to analyse PLW559, which was applied to a set of 76 tyrosine kinases.

#### 4.2.8. Computational modeling studies

The structure of FGFR2 (PDB ID: 8STG) was prepared using Protein Preparation Wizard and the compounds were prepared by LigPrep. The covalent docking was carried out using covalent docking module with default settings (Schrödinger, LLC, New York, NY, 2021).

## Supplementary Material

SI without authors.docx

## Data Availability

The authors confirm that the data supporting the findings of this study are available within the article and its supplementary materials.

## Abbreviations

FGFR2: fibroblast growth factor receptor 2
WB: western blot
SAR: structure–activity relationships
DPPA: diphenylphosphoryl azide
ATP: adenosine triphosphate
DIPEA: *N,N*-diisopropylethylamine
DMF: *N,N*-dimethylformamide
DMSO: dimethyl sulfoxide
HPLC: high-performance liquid chromatography
HRMS: high-resolution mass
IC_50_: half-maximal inhibition concentration
LC–MS: liquid chromatography-mass spectrometry
PBS: phosphate-buffered saline
TFA: trifluoroacetic acid
THF: tetrahydrofuran
NMP: *N*-methyl-2-pyrrolidone
NIS: *N*-iodosuccinimide
NBS: *N*-bromosuccinimide
*i*-PrOH: isopropyl alcohol
10% SDS-PAGE: sodium dodecyl sulphate-polyacrylamide gel electrophoresis
PVDF: polyvinylidene fluoride
TBST: Tris-buffered saline with Tween
HRP: horseradish peroxidase
FRS2α: fibroblast growth factor (FGF) receptor substrate 2α
ERK1/2: extracellular-regulated kinase ½
GAPDH: glyceraldehyde-3-phosphate dehydrogenase
AKT: protein kinase B (PKB)
WT: wild type
TEV: tobacco etch virus
DTT: dithiothreitol
Boc: tert-butyloxycarbonyl
HEPES: 4-hydroxyethyl piperazine ethanesulfonic acid
EGTA: ethylene glycol tetraacetic acid
BSA: bovine serum albumin
EDTA: ethylene diamine tetraacetic acid
