# Supplementary material for: 2H-pyrazolo[3,4-d]pyrimidin-4-amine derivatives as novel selective fibroblast growth factor receptor 2 (FGFR2) inhibitors
Source: J Enzyme Inhib Med Chem. 2026 Mar 30;41(1):2647526. doi: 10.1080/14756366.2026.2647526 (PMC13037149; doi:10.1080/14756366.2026.2647526)

**Supporting Information**

2*H*-Pyrazolo[3,4-*d*]pyrimidin-4-amine Derivatives as Novel Selective Fibroblast Growth Factor Receptor 2 (FGFR2) Inhibitors

**Table of Contents**

**1.** Table S1………………………………................................................................... S3

**2.** ^1^H and ^13^C NMR spectra for all inhibitors…........................................................... S6

**3.** HPLC spectra of all inhibitors............................................................................... S21

**Table S1: Kinase selectivity profiling of WPL559**

|  | Kinase | WPL559 (%inhibition)@1μM |
| --- | --- | --- |
| **1** | FGFR2 | 96.14 |
| **2** | FGFR3 | 63.22 |
| **3** | FGFR1 | 46.98 |
| **4** | HER4 | 17.56 |
| **5** | EPHA7 | 16.96 |
| **6** | FGR | 16.02 |
| **7** | LYNa | 14.60 |
| **8** | PDGFRβ | 14.49 |
| **9** | EPHB3 | 12.19 |
| **10** | HER2 | 12.07 |
| **11** | FRK | 12.03 |
| **12** | FER | 11.82 |
| **13** | EPHA1 | 10.37 |
| **14** | RON | 10.29 |
| **15** | FLT3 | 9.47 |
| **16** | MUSK | 9.32 |
| **17** | IRR | 7.92 |
| **18** | FLT1 | 7.86 |
| **19** | LCK | 7.34 |
| **20** | ACK | 7.03 |
| **21** | KDR | 6.32 |
| **22** | TIE2 | 6.20 |
| **23** | ZAP70 | 6.11 |
| **24** | FLT4 | 5.59 |
| **25** | LYNb | 4.73 |
| **26** | ITK | 4.43 |
| **27** | TRKA | 4.26 |
| **28** | PYK2 | 3.80 |
| **29** | ABL2 | 3.43 |
| **30** | ABL1 | 3.36 |
| **31** | EGFR | 3.04 |
| **32** | SYK | 2.92 |
| **33** | AXL | 2.89 |
| **34** | MER | 2.48 |
| **35** | SRC | 1.96 |
| **36** | TEC | 0.87 |
| **37** | INSR | 0.37 |
| **38** | FYN [isoform a] | 0.24 |
| **39** | HCK | 0.13 |
| **40** | FGFR4 | -0.03 |
| **41** | EPHA4 | -0.09 |
| **42** | IGF1R | -0.26 |
| **43** | JAK3 | -0.29 |
| **44** | JAK1 | -0.31 |
| **45** | EPHA2 | -0.56 |
| **46** | FES | -0.90 |
| **47** | PDGFRα | -0.93 |
| **48** | CSK | -1.08 |
| **49** | FAK | -1.69 |
| **50** | MET | -1.73 |
| **51** | CSF1R | -1.83 |
| **52** | TRKC | -2.03 |
| **53** | RET | -2.08 |
| **54** | BTK | -2.24 |
| **55** | ROS | -3.63 |
| **56** | YES | -3.65 |
| **57** | EPHB2 | -3.82 |
| **58** | EPHA5 | -3.87 |
| **59** | EPHB1 | -4.50 |
| **60** | EPHA8 | -4.84 |
| **61** | FYN [isoform b] | -5.00 |
| **62** | TYRO3 | -5.00 |
| **63** | SRM | -5.39 |
| **64** | TYK2 | -5.57 |
| **65** | DDR2 | -7.25 |
| **66** | DDR1 | -7.64 |
| **67** | TXK | -8.10 |
| **68** | ALK | -8.13 |
| **69** | JAK2 | -8.86 |
| **70** | TRKB | -9.32 |
| **71** | EPHB4 | -11.91 |
| **72** | BMX | -13.31 |
| **73** | EPHA6 | -13.71 |
| **74** | BRK | -13.74 |
| **75** | EPHA3 | -15.11 |
| **76** | KIT | -15.13 |

**^1^H and ^13^C NMR spectra for all inhibitors**

^1^H NMR and ^13^C NMR spectra for **PLW1**


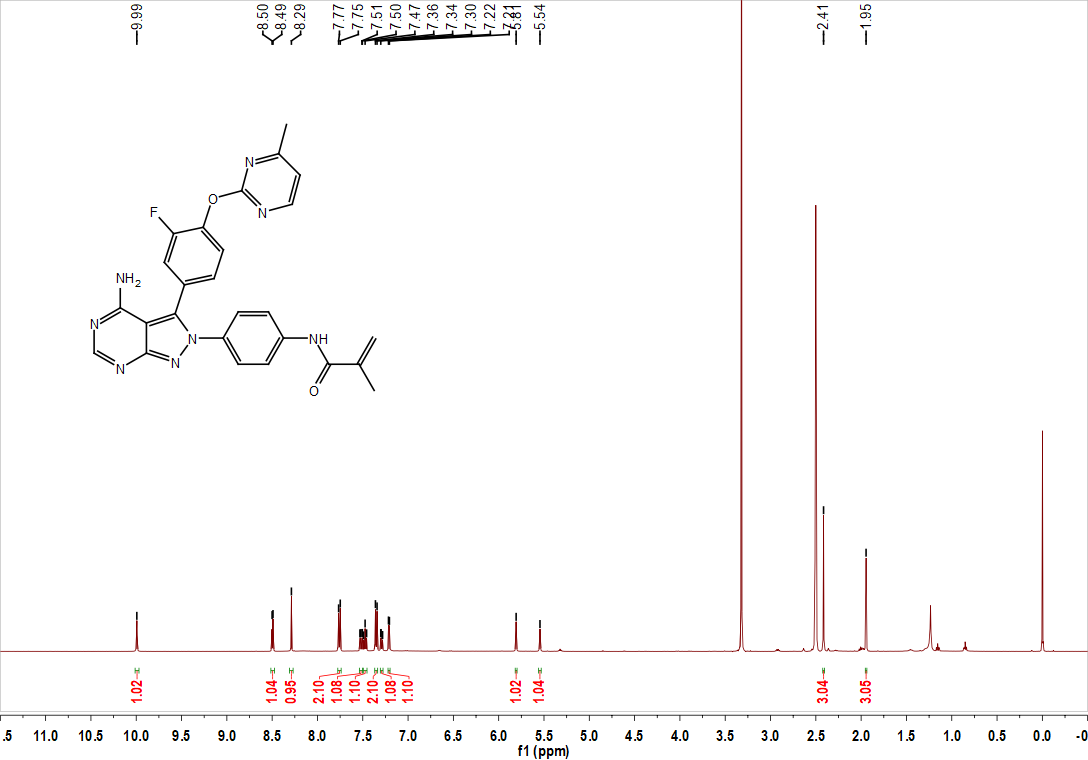


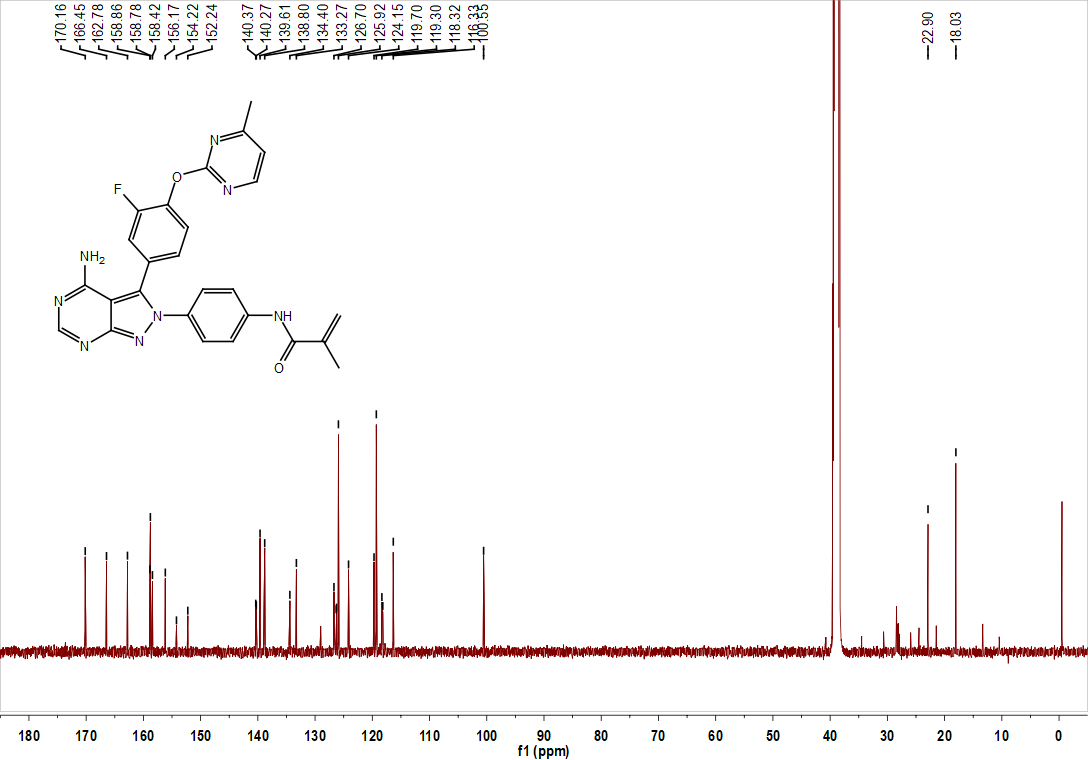


^1^H NMR and ^13^C NMR spectra for **PLW2**


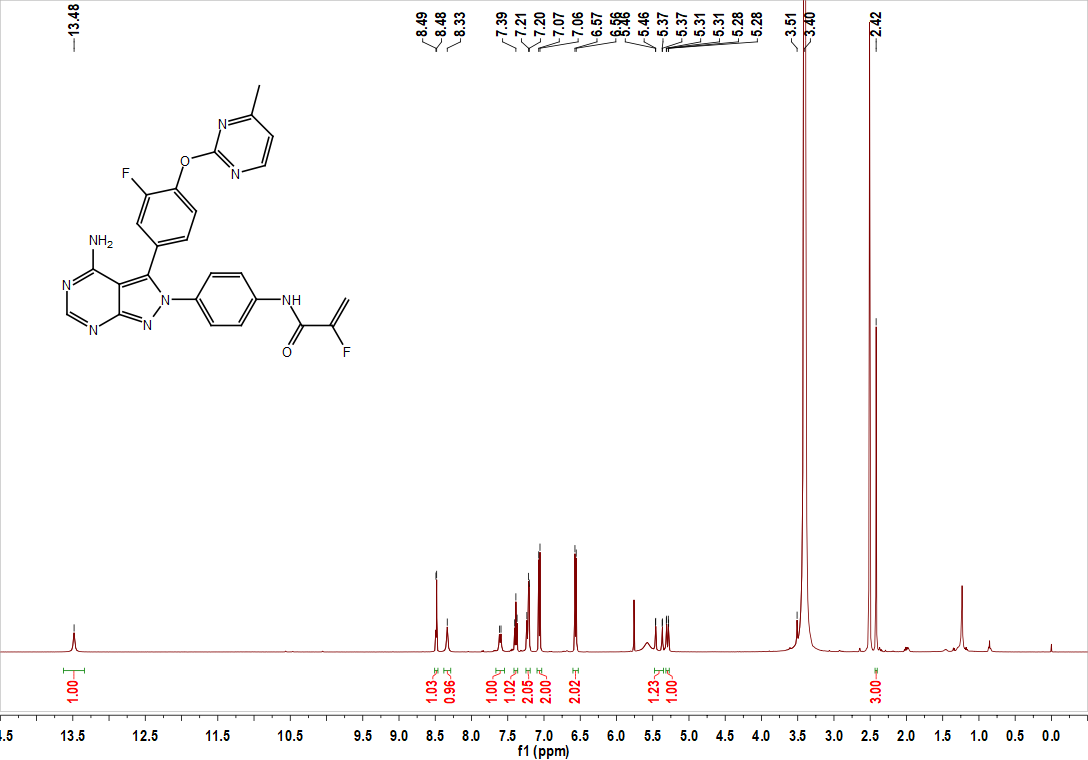


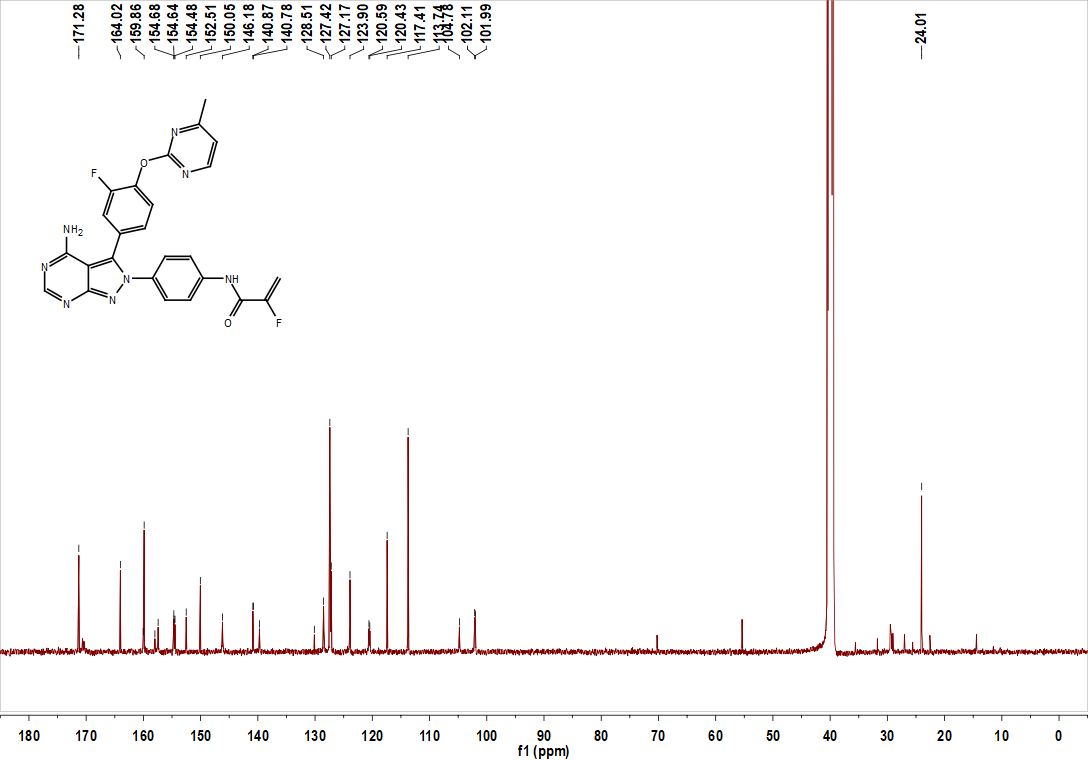


^1^H NMR and ^13^C NMR spectra for **PLW3**


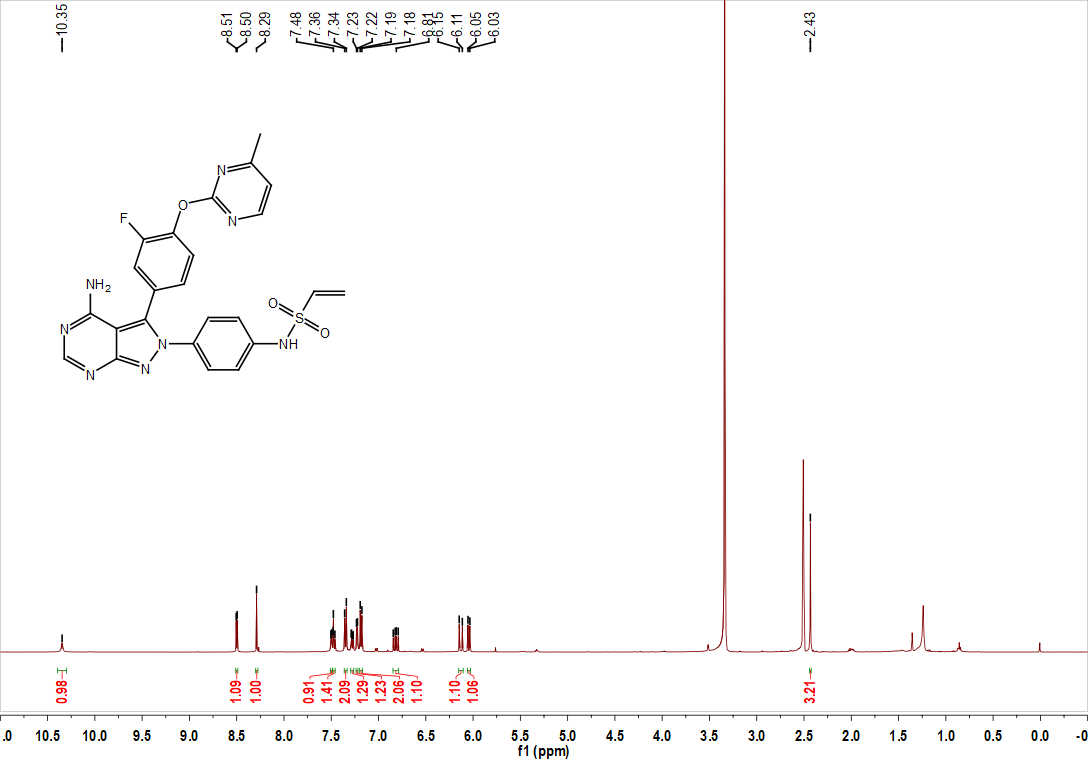


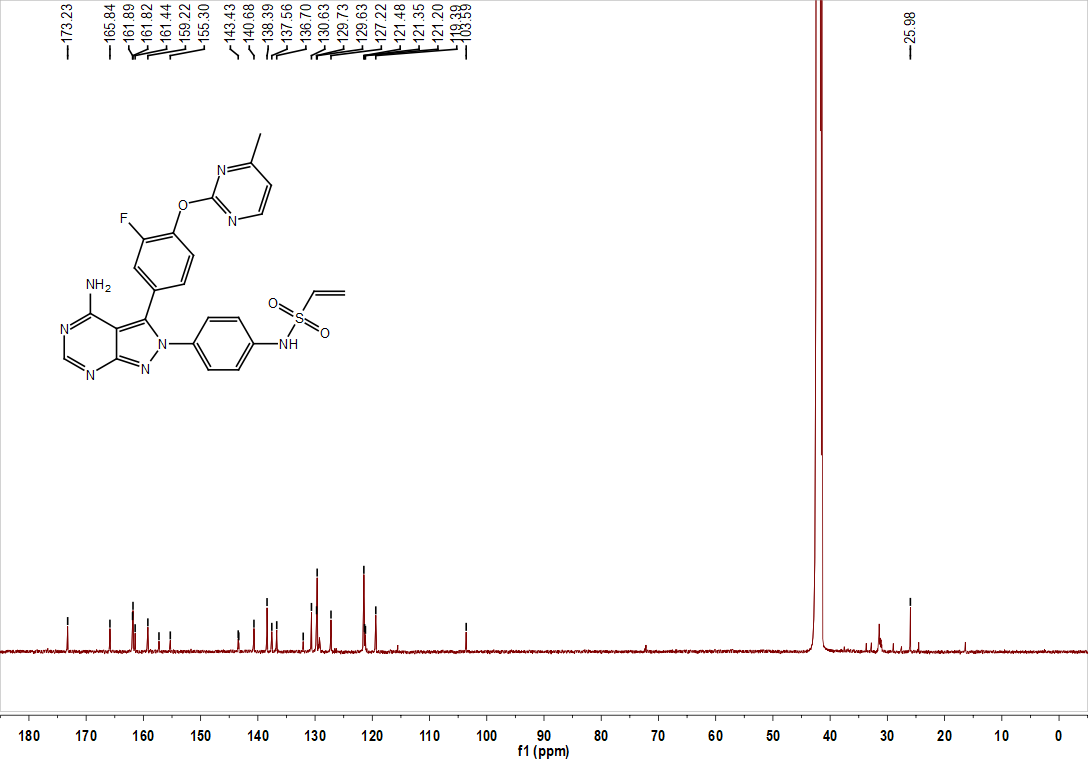


^1^H NMR and ^13^C NMR spectra for **PLW4**


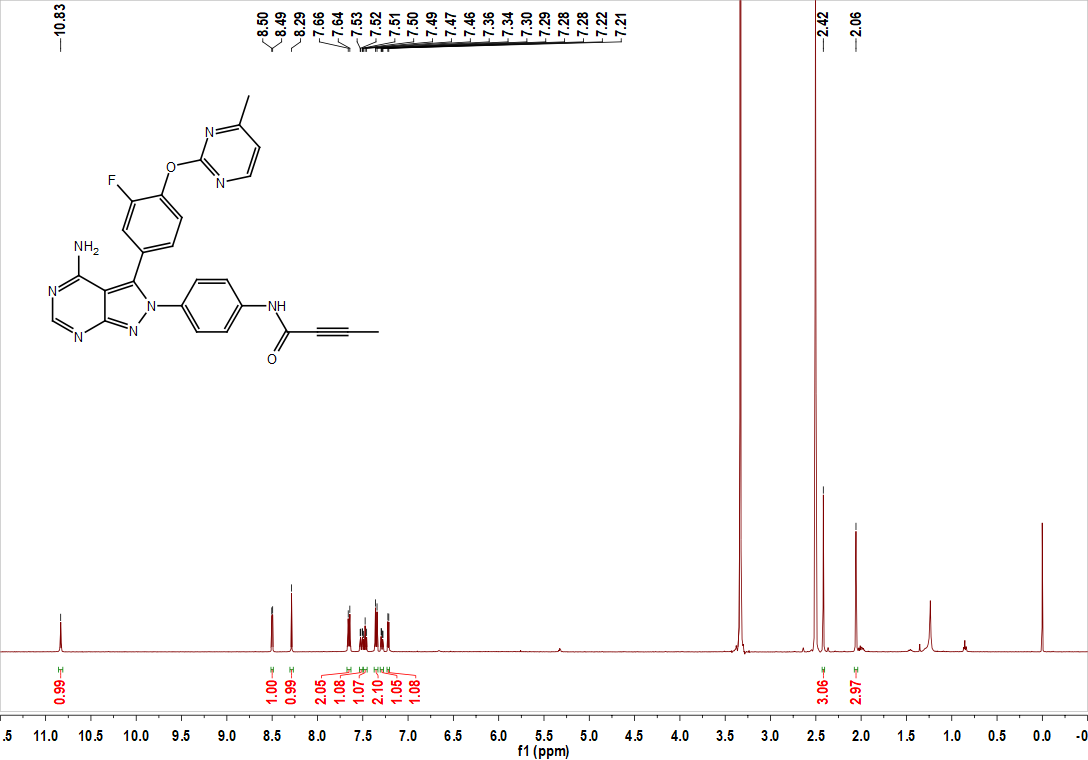


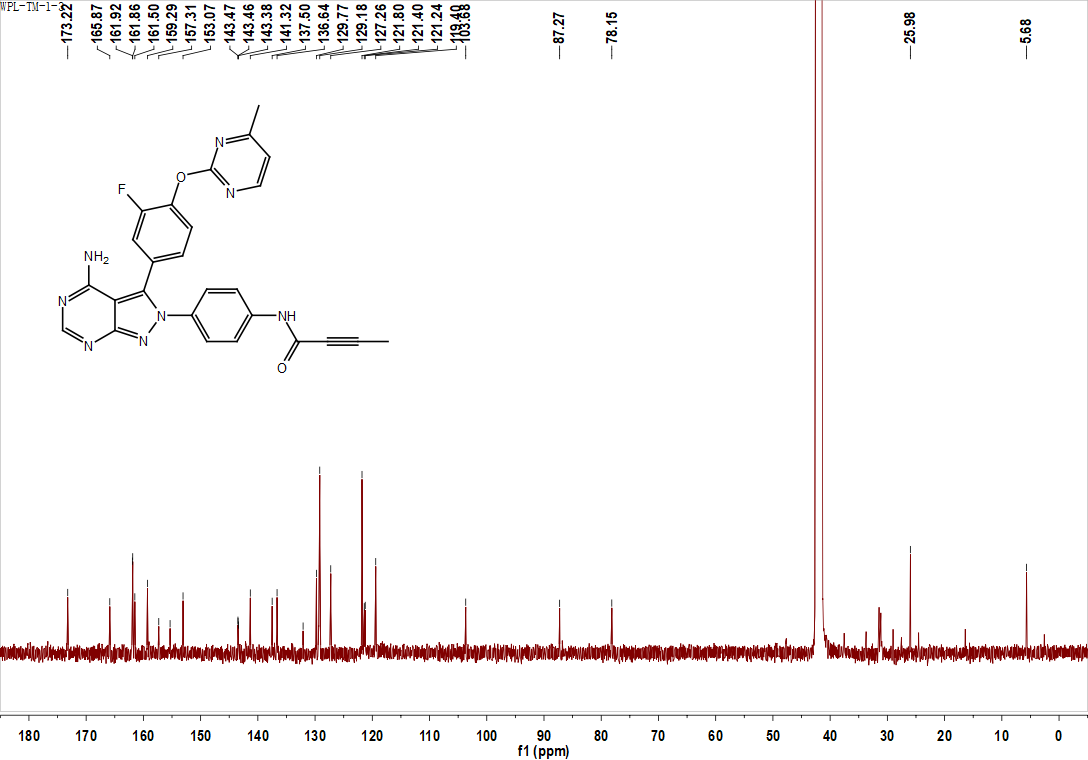


^1^H NMR and ^13^C NMR spectra for **PLW5**


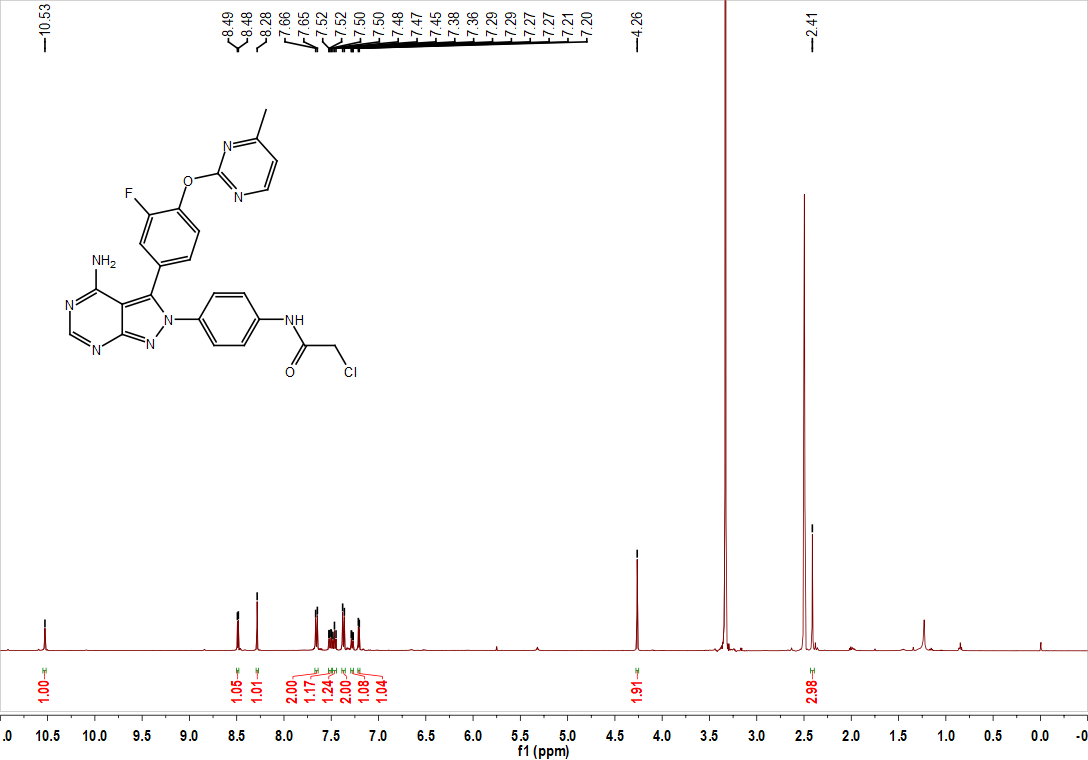


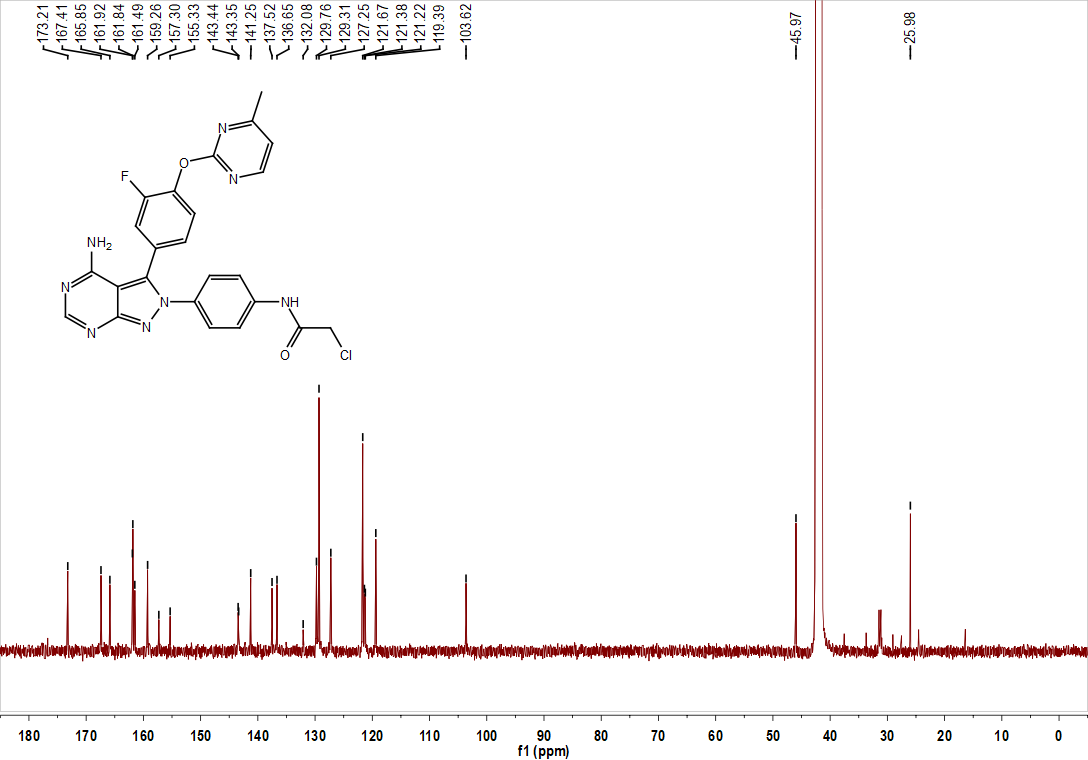


^1^H NMR and ^13^C NMR spectra for **PLW6**


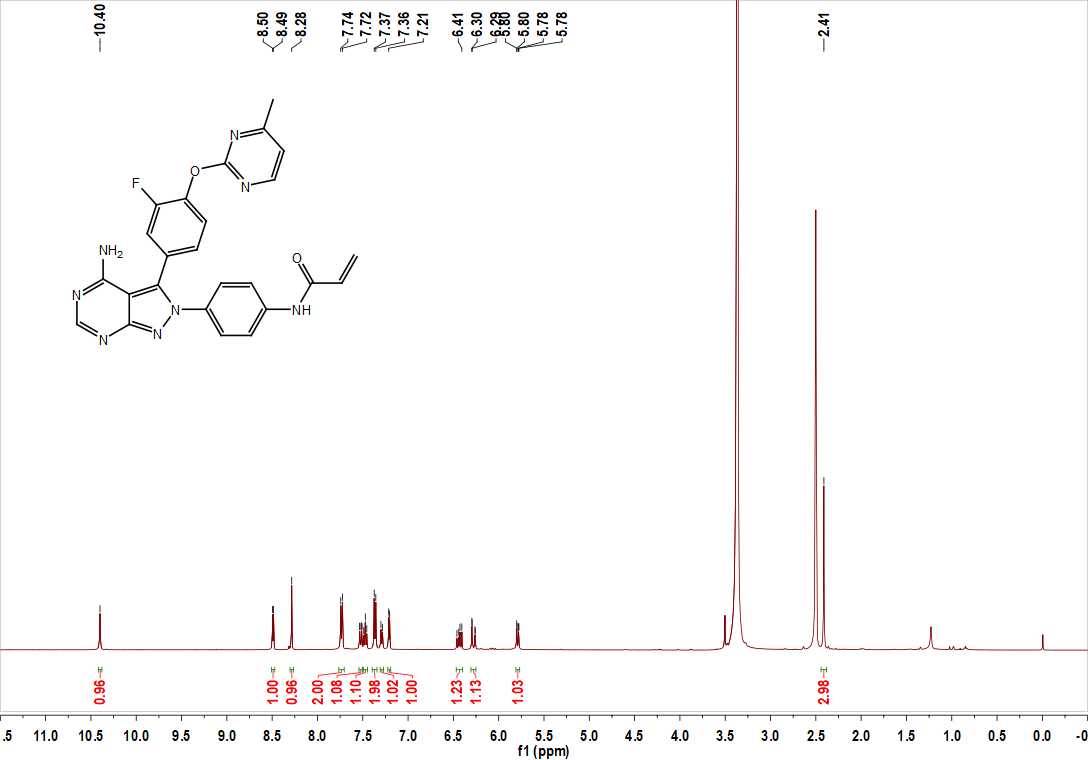


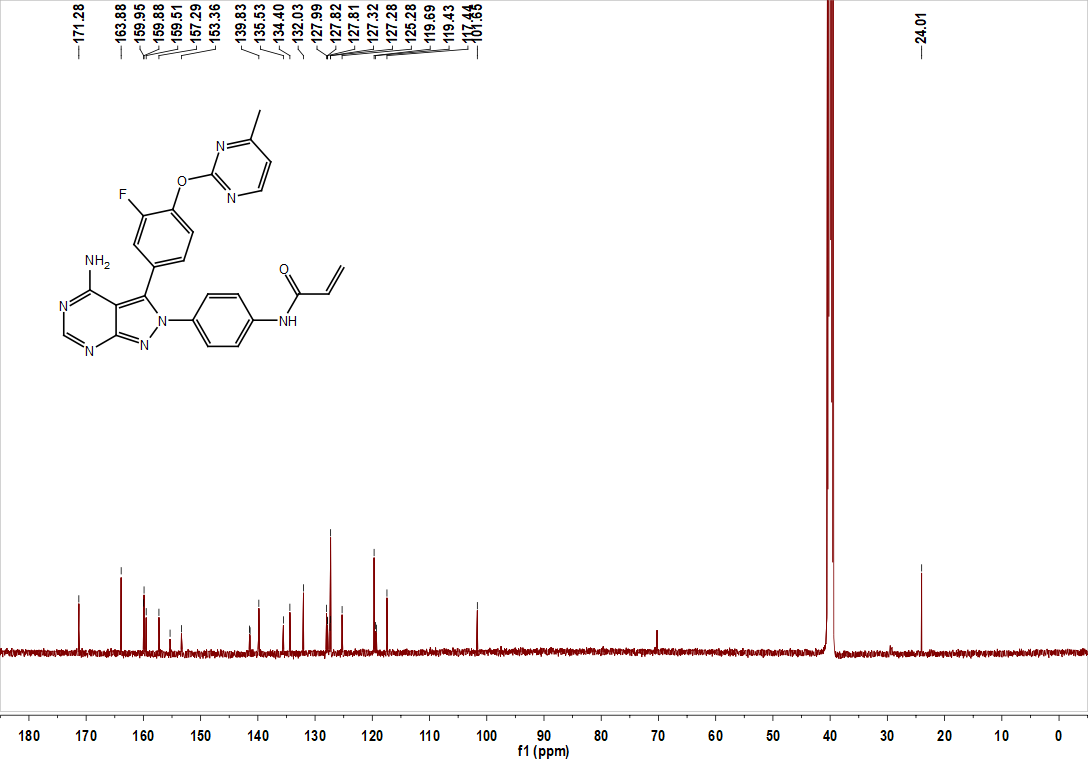


^1^H NMR and ^13^C NMR spectra for **PLW7**


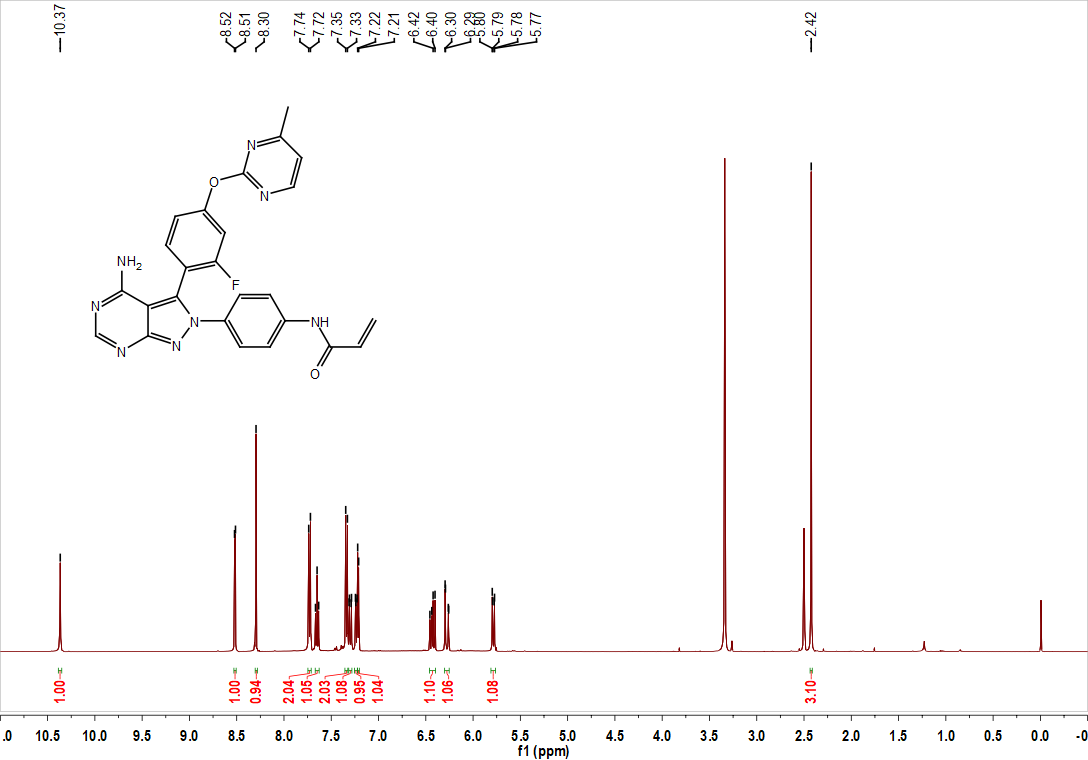


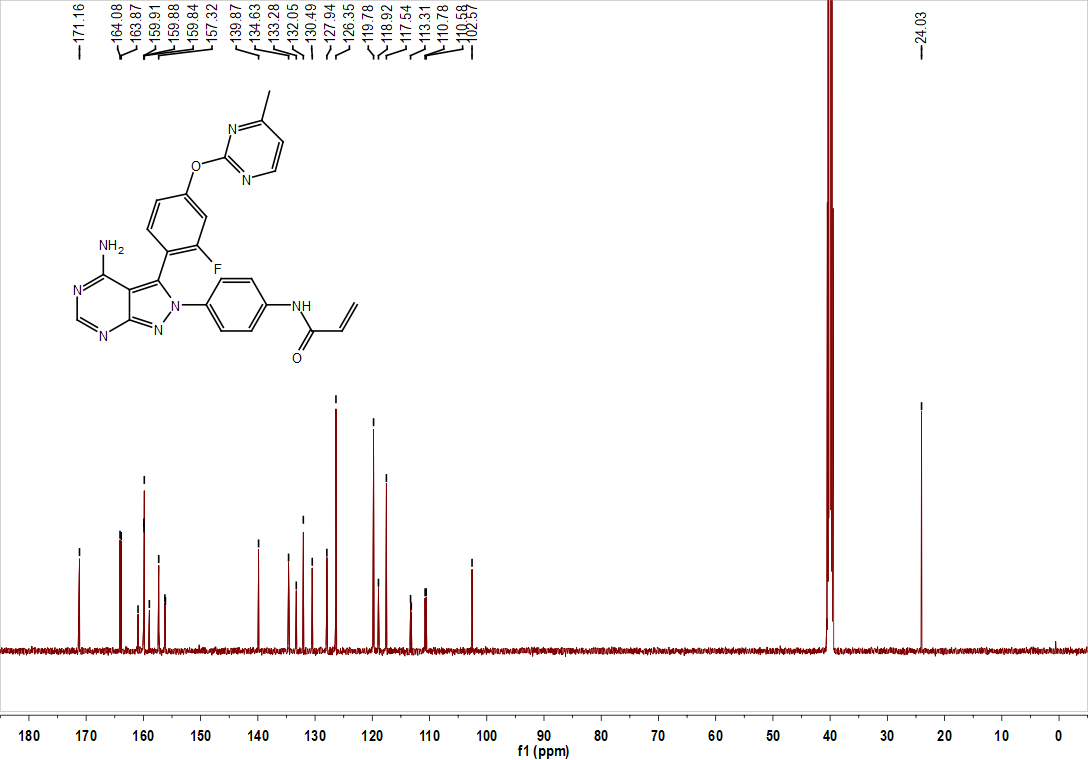


^1^H NMR and ^13^C NMR spectra for **PLW8**


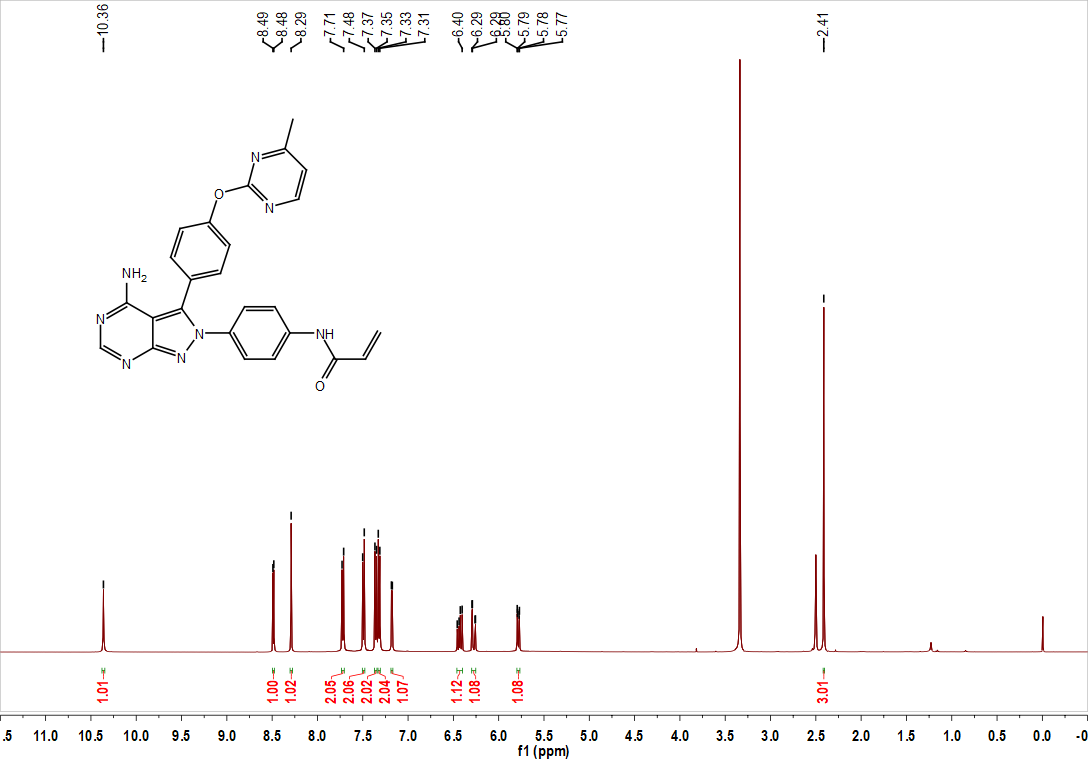


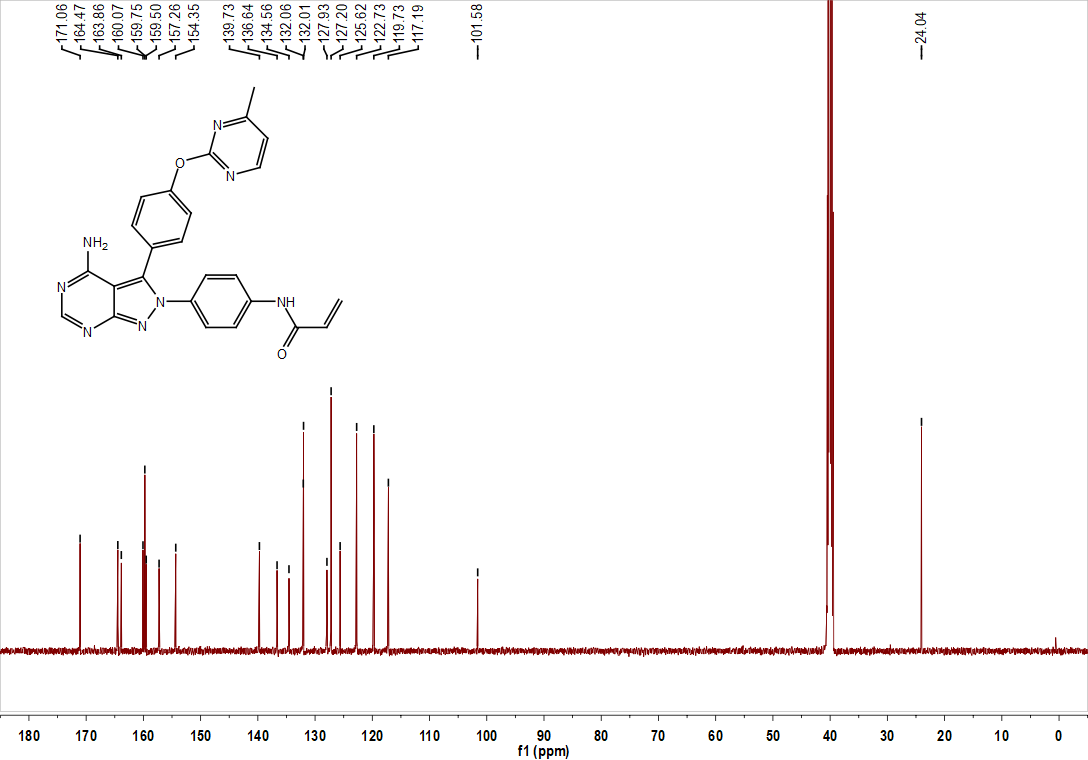


^1^H NMR and ^13^C NMR spectra for **PLW9**


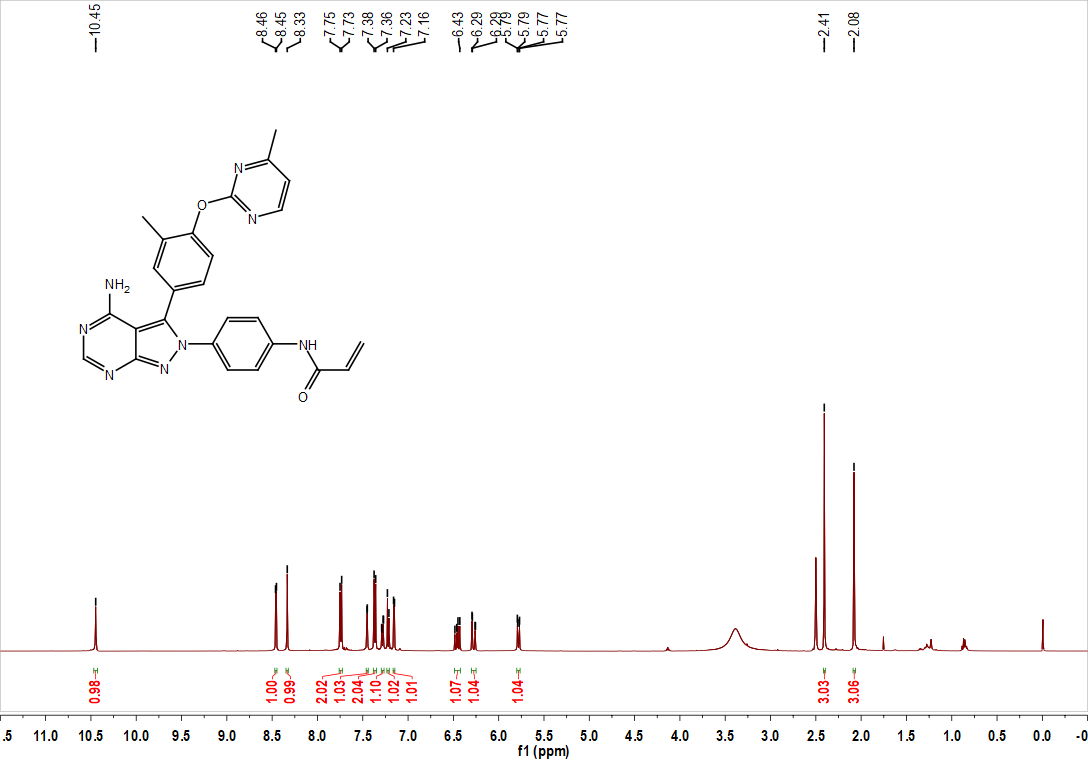


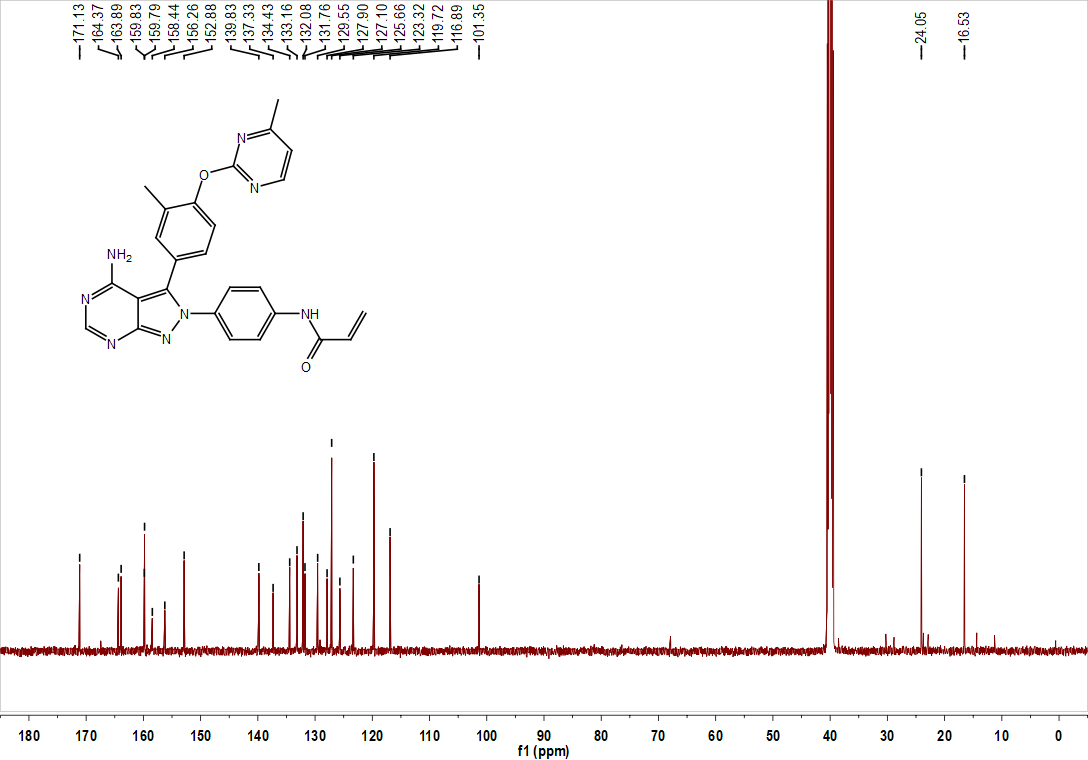


^1^H NMR and ^13^C NMR spectra for **PLW10**


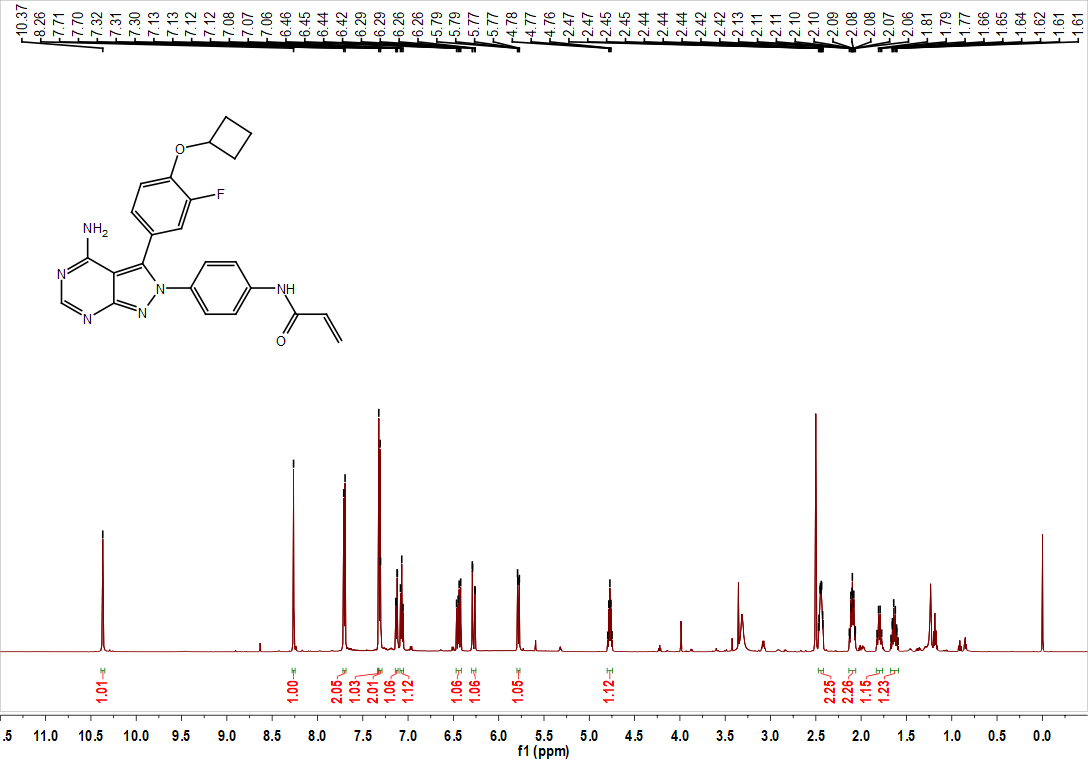


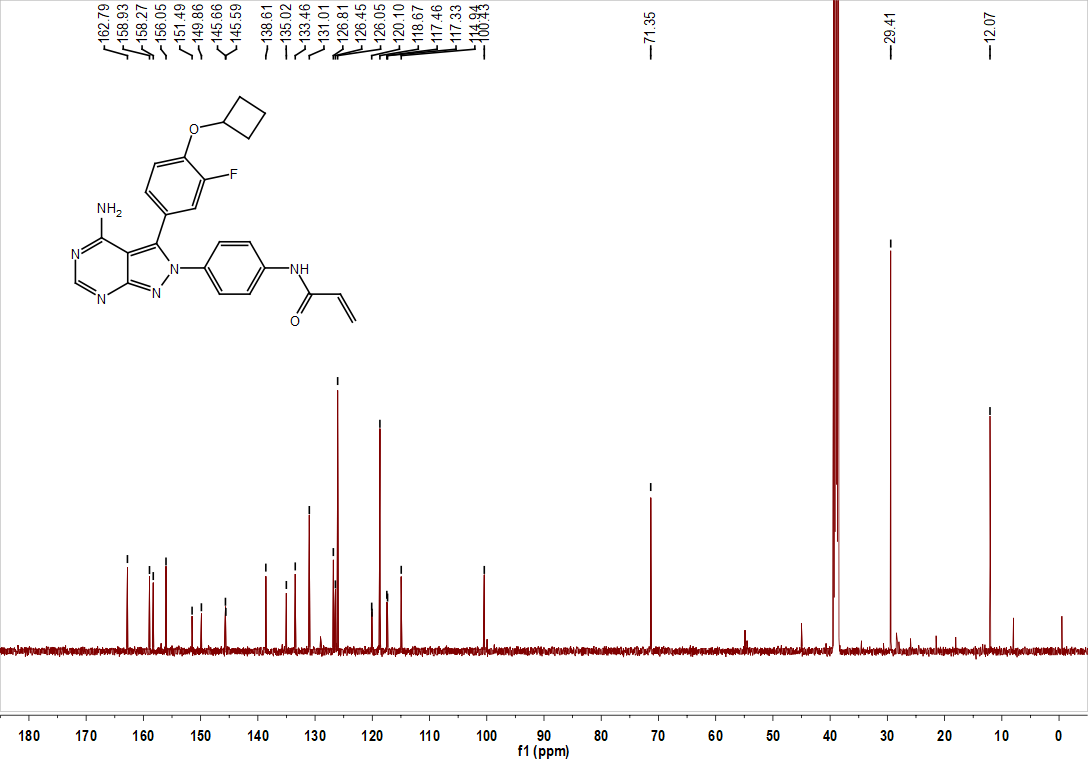


^1^H NMR and ^13^C NMR spectra for **PLW11**


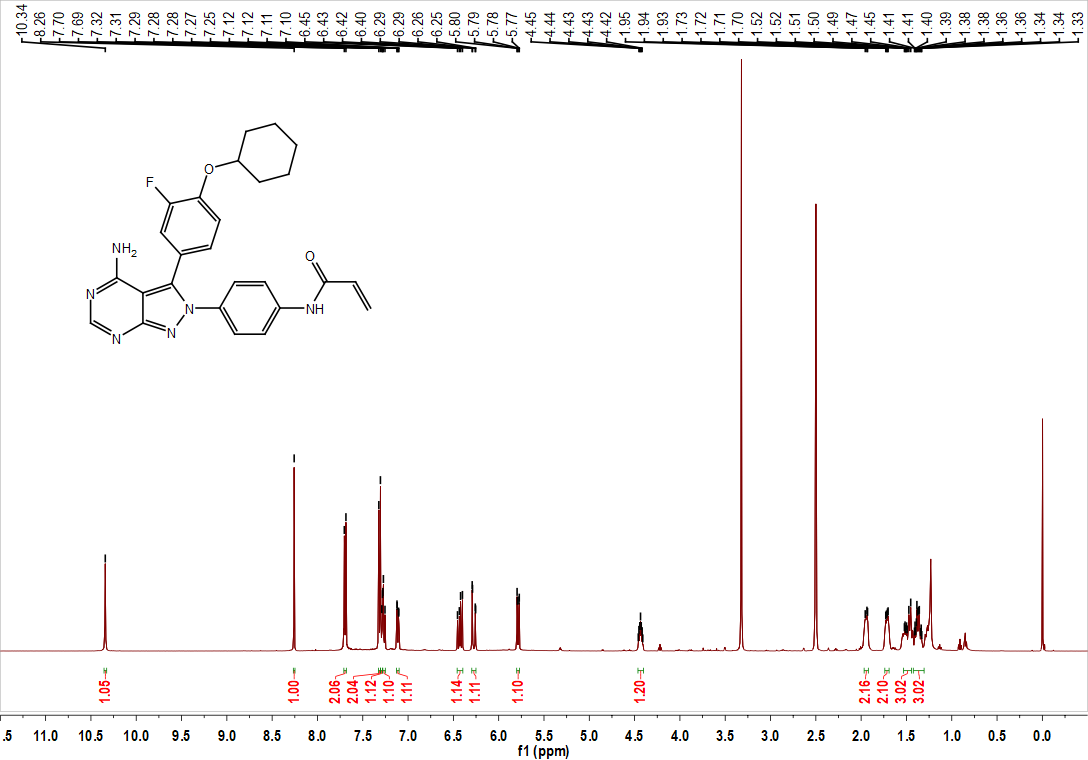


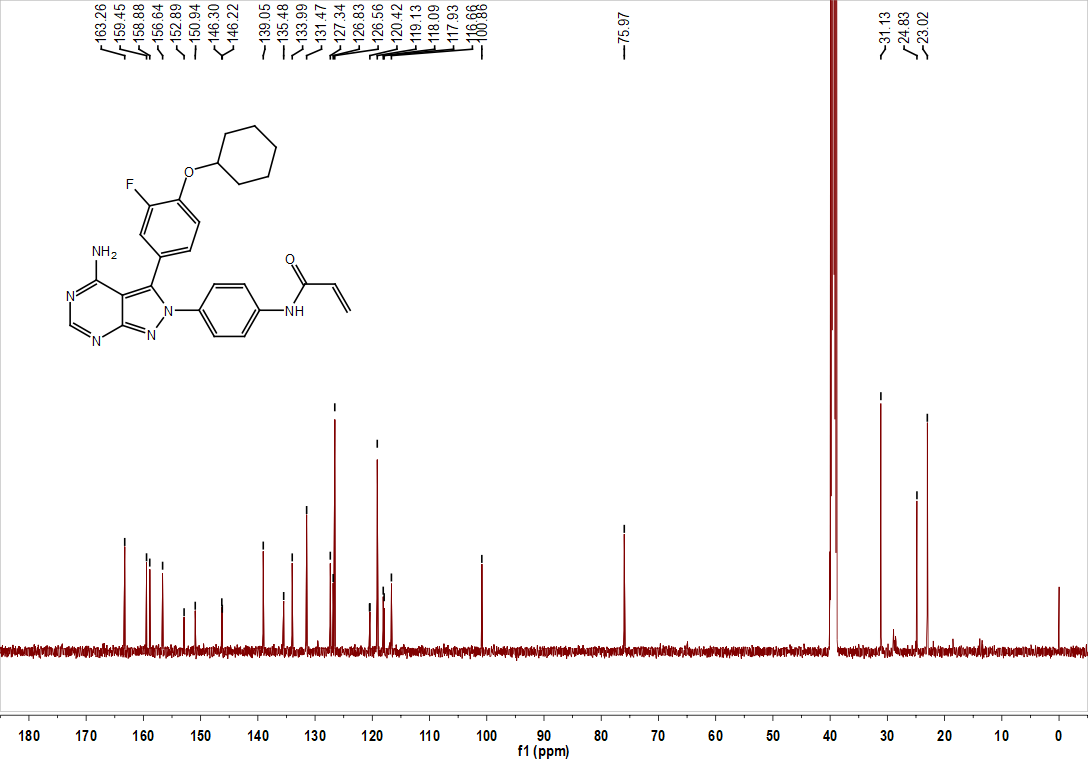


^1^H NMR and ^13^C NMR spectra for **PLW12**


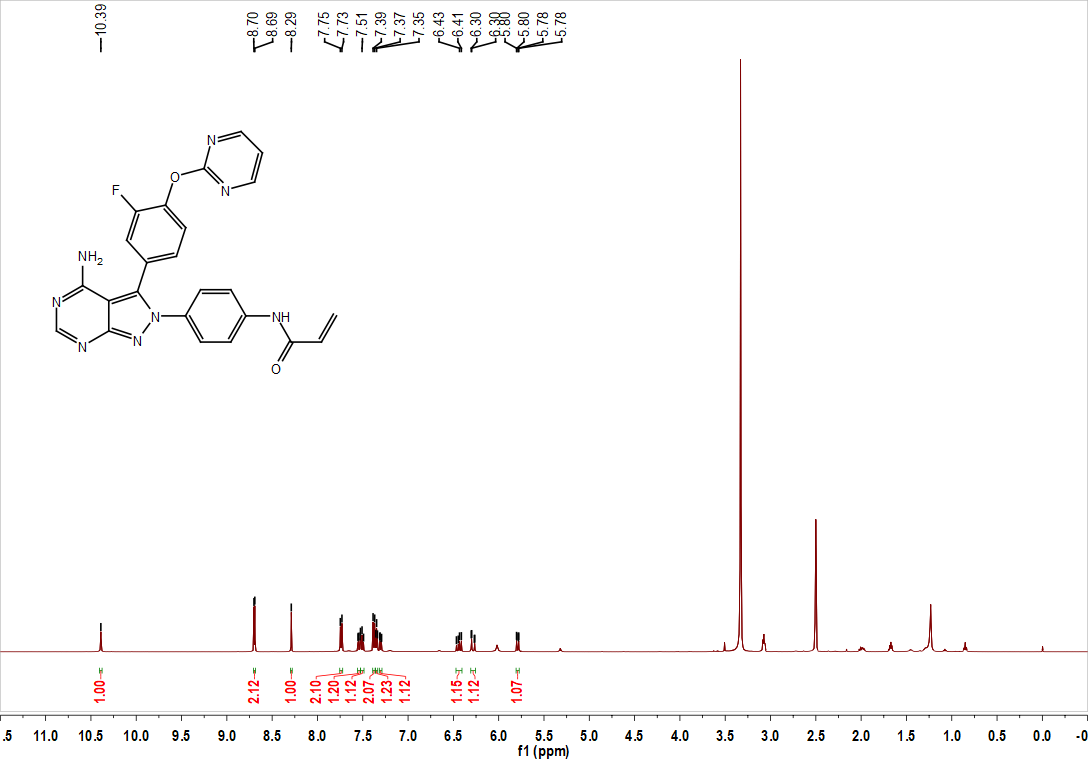


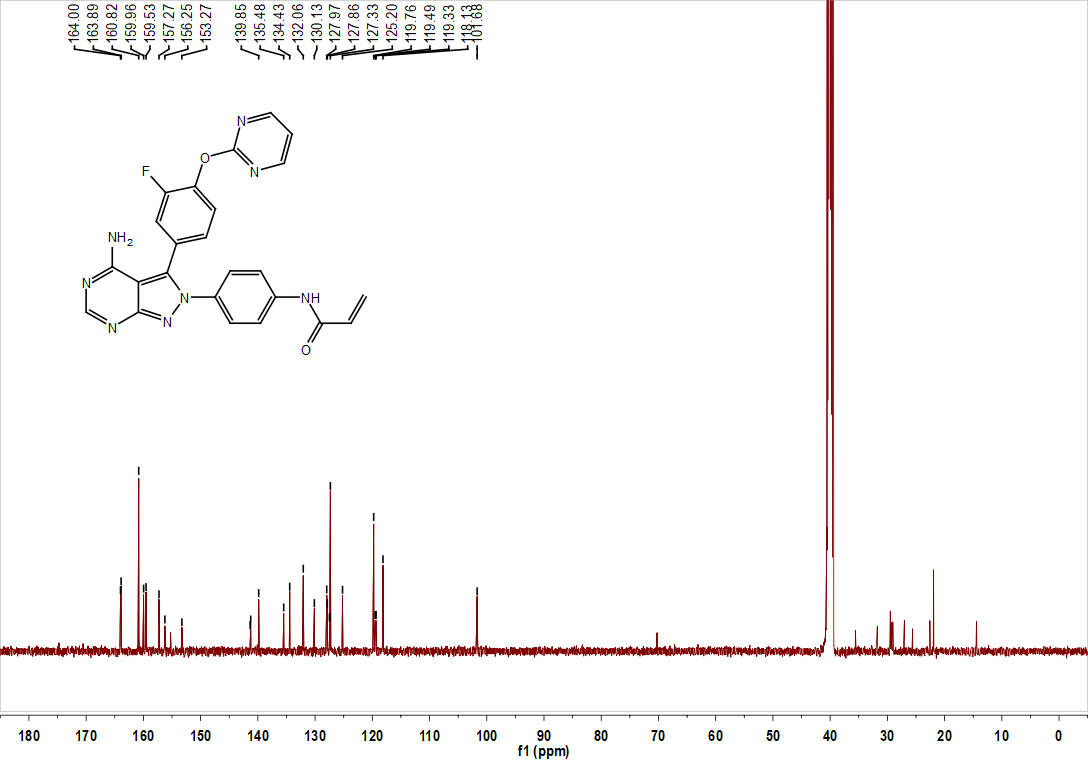


^1^H NMR and ^13^C NMR spectra for **PLW13**


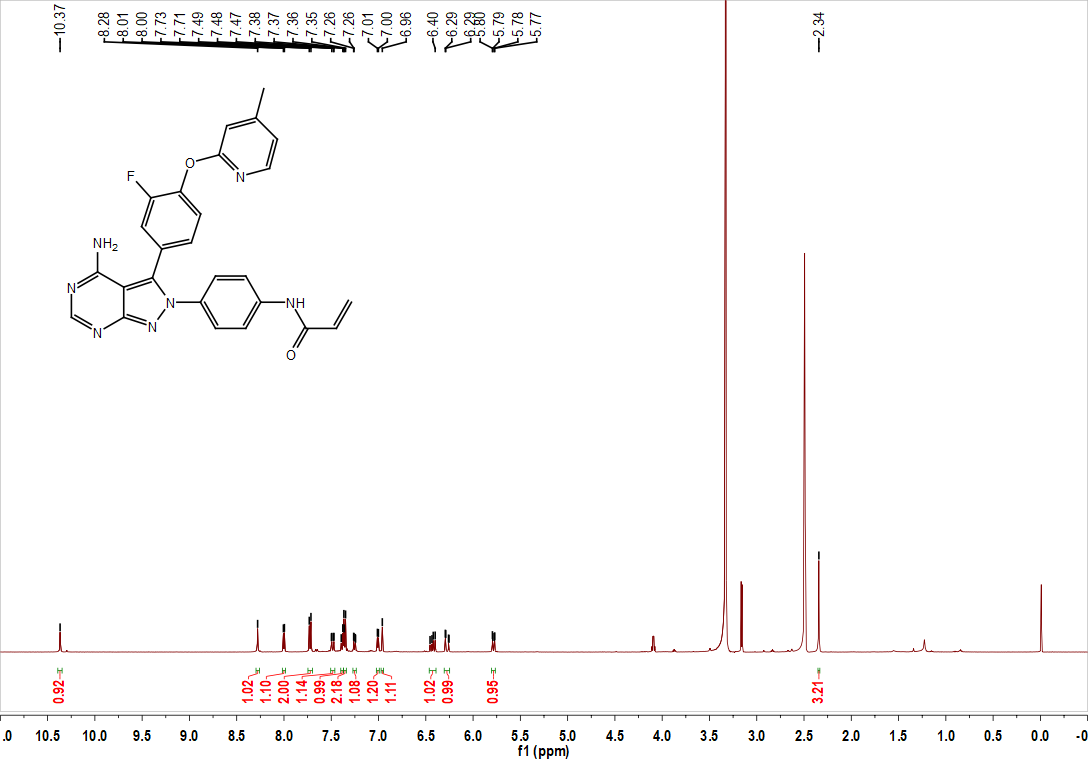


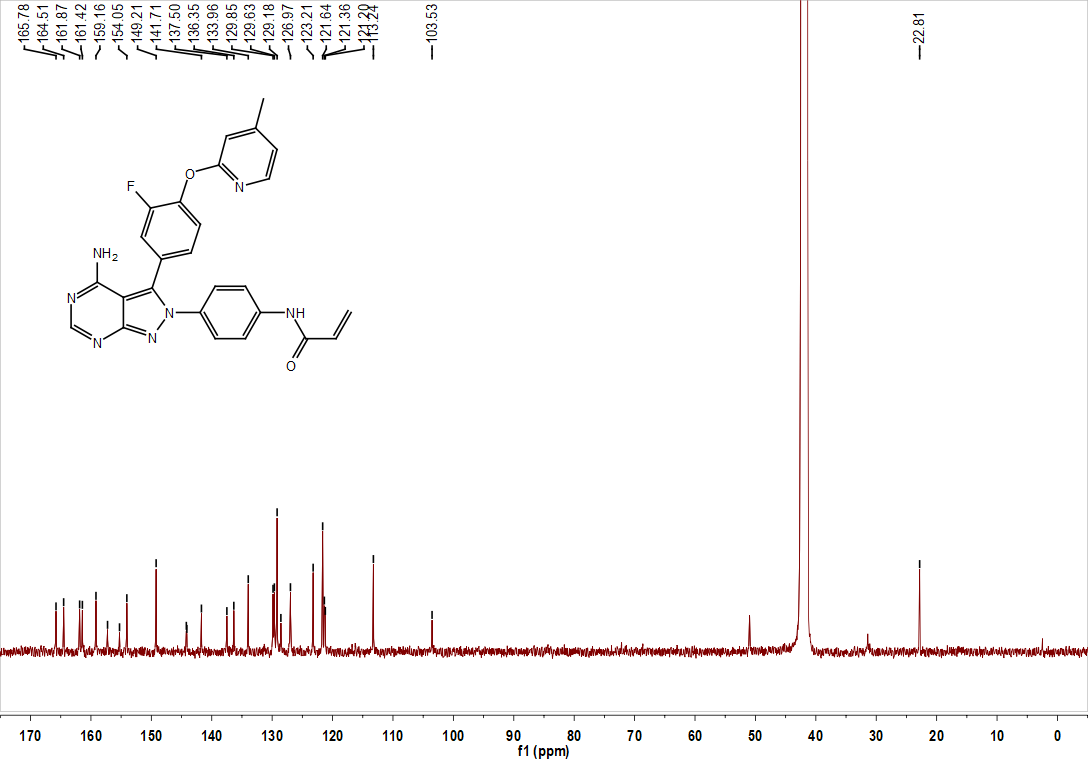


^1^H NMR and ^13^C NMR spectra for **PLW14**


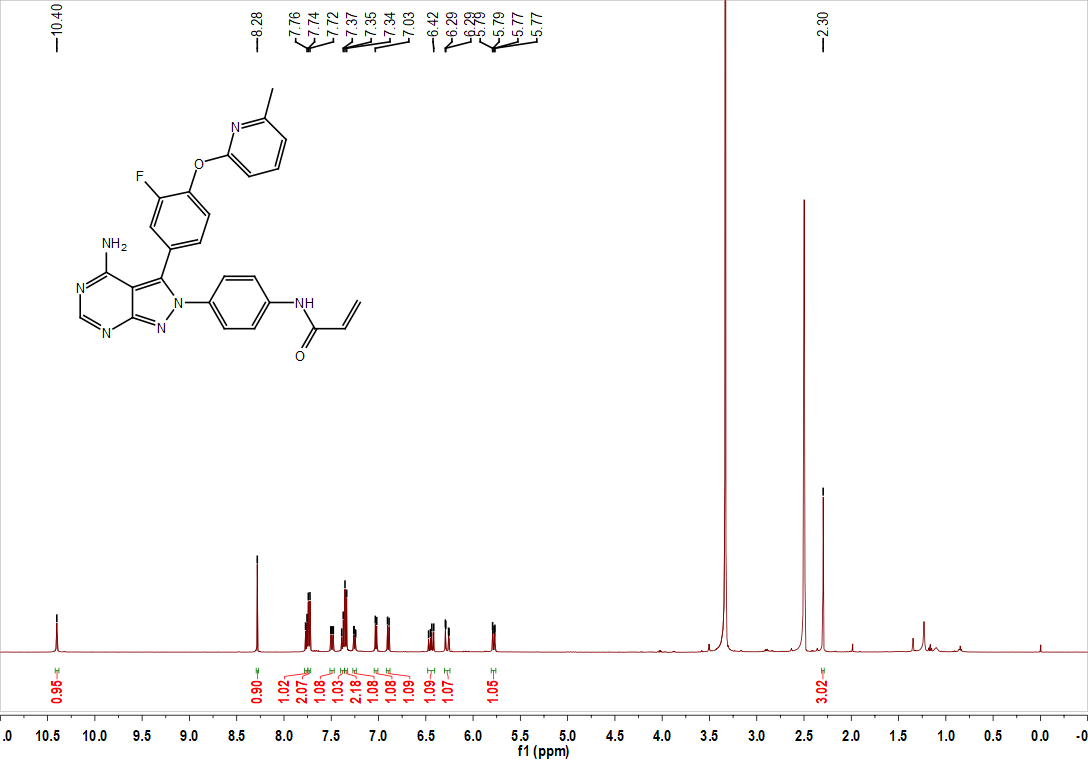


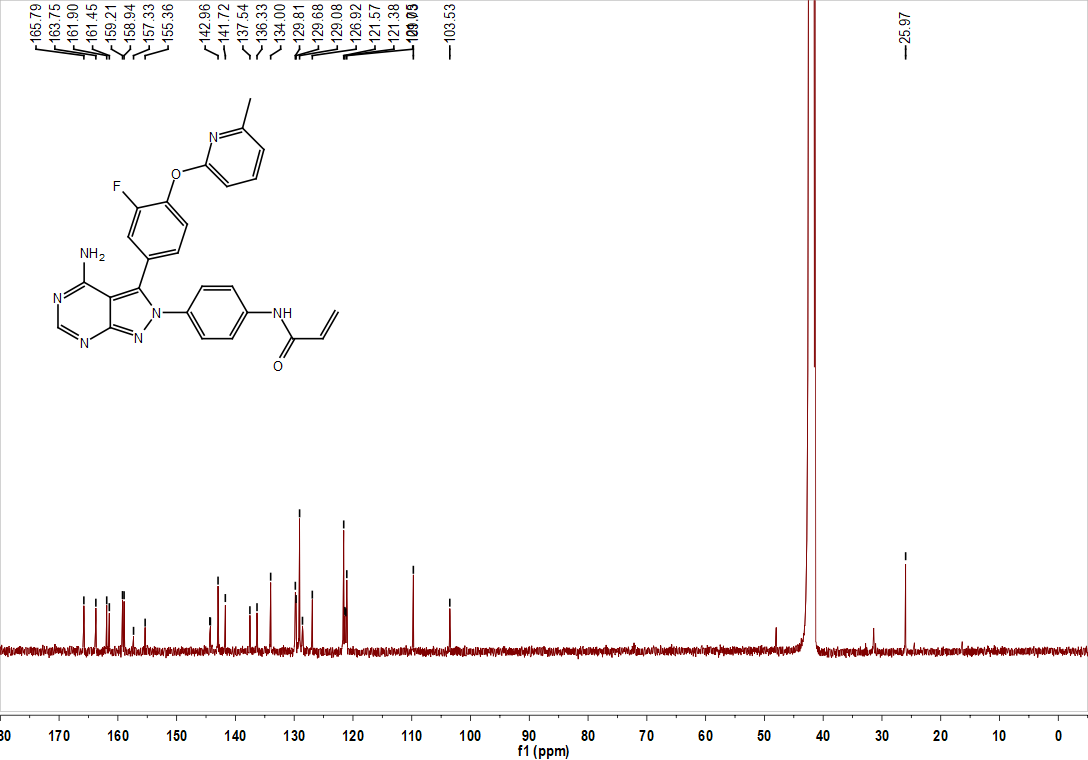


^1^H NMR and ^13^C NMR spectra for **PLW14N**


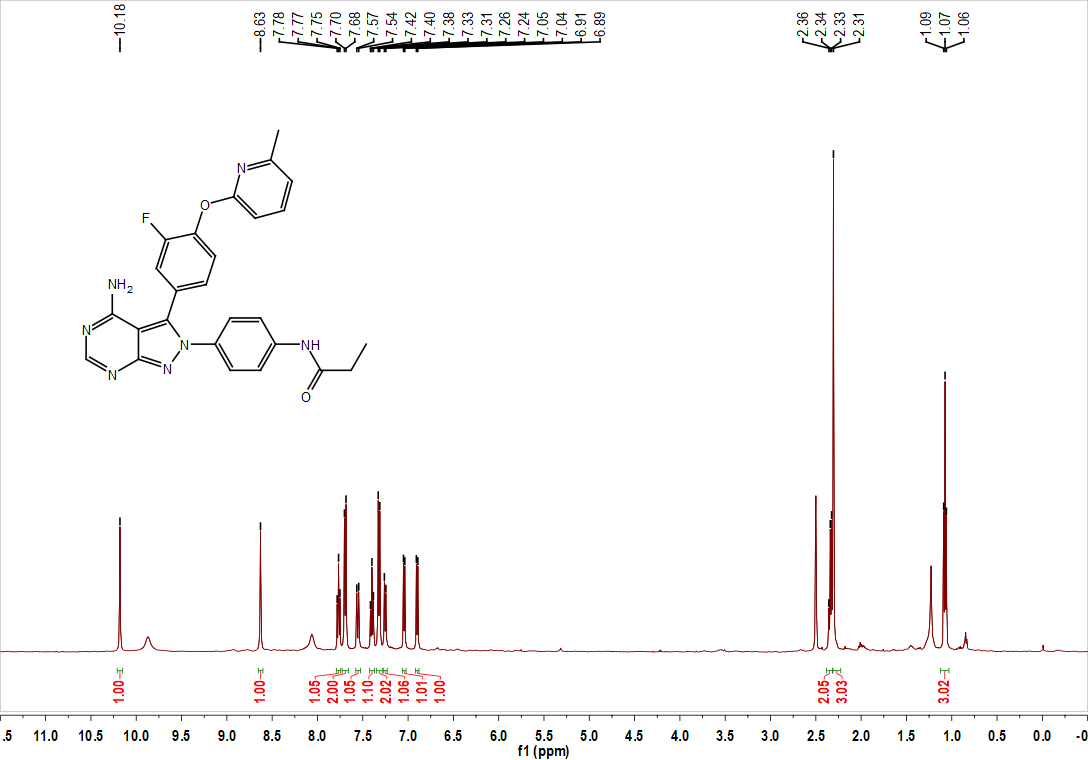


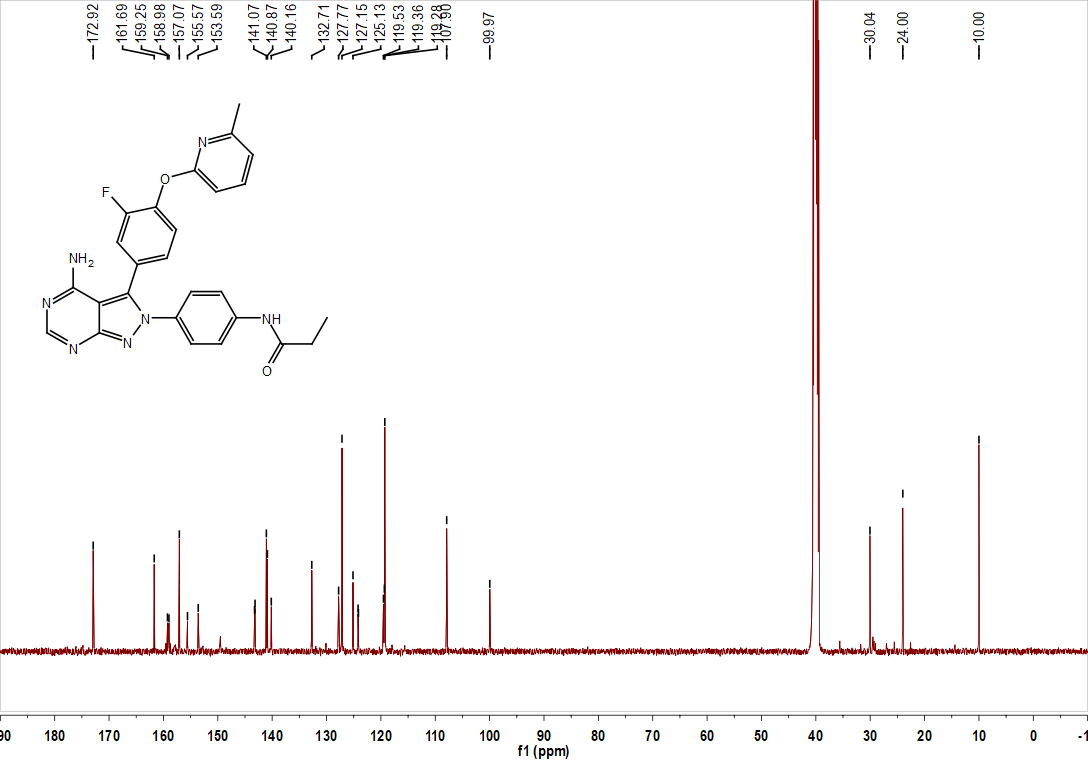


**HPLC spectra of** all inhibitors

HPLC Purity Analysis of **PLW1**


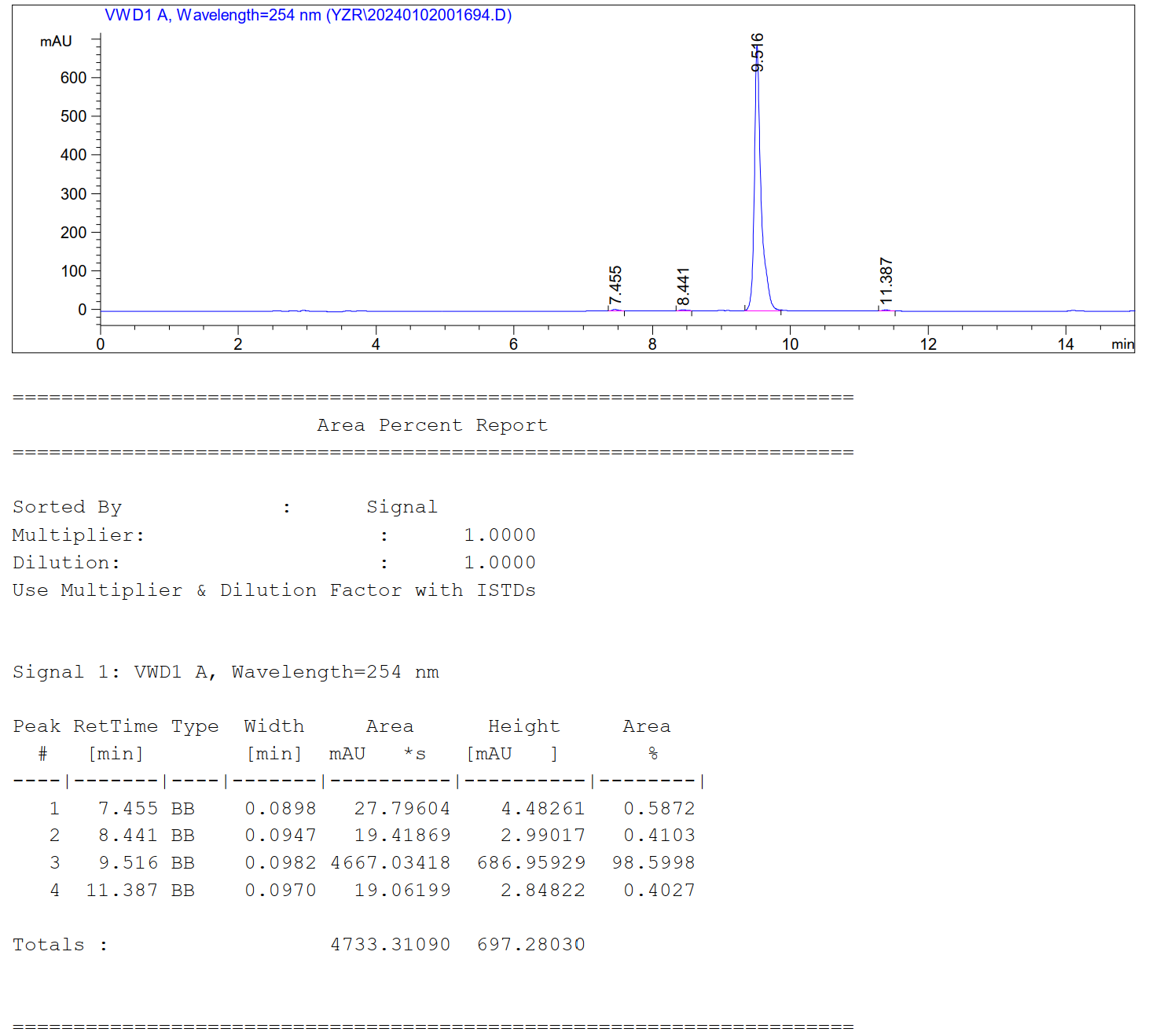


HPLC Purity Analysis of **PLW2**


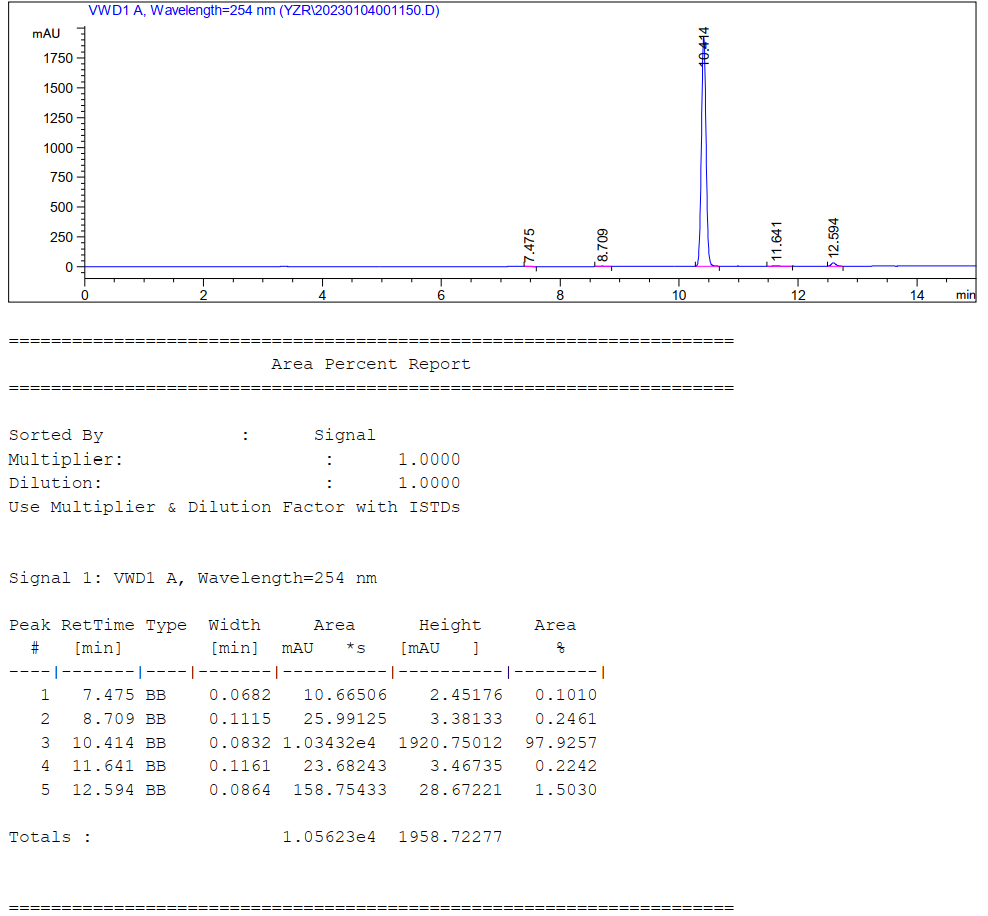


HPLC Purity Analysis of **PLW3**


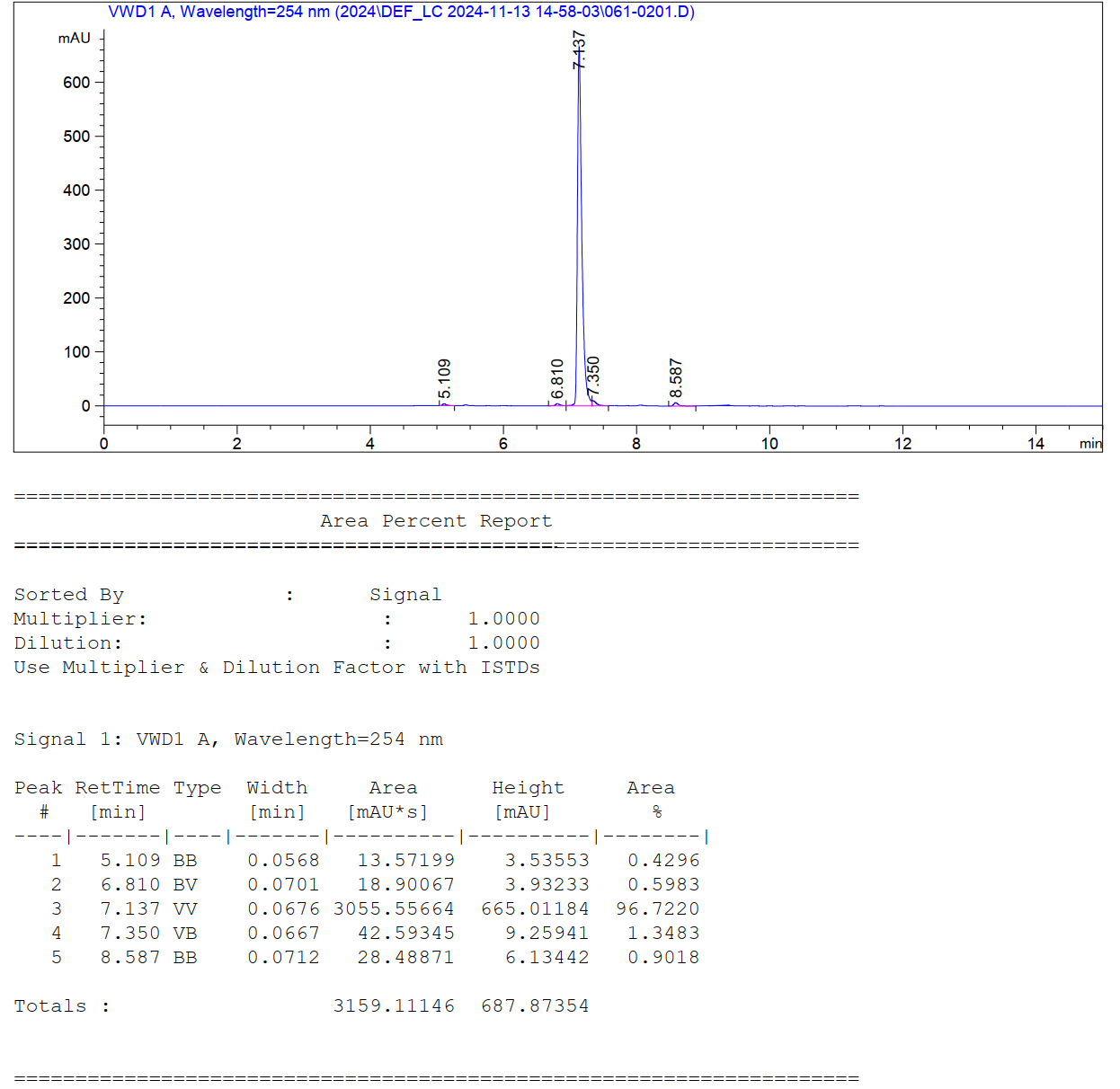


HPLC Purity Analysis of **PLW4**


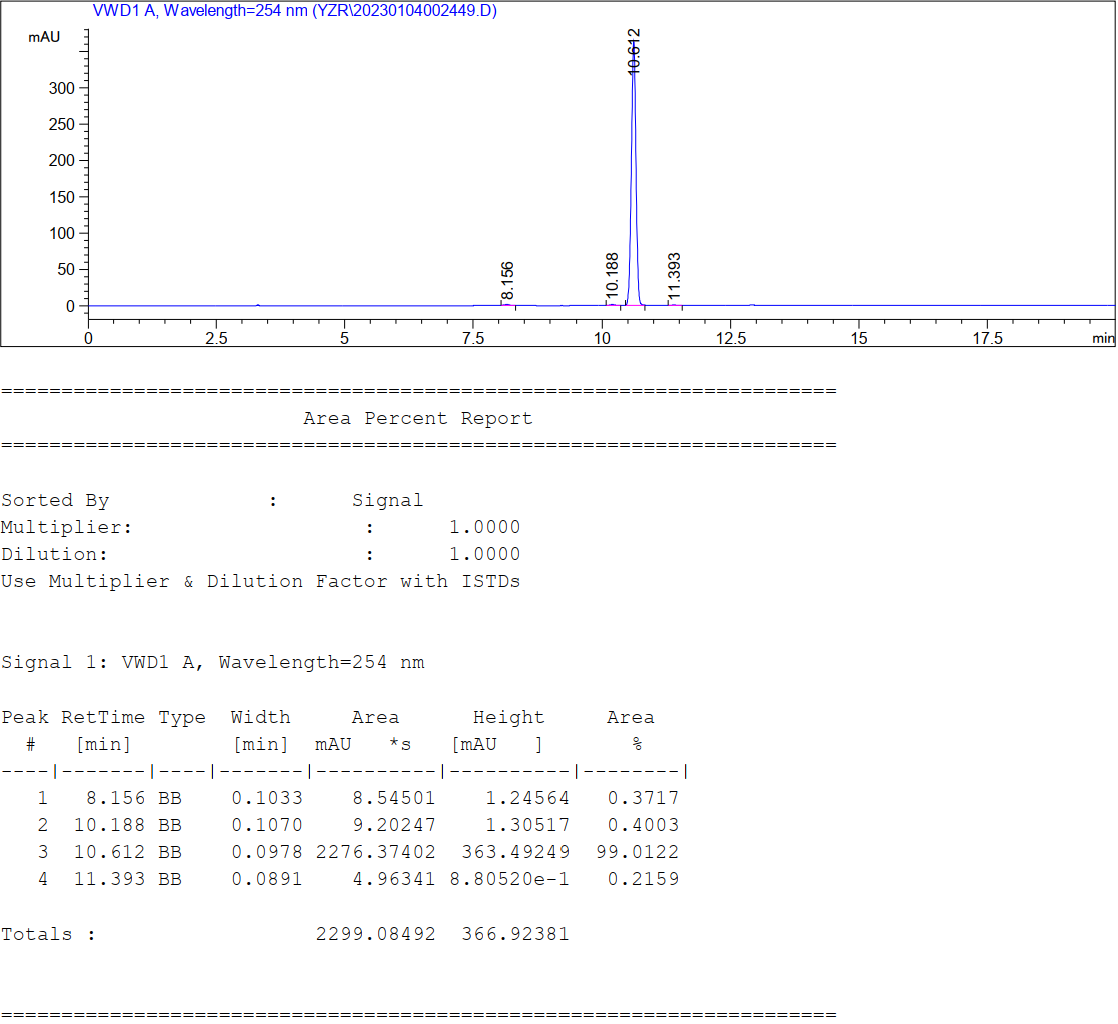


HPLC Purity Analysis of **PLW5**


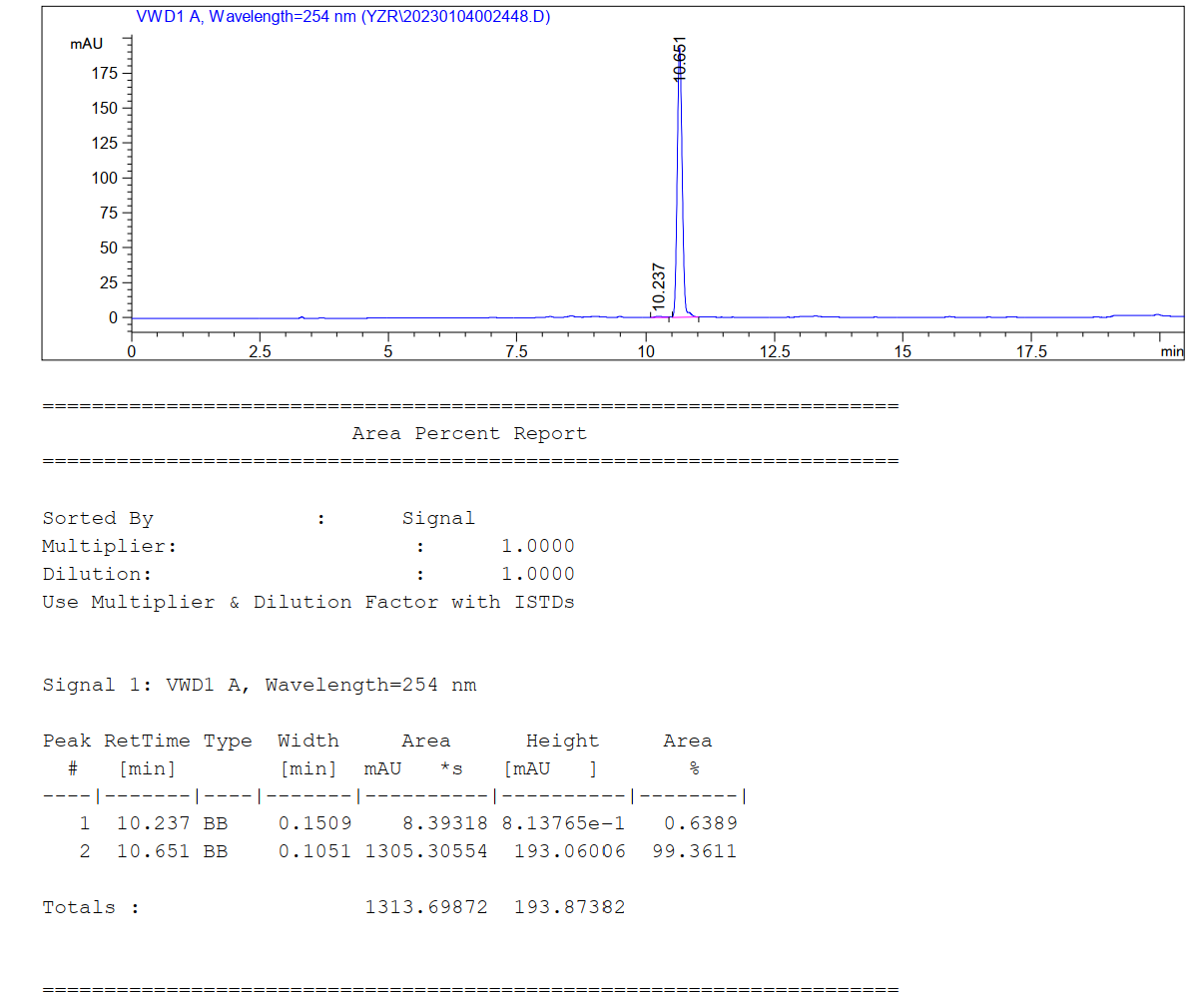


HPLC Purity Analysis of **PLW6**


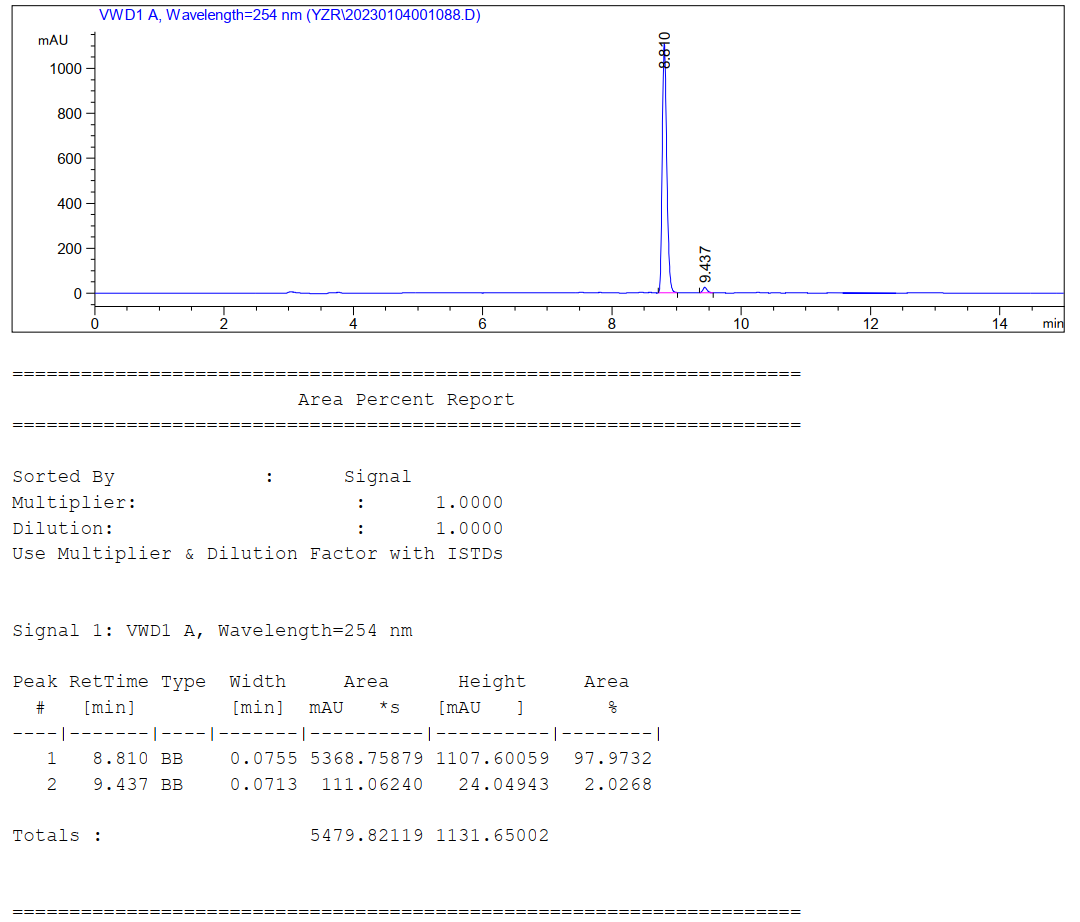


HPLC Purity Analysis of **PLW7**


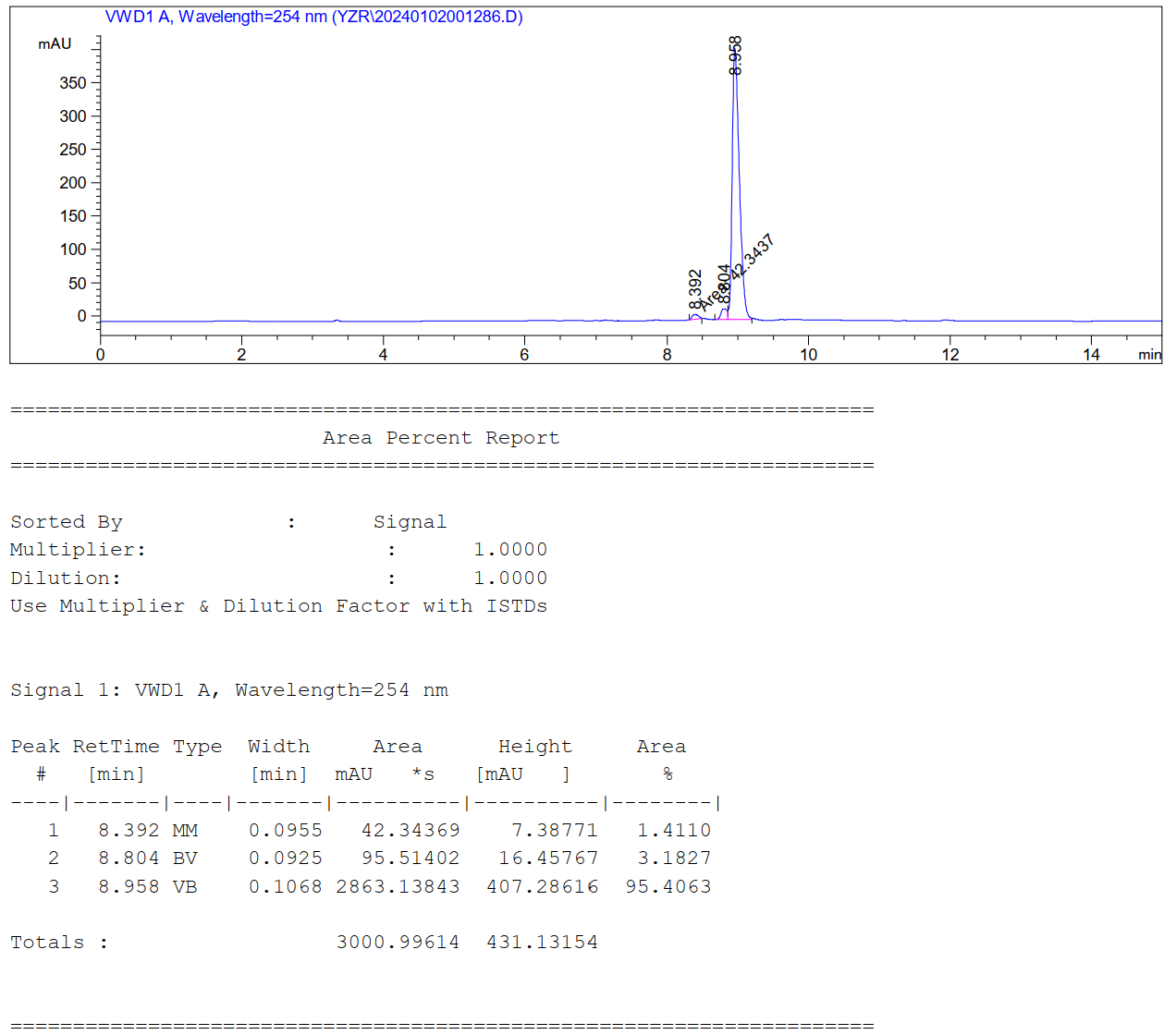


HPLC Purity Analysis of **PLW8**


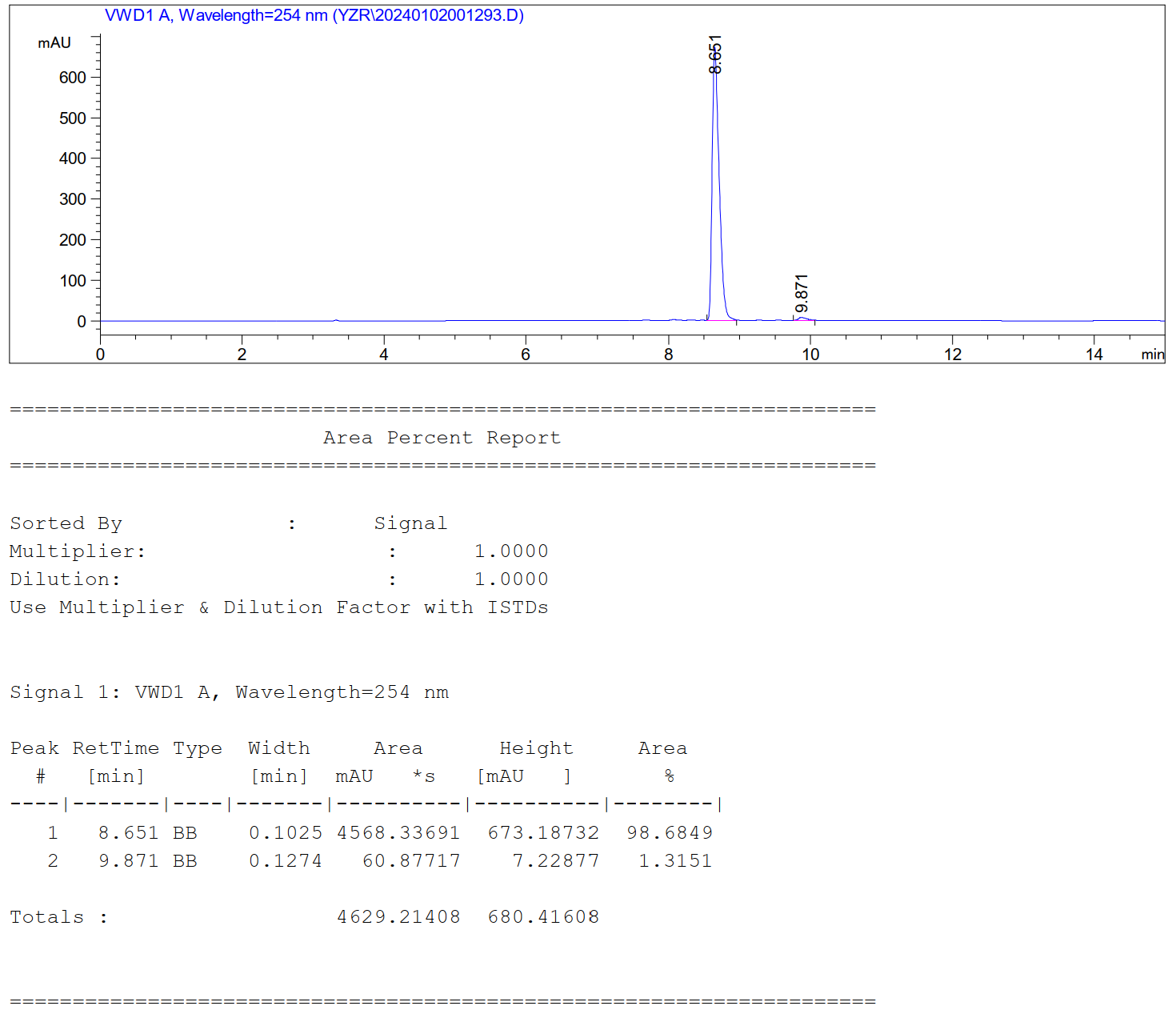


HPLC Purity Analysis of **PLW8**


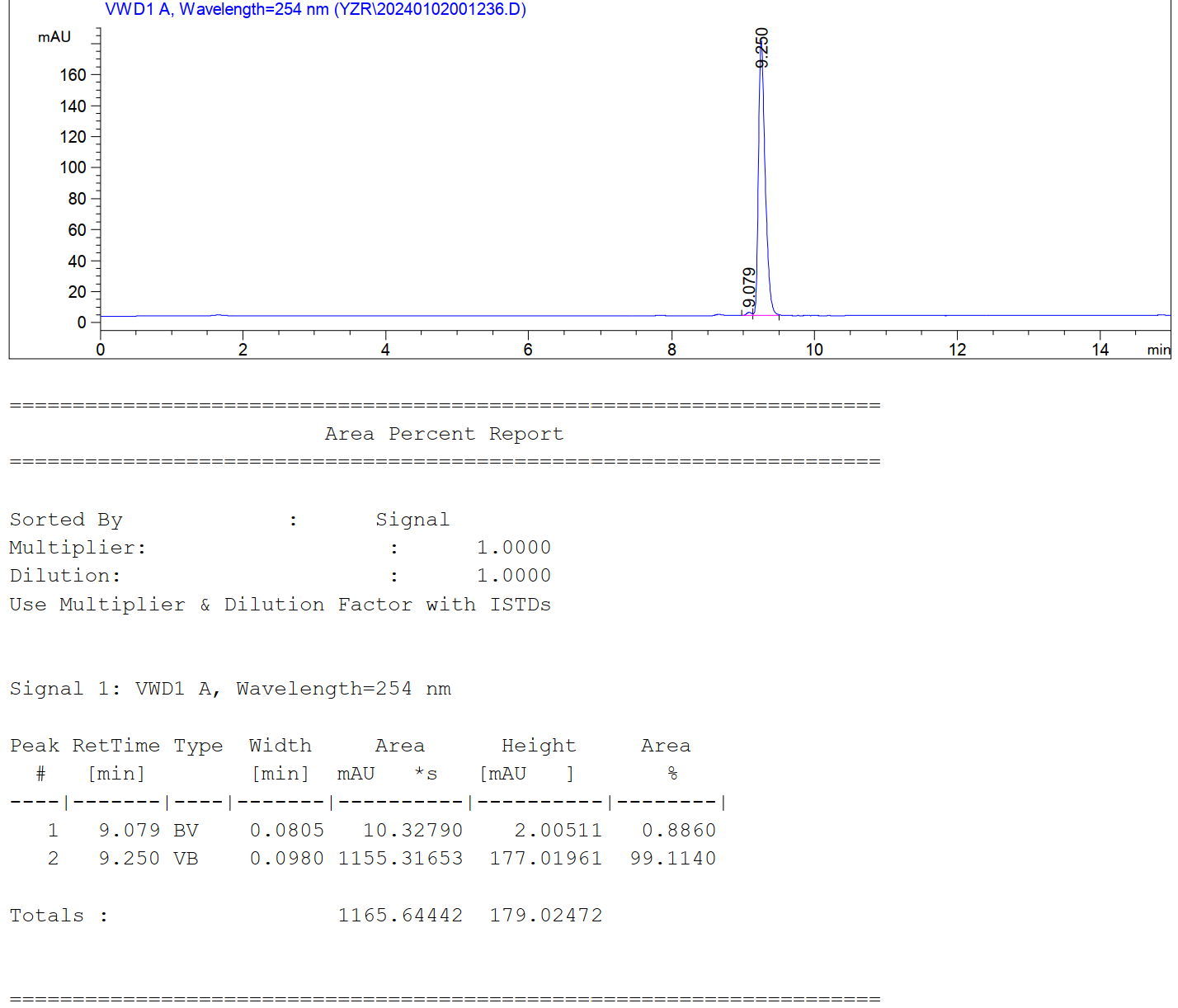


HPLC Purity Analysis of **PLW10**


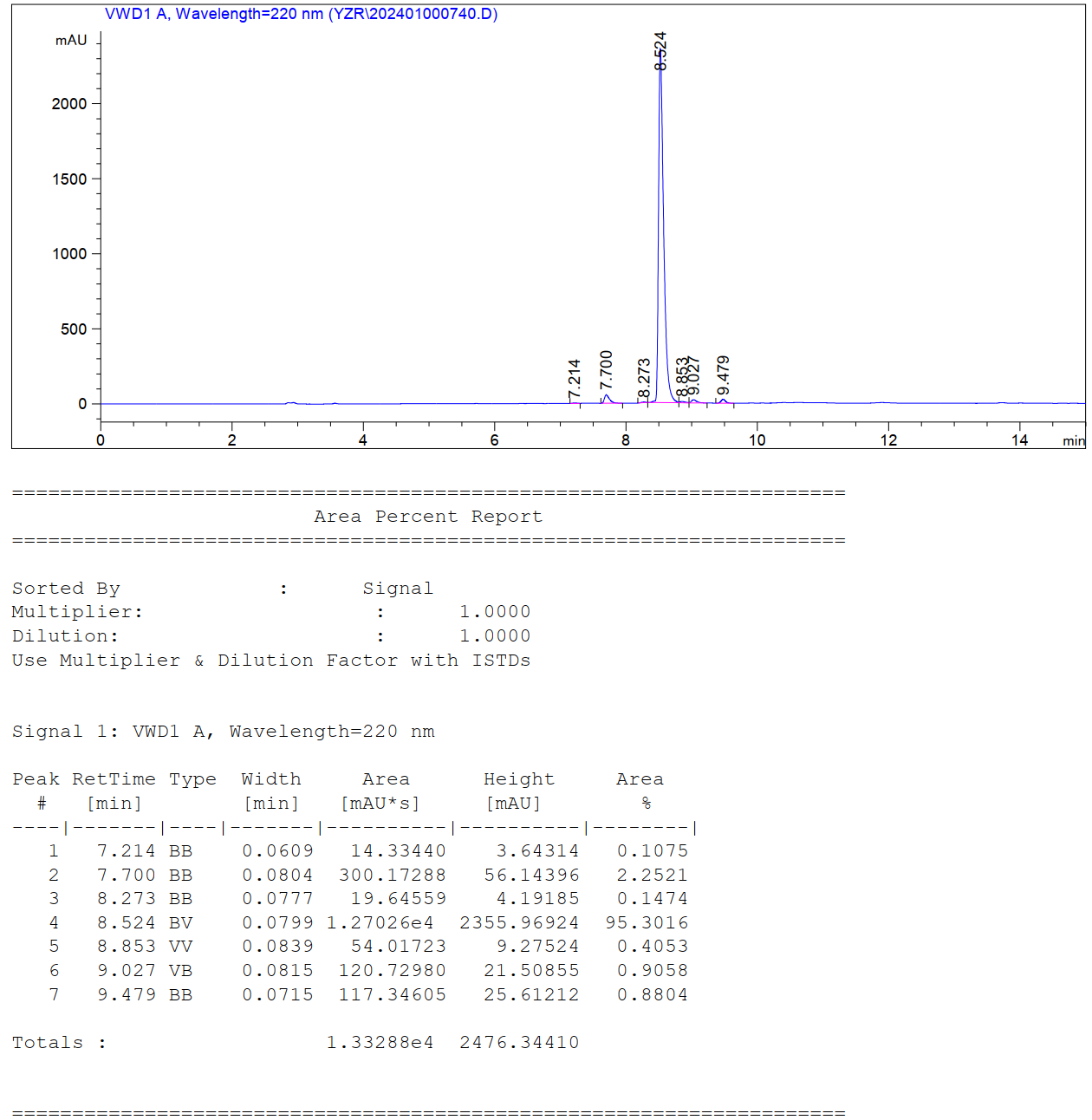


HPLC Purity Analysis of **PLW11**


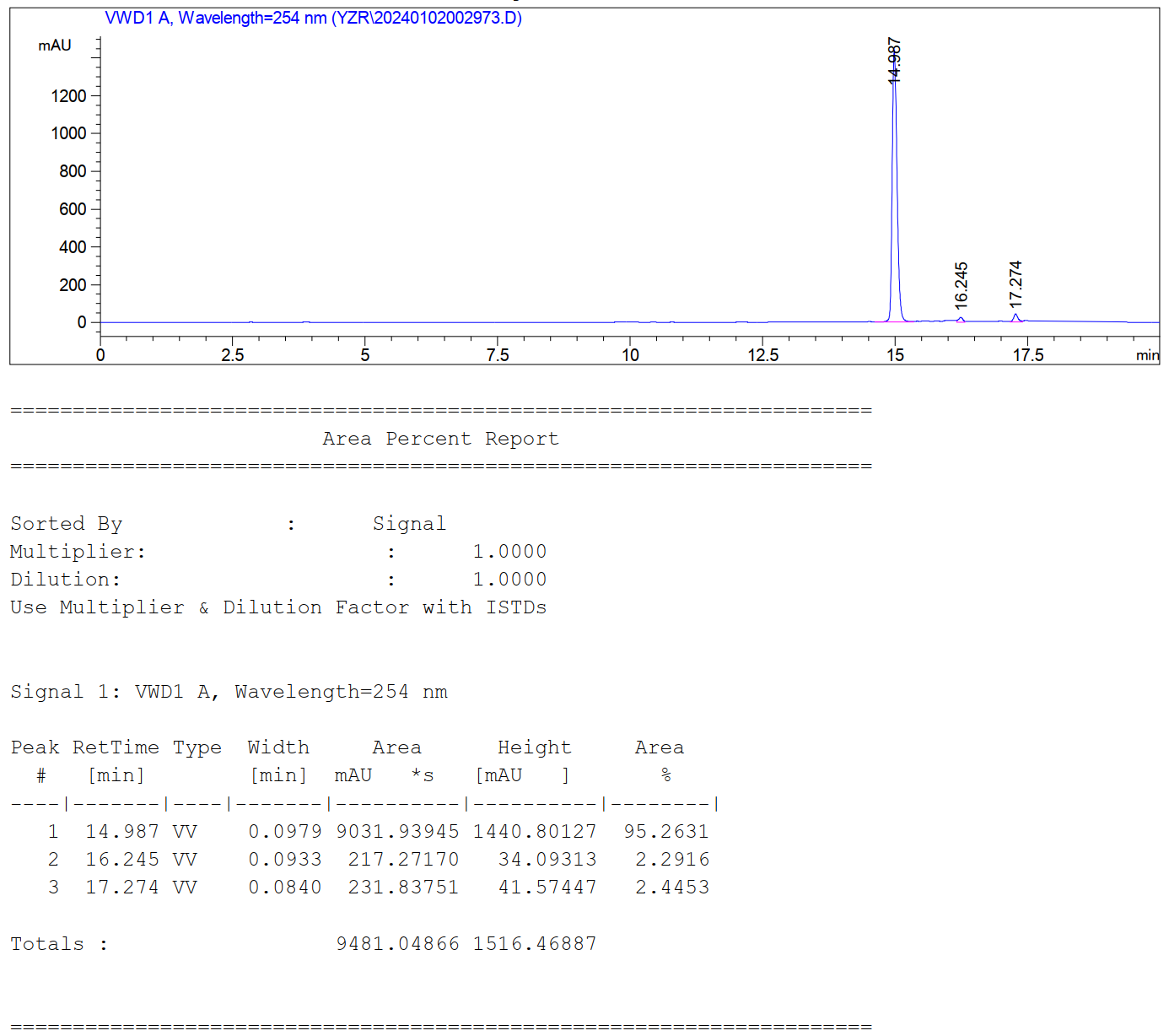


HPLC Purity Analysis of **PLW12**


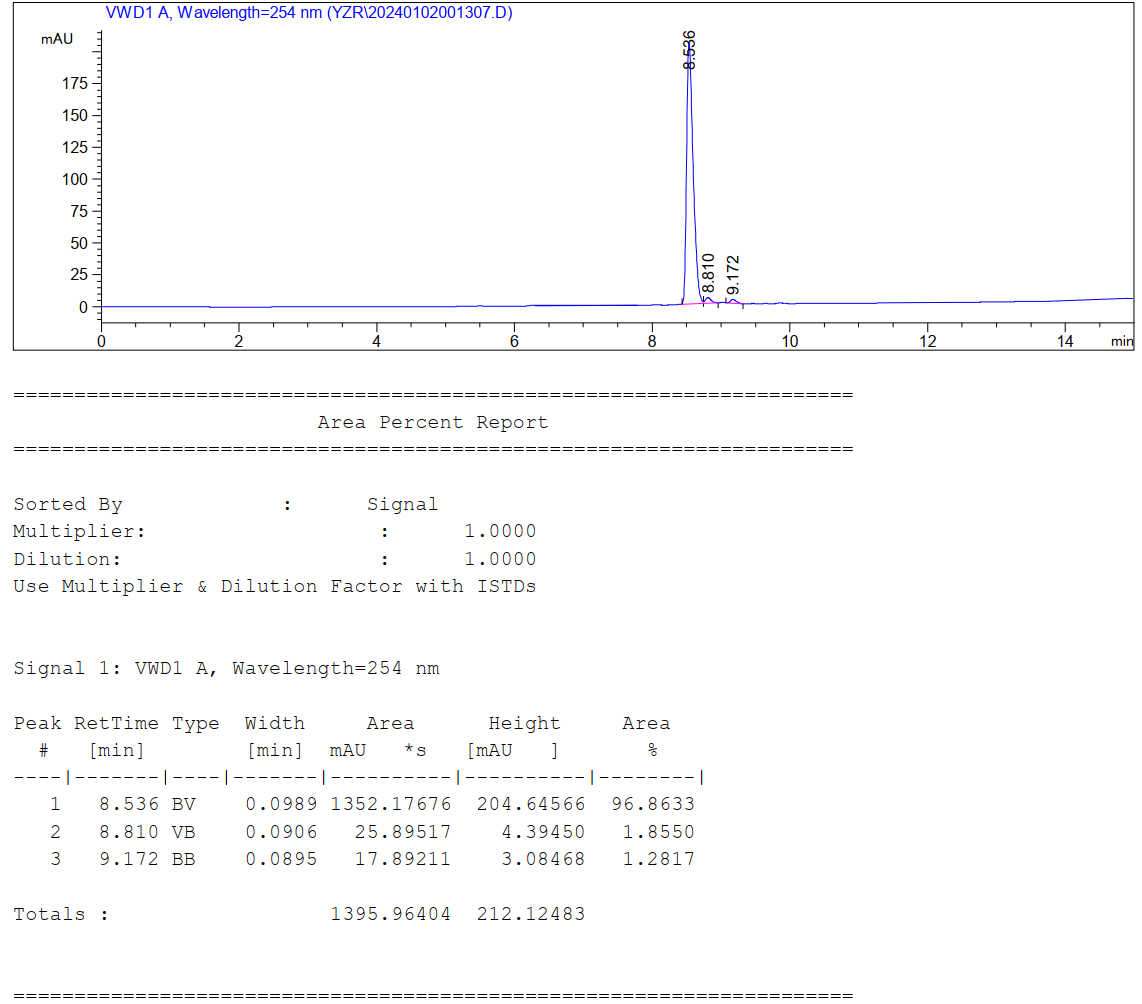


HPLC Purity Analysis of **PLW13**


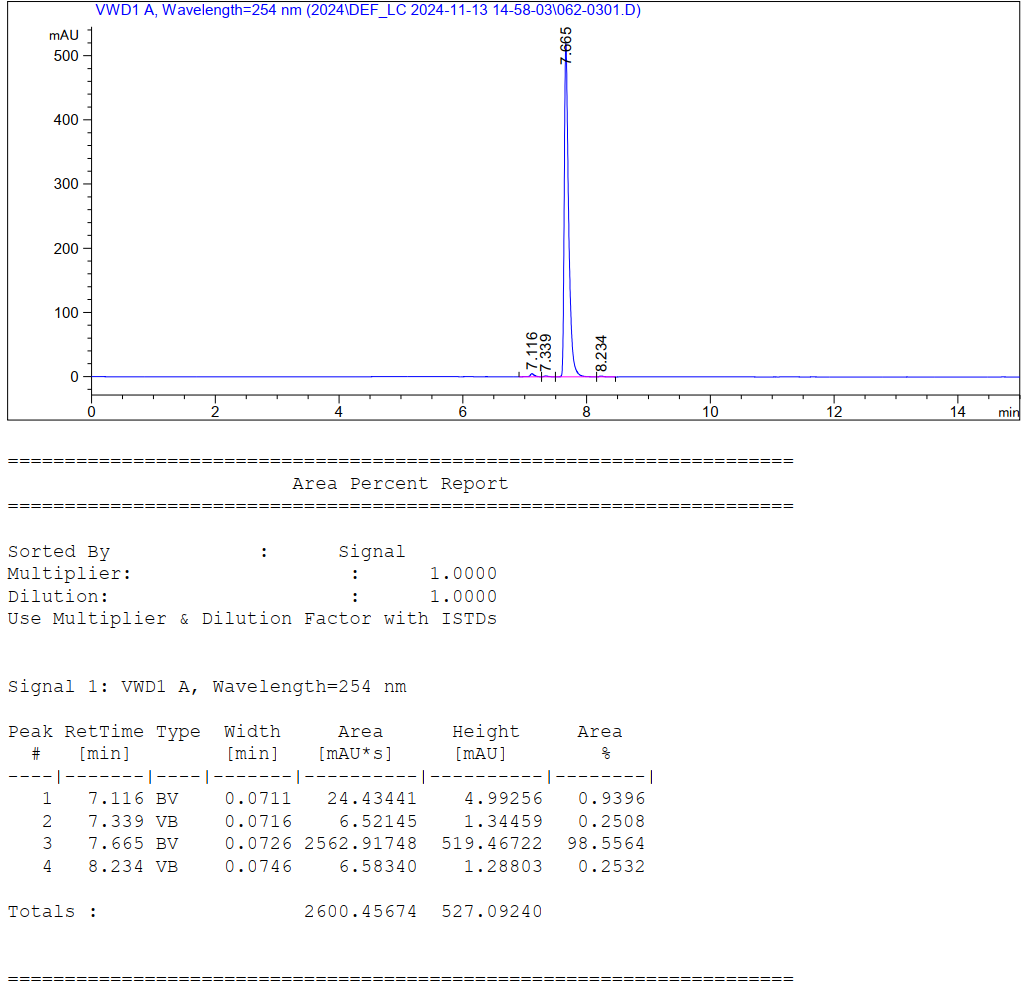


HPLC Purity Analysis of **PLW14**


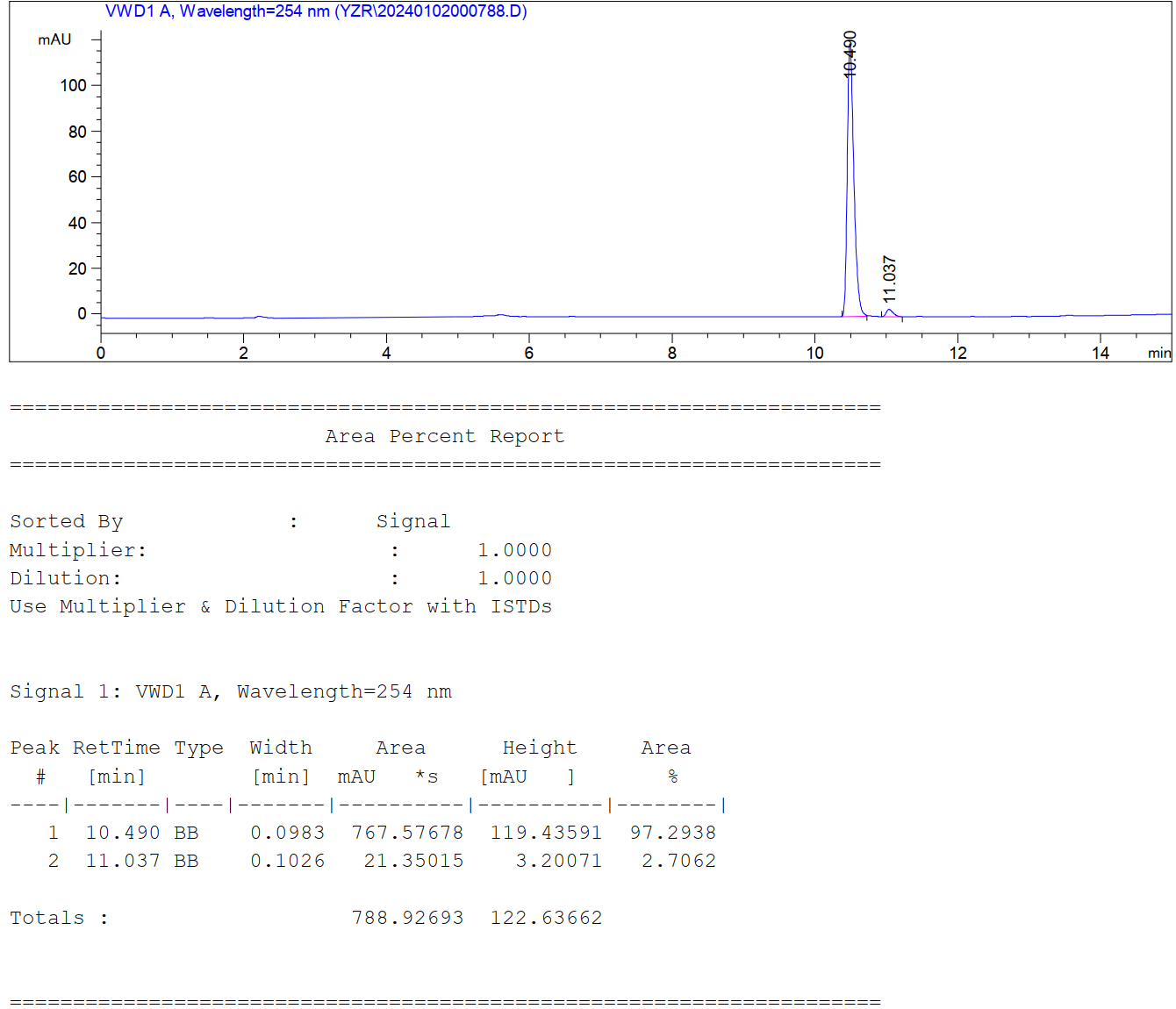


HPLC Purity Analysis of **PLW14N**


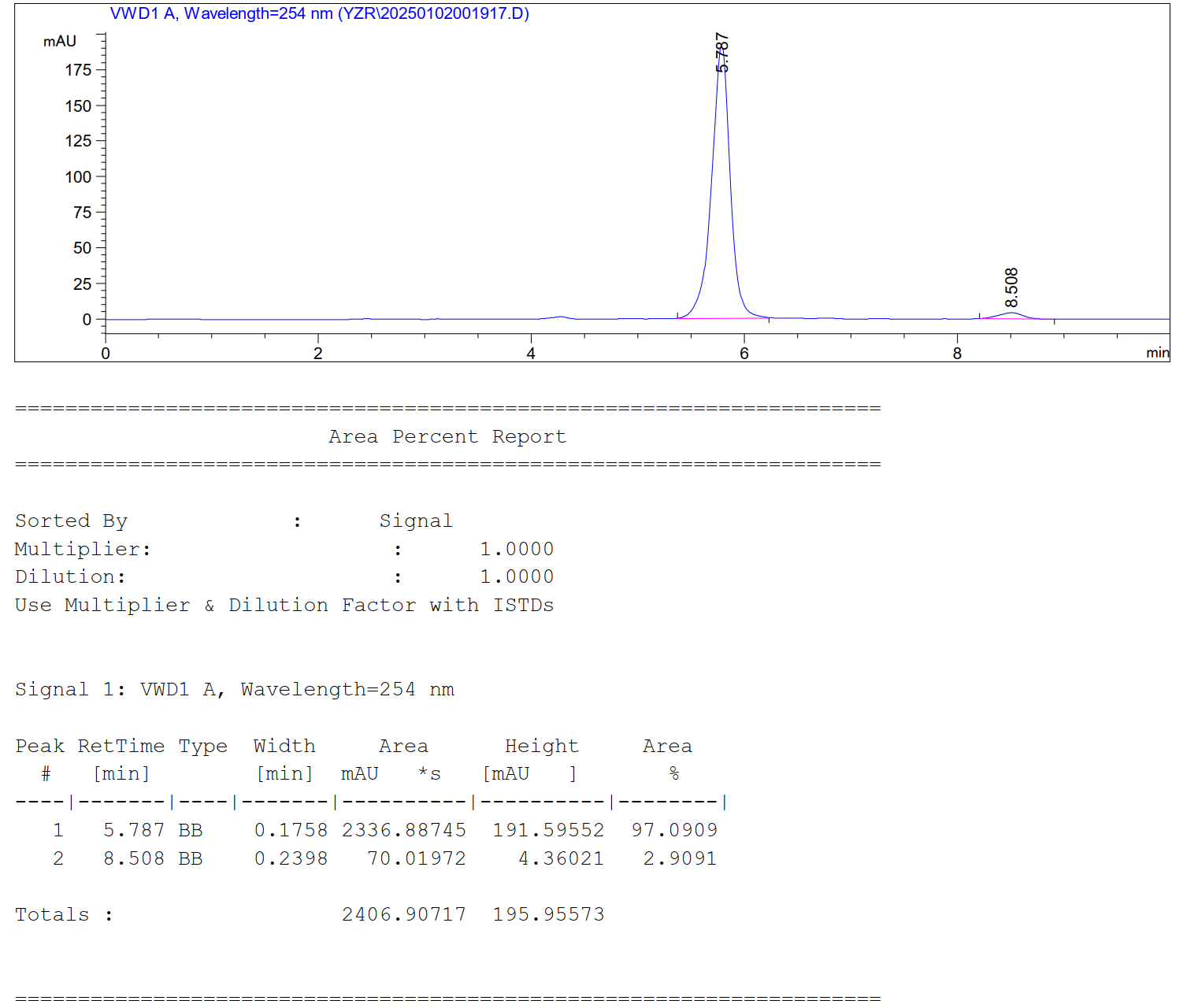

Supplement: SI without authors.docx [file IENZ_A_2647526_SM3513.docx]
